# Supplementary material for: De novo biosynthesis of simple aromatic compounds by an arthropod (Archegozetes longisetosus)
Source: Proc Biol Sci. 2020 Sep 2;287(1934):20201429. doi: 10.1098/rspb.2020.1429 (PMC7542773; doi:10.1098/rspb.2020.1429)
Supplement: Figure S1 [file rspb20201429supp1.pdf]

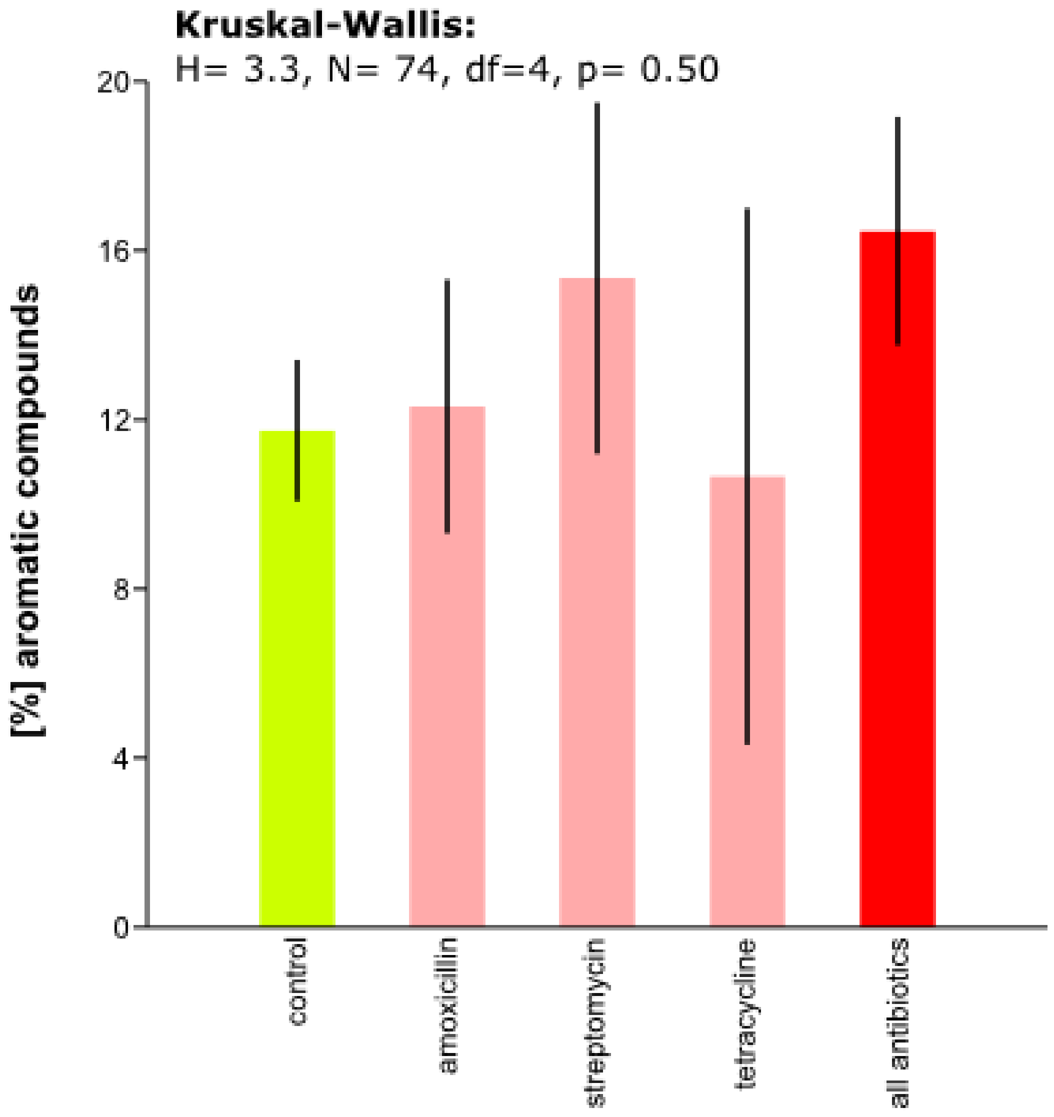

Figure S1. The effect of different antibiotic treatments - amoxicillin, streptomycin, tetracycline, and a combination of all antibiotics - on the relative aromatic compound ion abundances (%; calculated from ion abundances) compared to the control group. Coloured bars represent means, error indicators are standard errors. In-figure text denotes the result and statistical parameters of a non-significant Kruskal-Wallis test. df= degrees of freedom; N= sample size.
