# Supplementary material for: De novo biosynthesis of simple aromatic compounds by an arthropod (Archegozetes longisetosus)
Source: Proc Biol Sci. 2020 Sep 2;287(1934):20201429. doi: 10.1098/rspb.2020.1429 (PMC7542773; doi:10.1098/rspb.2020.1429)
Supplement: Figure S2 [file rspb20201429supp2.pdf]

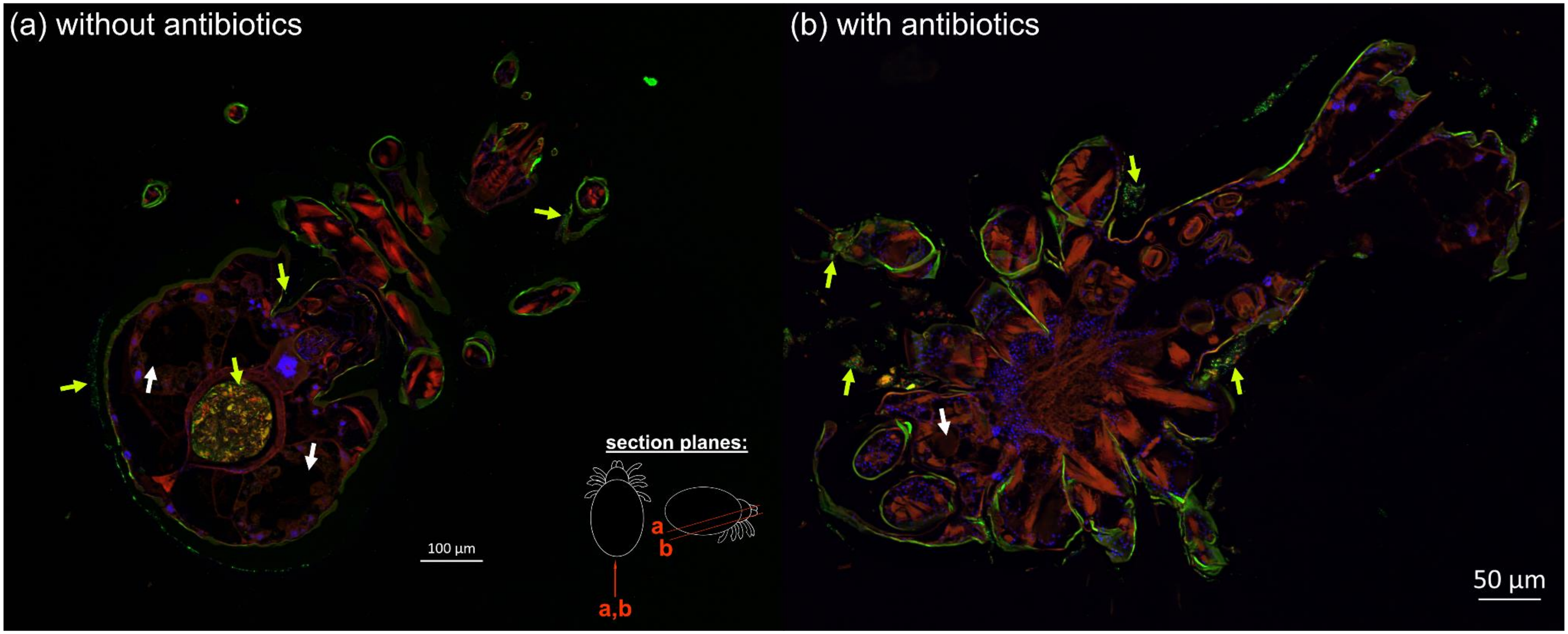

Figure S2. Fluorescence *in situ* hybridization (FISH) focusing on the cuticular surface of *Archegozetes longisetosus*. The left side of the figure (a) shows a frontal section of a mite fed with untreated wheat-grass powder. Bacterial signals (green arrow heads) were recovered on the cuticle (see four example arrows) and also on/within a faecal pellet in the alimentary tract. Yet, no signals were detected in the caecae (white arrow heads). The right side (b) shows another frontal plane (more ventrally), from a mite treated with a mixture of antibiotics (10% w/w; combined amoxicillin, streptomycin and tetracycline; oral in the diet). No bacterial signals were recovered from the alimentary tract (white arrowhead). However, signals were still detected on the cuticle (green arrow heads). Bacteria stained in green with the general bacterial probe EUB338-Cy5; note the different scale bars.
