# Supplementary material for: De novo biosynthesis of simple aromatic compounds by an arthropod (Archegozetes longisetosus)
Source: Proc Biol Sci. 2020 Sep 2;287(1934):20201429. doi: 10.1098/rspb.2020.1429 (PMC7542773; doi:10.1098/rspb.2020.1429)
Supplement: Figure S3 [file rspb20201429supp3.pdf]

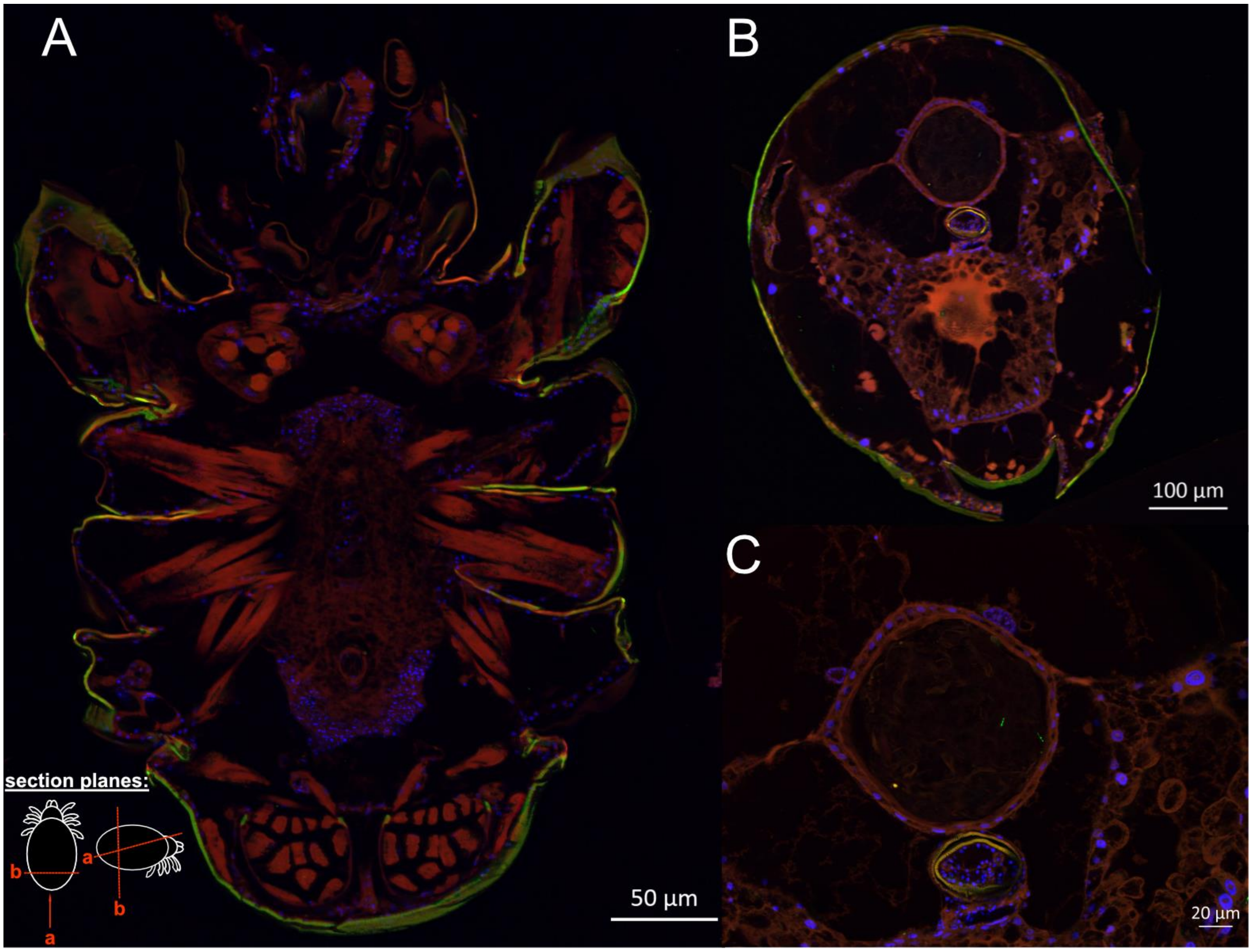

Figure S3. Fluorescence *in situ* hybridization (FISH) focusing on the detection of fungi in *Archegozetes longisetosus*. The left side of the figure (a) shows a frontal section of a mite fed with untreated wheat-grass powder. No fungal signals were detected. The top right side (b) shows transversal plane and the bottom right side (c) shows a magnification of the alimentary tract already depicted in (b), from a mite treated with a mixture of antibiotics (10% w/w; combined amoxicillin, streptomycin and tetracycline; oral in the diet). Two signals were detected within food particles in the alimentary tract. Fungi are stained in green with the general fungal probe PF2-Cy5; note the different scale bars.
