# Supplementary material for: De novo biosynthesis of simple aromatic compounds by an arthropod (Archegozetes longisetosus)
Source: Proc Biol Sci. 2020 Sep 2;287(1934):20201429. doi: 10.1098/rspb.2020.1429 (PMC7542773; doi:10.1098/rspb.2020.1429)
Supplement: Figure S4 [file rspb20201429supp4.pdf]

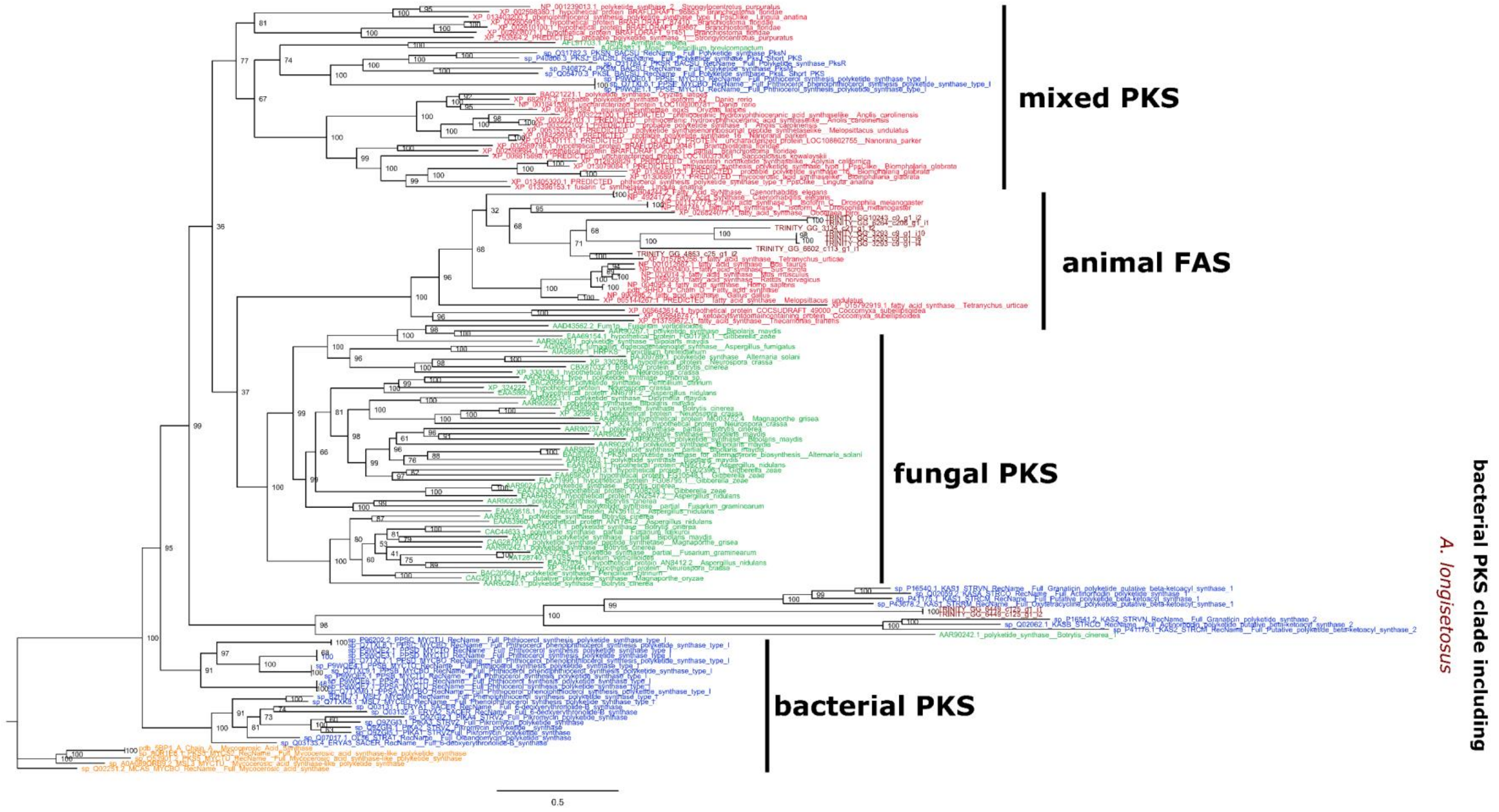

Figure S4. Full maximum likelihood tree based on an alignment of the KS- domains of fatty-acid and polyketide synthases from animals (including those of the mite – depicted in dark red), fungi and bacteria. Bootstrap values (based on 1,000 replicates) are indicated along branches and the scale bar below the tree denotes substitutions per site. The tree was rooted by the outgroup mycocerosic acid synthase from *Mycobacterium bovis*.
