# Supplementary material for: De novo biosynthesis of simple aromatic compounds by an arthropod (Archegozetes longisetosus)
Source: Proc Biol Sci. 2020 Sep 2;287(1934):20201429. doi: 10.1098/rspb.2020.1429 (PMC7542773; doi:10.1098/rspb.2020.1429)
Supplement: Figure S5 [file rspb20201429supp5.pdf]

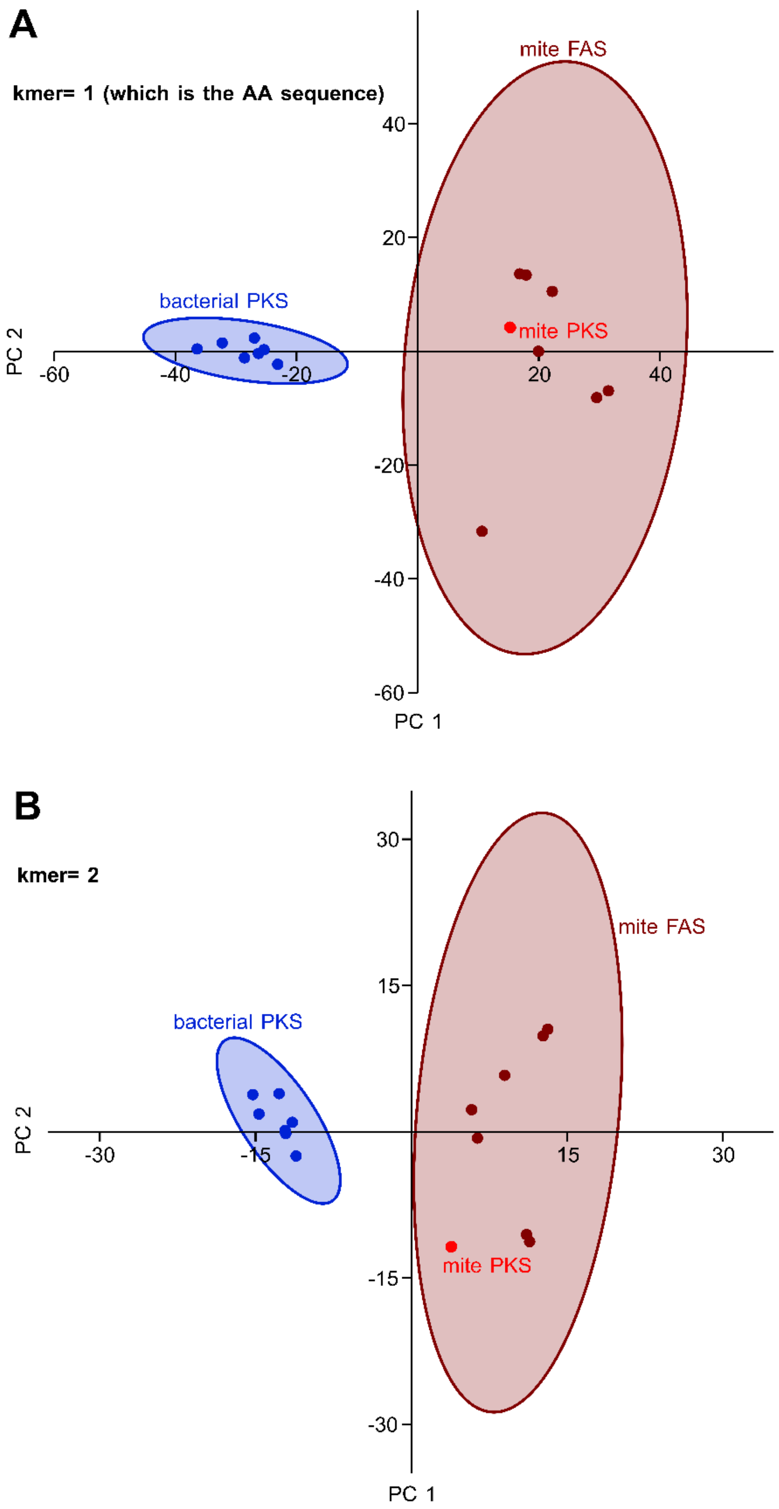

Figure S5. Principle component analyses of the kmer-counting matrices for (a) kmer=1, which equals the amino acid sequence and (b) kmer=2. In both cases the mite sequences including the mite polyketide synthase (red; PKS) were distinctly different from the bacterial PKS (blue). Ellipses represent the 95% confidence.
