# Supplementary material for: De novo biosynthesis of simple aromatic compounds by an arthropod (Archegozetes longisetosus)
Source: Proc Biol Sci. 2020 Sep 2;287(1934):20201429. doi: 10.1098/rspb.2020.1429 (PMC7542773; doi:10.1098/rspb.2020.1429)
Supplement: Table S1 [file rspb20201429supp6.pdf]

Table S1. Enrichment factors of the respective ions in treatment groups compared to the control calculated based on mass spectrometry. The mass/charge ratios (*m/z*) 136 and 150 are the molecular ions of 2-hydroxy-6-methyl-benzaldehyde and 3-hydroxybenzene-1,2-dicarbaldehyde, respectively and thus showed no enrichment.

m/z	**[$^{13}C_6$, d_7] D-glucose**	**[$^{13}C_3$] malonic acid**	**sodium [$^{13}C_1$] acetate**	**[$^{13}C_6$] phenylalanine**
**2-hydroxy-6-methyl-benzaldehyde**				
136 [M^+]	0	0	0	0
137	8	5	3	3
138	62	38	21	19
139	180	60	17	23
140	269	72	3	23
141	94	25	13	3
142	87	23	14	2
143	86	18	15	1
144	47	17	8	<1
**3-hydroxybenzene-1,2-dicarbaldehyde**				
150 [M^+]	0	0	0	0
151	4	1	1	2
152	64	20	2	24
153	423	48	14	66
154	2112	151	143	252
155	1170	23	59	96
156	938	15	42	37
157	668	2	30	21
158	737	1	31	1
