# Supplementary material for: De novo biosynthesis of simple aromatic compounds by an arthropod (Archegozetes longisetosus)
Source: Proc Biol Sci. 2020 Sep 2;287(1934):20201429. doi: 10.1098/rspb.2020.1429 (PMC7542773; doi:10.1098/rspb.2020.1429)
Supplement: Table S2 [file rspb20201429supp7.pdf]

Table S2. Amino acid alignment of curated KS-domains of bacterial, fungal and animal PKS as well as animal FAS, including putative PKS/FAS from *A. longisetosus*. Sequences will be deposited in the NCBI database, and accession numbers provided upon acceptance of the manuscript.

>AAD43562.2_Fum1p__Fusarium_verticillioides_

-VAIVGMGMRLPGGIHTPDELWGMLVEKRST-RCEIPPTR-FSVDGFHSPSS--KPGSIAMR-HGHFLDDKD-
DLHRLDTSFFSMGMTEVSDIDPQQRMLLEVAYECMQS---SG-----QTNWRGSNIGCYVGVWGEDW-----LDLHSK--DLYDSGTYRVSGGHDF--AI-
SNRISYEYDLKGPSFTIKAGCSSSLIALHEAVRAIRAGDCDGAIVAGTNLVFSPTMSVA--MTEQ-----GVL-SPDASCKTFDAN---A-NGYARGEA-INAIFLKP--
LNNALREGDP-IRALVRATSSNSDGKTP-----GMSMPSSESHEALIRRAYGEVF---LD-PKD----TCFVEAHGTGTSVGDPLEATAIAR-VF----
GGSSDNKLYIGSVKPNLGHSEGASGVSSVMKAVLALENRTIPPNINFS-TP-NPKIP--FSEMNMAVPVDAIPWP------------
RDRPLRVSVNSFGIGGANAHCIIETLEE---YLGRSLPNESQ

>AAO62426.1_type_I_polyketide_synthase__Phoma_sp._C2932_

PIAIIGMSCRFPGNATSPEKLWELCAQGRSA-WSSIPKSR-FRQEGFYNPNAE-RVGTSHVV-GGHFLEE---
DPSLFDASFFNLSAEAAKTMDPQFRLQLESVYEAMES---AGIT---LEHIAGSDTSVYAGACFRDY-----HDSLVR--DPDLVPRFLLTGNG-A--
AMSSNRVSHFYDLRGASMTVDTGCSTTLTALHLACQGLRNRESKTSIVTGANVILNPDMFVT--MSSL-----GLL-GPEGKSHTFDAR---A-NGYGRGEG-
IATVIIKR--LDDALRAQDP-IRCIIRGTALNQDGRTAT-----LTSPSQTAQSDLIRACYRAAA---LD-PND----TAFLAAHGTGTRTGDAVEIAAAAD-VFGEKRSP--
ERPLWIGSVKTNIGHSEATSGLASVIQAALALEKGLIPPNINFK----EPNEKLGQVSAAVRVPSNLQKWPSVSG-----------
VRRASVNNFGYGGANAHVILESGIP--GHTPIANGS

>AAR85531.1_polyketide_synthase__Didymella_maydis_

PIAIVGLSFRGPGDATSAENLLRMVAESRES-RSPIPSQK-WNASGHYHPDPS-RQGS-HIVEYGHWFQQ---
DVYEFDAPFFNLSAVESAALDPQQRMLLECTYEAFEN---SGMP---LNKLVGTDTSVFTAVFCTDY-----TDMLWR--DPEMVPMYQCTNSG-
ATRANMANRVSYSFDLKGPSITVDTACSGGLTALHLACQSLVTGESTQAVVSGSSLILGPETMVT--MSMM-----RFL-SPDGRCYAFDDR---A-NGYARGEG-
VTVLLLKR--LDDALANGDT-IRAVIRGTGRNPDGKTT-----GIAMPSGLAQEALIRSVYAKTG---LD-LLD----TAYIECHGTGTQAGDTTEARAISN-VF----
GPGRQVPLAIGSVKTNIGHLEAASGLAGVLKCILMLENEIILPNRNFK----HANLNIPLEEWKLRVPTTVEPWNSMT-----------
TRRASVNSFGYGGTNVHAILESADNYMGQRADPQVFAVSAFDPTAGEACAKNLAYYVEERQDMTFLSSLAFTLSDRRT

>AAR90237.1_polyketide_synthase__partial__Botrytis_cinerea_

-----TRGGNFLKG--DLAAFDPSFFSITAEEAKAMDPQHRLLLETTYQALEN---AGIP---
LSKASGTKTGVYTGSMADDY-----RFTSLK--DPEDLPKYAVTGTA-M--
SLLANRLSWFFNLGGPSINLDSACSSSLMALDIACQGLRNGDSSMAIVAGSCLISCLETWIS--LCNM-----GFL-SPDSRCYSFDNR---A-NGYARSEG-
VGVVIIKK--LSDALKDNDT-IRAVIRATGSNSDGHTP-----GITQPSREAQTRLIEETYIKGG---LD-PAK----TRYFEAHGTGTPLGDPIEAGAIGS-VFREYRSA--
TEPLYVGALKSNIGHLEGGSGIAGLIKTVLVLEKGIIPPNANFE-QA-NSNID--AEFFHLRFPTEQAPWP--SQ---G-------
LRRASVNSFGFGGSNSHCIVNRATASFKFVSDPQLLVFSAVDKDGIRRVAEAYRSYF-AGHALSFLRDLAYTLSVRRT

>AAR90238.1_polyketide_synthase__Botrytis_cinerea_

PIAIVGISCRFPAGANSPEELWSLISQGKSA-WSDVPEDR-FNWKSFLHPSSD-VVGTINSR-GGHFIDQ---
DIRTFDAGFFGIPPAEANAMDPQHRLQLETAYEALEN---AGIS---LEKVRGSSTSVYVAIFNRDY-----DRMMFK--DTNDIAKYHLLGSG-E--
AIASNRISYTFDLKGPSMTVDTGCSGSLVALHQACQGLRSGDTDMALVGGTSLILSPDTMVP--MSRM-----NVL-DPSGKSFVFDDR---G-VGYGRGEG-
VATIVLKR--LKDALNAGDH-VRAVIRNTGINQDGKTS-----GIALPSQVAQQALANHVFKQVG---LD-PCK----INYVEAHGTGTIAGDLAETKSIAN-VF----
CTERKKTLYIGSIKSNIGHLESASGAAGLLKAILVLEKGLIPPNVNLS----NHKKGLDLEQSNIVVPSKIEKLSVDEDG----------
KSRIAINSFGYGGTNAHAILETAPLSQGSMASAKLFSLSAKSKASLIENIKKLRIWASEFKATNQLENLSYTLNQRRS

>AAR90239.1_polyketide_synthase__Botrytis_cinerea_

PVAIIGSSCRFTGA-SNTSKLWELLKEPRDV-LSKIPEER-FLAEGFYHQDGQ-HHGTSNVL-HSYLLDE---
NPLAFDSAFFHIHNREAECIDPQQRLLLENVYETIES---ACYP---METIRGSDTGVFVGLMCADY-----YDVQMR--DPETLPQYFSTGTA-R--
SIVSNRVSYFFDWKGPSLTIDTACSSSLVAVHQAVSALRNGECRSAVAAGVNLIFGPEMFNG--EANL-----KML-SPTGTCKMWDAS---A-DGYARGEG-
CGSVMLKL--LEDAIRDGDR-IESVIRETGVNSDGRTM-----GITMPSASSQEKLIKETYRRAG---LD-PSKESDRCQFFEAHGTGTLAGDRQEAEAISR-
AFDLYQTSQDNLPLSVGSIKTIVGHLEGCAGIAGLLKASLALQHSFIPPNMHFQ-EL-NPEIE--PFYQNLKVVTSLQPWP--LI---PDNT----
PRRASINSFGFGGTNAHAILESYSAEMTQINTIVPLLLSASSEHSLVELTKKYLDHL-KTHPFQNLQDICWTALSCRS

>AAR90240.1_polyketide_synthase__Botrytis_cinerea_

PIAIIGRGCRLAGGCQSPSELWNFLQTPYDV-SQEFPPDR-NSASRHSTSTRGYFLSQ--------------DVREFDASFFSLSPLEAQAMDPQHRLLLETVYEALEE---
AGIP---AETLRGSDTAVYTGVMFHDY-----LSLSSQ--DHMAIPKYHITGTA-P--
NKASNRISYFFDWHGPSVTVDTACSSSLVALDHAVQQLRSGSSTLAVAAGANLLLDGRPFIG--FNNM-----GML-SPTGSCKMWDTE---A-DGYARGEG-
ILAMLLKP--LRLALADGDN-IQCVIRETGVNHNGRTS-----GITLPSASAQTSLIRDVYHRAG---LD-PTNPSDRPQYIEAHGTGTQAGDPLEAEALAA-
AFSLTSSQPDEAHMLVGSIKTVIGHTEGAAGLAGVLKASLAIQHGIIPPNLGFQ-QL-NKKVA--PYCTNMDVVTSVQPWP--AM---ASNT----
PRRVSINSFGFGGANAHVILESVPLLSPRSDSVLPYVFSAASEQSLISMLKRYENYL-QENPSINPNELTTTLASHRS

>AAR90241.1_polyketide_synthase__Botrytis_cinerea_

PIAIIGMACRFPGGSNSPSKLWDLIKSPRNL-SKRVPDER-FNSEAFFHTNGS-YHGATDSR-EAYFLEE---
DVALFDNAFFNIQPGEAEAVDPQQRLLMETVYDSLCA---GGQT---IEGLRGSNTGIYVGMMCDDW-----AQAINR--DWESTMTYAATGQS-R--
AIVSNRLSYFFDWHGPSMTVDTACSSSLVAVHQGVTSLRNGECPVVIAAGVNLILGPGMWIA--ESKL-----HML-SPTGTSKMWDES---A-DGYARGEG-
IASVVMKR--LSDALRDGDP-IECVIRGTGVNQDGK--TP---GLTMPNGKAQADLIRDTYQRAG---LD-
IHDPRDRPQFFHAHGTGTQAGDPQESQAIDSAFFSGPDKK--LETMAVGSIKTIIGHTEGTAGLASLIGSVMAIKHGVFPPNLHFQ-NL-SPKVA--
PFYNHLHIPTTATPWP--EL---PSGV----PRRISCNSFGFGGLNAHAIIESYESEATLASVFTPLTISAASASALKATLSDIRSYL-EKSPDVNMHDLAHTLQSRRS

>AAR90242.1_polyketide_synthase__Botrytis_cinerea_

PIAVIGTGCRFPGGASSPSKLWNLLHHPYDL-TQKVPSSR-FNIKAFYHPNGE-HHGTTNAT-KSYFLNE---
DPTTFDAPFFNINPREAEALDPQQRLLLETVYEALEA---AGLS---IEEMQGTSTAVYVGLMCADY-----FDVLMR--DIEDIPQYLATGTA-R--
SIMSNRISYFFDWKGPSMTIDTACSSSLVAVHNAISTLRSGQSRTAIAAGANLIFGPEMYIG--ESNL-----HML-SPTGRSQMWDSR---A-DGYARGEG-
TAAIVLKT--LKNALEDGDD-IEYIIRETGVNSDGKSK-----GITMPLAASQADLIRQTYARAG---LD-
CTKPSERCQYFEAHGTGTPAGDPVEAEAISSAFFPQRSDILNSEPLHVGSIKTVIGHLEGAAGLAGIIKAGLALKEKTIPPNLHFQ-SL-NSAIE--
PFYGNLNVPTAPLPWP--AV---EG------PLRASVNSFGFGGTNAHAILESYEVPSLESTALIPFTISAISEDCLIQNITNFSDYI-EEHEGVNLIDLGYSLLGRSN

>AAR90244.1_polyketide_synthase__Botrytis_cinerea_

PIAIVGISCRLPGNVSTPDEFWELCSRARSG-WSEIPKER-FDTASFYHPNPG-KAGC-FNAAGGNFLKE---
DVGLFDAPFFSLTAQEATSMDPQQRILLECTFEALES---AGIP---KHEIVGKDVGVFVGGSFSEY-----ESASFV--DTDSVPMYQATGKLFF----
LSNRISHFFDLRGPSFTMDTACSSSLVALHQACQSIRNGECKSAITGGCHLNMLPENWISIPFCEIPSNKYRLF-SDEGRSFSFDSR---G-TGYGRGEG-
CGLIVLKP--LEQALKDNDN-IRAVIRGSGINQDGKTP-----GITMPNGSAQESLMKWVYESAG---ID-PKD----TGYVEAHGTGTKVGDPIEATALYN-
VFGEGRNA--RNPLFIGSVKSNIGHLEAASGIISVIKTAMMLERGFILPNHDFK----TPNENIPFSKWHLKVPISQRPWP------------
RAKKFASVNNFGFGGTNAHVVLERAPFSEKALQDRRMIALSANDKGALEALMKNIGIYLERRPEFGLMSNVAYTLGQRR-

>AAR90247.1_polyketide_synthase__Botrytis_cinerea_

PIAVVGMSCRLSGDASDTQKLWELLIKGRSA-WSKVPKDR-FNQEAFHDPSAEGKPGR-TSTDGGHFLKD---
DIKAFDASFFGINPIEATAMDPQQRLLLEIAYEAFEN---AGLT---LEQLSGSNTGVYVGQWASDY-----QEMLLR--DIDFPPIYQASGVG-A--
AISSNRISYCFNLHGPSLTLDTGCSASLVALHQAVHSLRAGETDKCFVAGVNLQLDPQRYGY--QNKL-----SMF-SKQGKSFTFDHRAKTA-SGYGRGEG-
CSGVVLMP--LSQAQKQGFP-IRAVVQNSVANQDGKTN-----GITVPSAEAQSAAIEKAYSQVG---LV-PYA-----DYVEAHGTGTAVGDPIEARAIAKVLG------
ESDSPLPIGSLKANIGHTESAAGLTGLIKAVLMLEHNMIPPQVNFE-KA-NPEID--LDALNLRIPITLESRP--------------
LKRISVNSFGYGGTNAHVVVDAAREEDASRAKERLFVLSAASEKSCQDMSSKVAKYLESKADSTLLSRLAYTLSRRSV

>AAR90260.1_polyketide_synthase__Bipolaris_maydis_

PLAIVGLATRFPQEAYNTEQLWQFLLAKRCA-HTPIPEGR--MGPGHYHPDPE--HGGTHAVAGGHFLAE---
DPAYFDSSFFGITKGEAMSLDPQQRVVLENVYLALEN---SGFT---LEQVAGSNTSVYVSGFNHDH-----LAILNS--DPETTLRHRVTGLT-N--
SMHSNRVSWFFDLKGPSVTIDTACSSSMAALHLGSQSLRTGESDMSIITGVTILNYPGDVNG--MSHQ-----GFL-SPDGRCFSFDHR---A-NGFARGEG-
AGTVIVKR--LSDALRNGDT-IRAVVRGTGINQDGRTP-----GLTLPSSDAQERLIRTTYASAG---LN-FDD----TTLVEAHGTGTKQGDAIEARGIAQ-AF------
RKDKPLYLGSVKASVGHLEGAAGVAGIIKSVLALENGVIPPQANFE-KA-NPKIP--FQKWNLSIADKAVPWP-----------
TQGLRRVSINSFGVGGTNAHAILDDAYHRDHELTGNHRTAIEAPSMEKIDSLSRAAEALI-APSEQSETQHLNGDDSAPKQ

>AAR90261.1_polyketide_synthase__partial__Bipolaris_maydis_

PIAIIGIAFRGPGDARDPEAFYRMLIEGRSA-RTEIPKDR-YNVDAFYHPDPE-RLGSIQQR-YAHFLQQ---
DFKAFDAPFFSITPKEAKAMDPTHRILLEAAYEGFEN---AGLT---LDQVSGTQTSCYIGTFTADF-----PNLQAR--DNEGPSIYHATGMS-S--
SLASNRLSWFYNLRGPSLTIDTACSSSLTAFHLACQSLRTGESEMSVVGGANLMFGPDMSIL--LGAA-----KIL-SPEGKSKMWDAN---A-DGFARGEG-
FGVTILKP--LDTALRDGDT-IRAVVLATATNEDGHTP-----GISLPNSEAQQDLIRRAYQMAG---VD-PAE----TGYVEAHGTGTMAGDPLEAKAILKTVG------
GRKSSLYVGSVKTNIGHLEGAAGVAGVIKAALAVERGLIPQNLWFE-KL-NPEIN---LPENVEIPLKLTPWPSDG-----------
PRRASINSFGFGGANAHVILEDTASLHDLQPTPKLFTISANDQEGVRRNVERLQRYLSAKTSAAFLSDLAFTLSAKRS

>AAR90262.1_polyketide_synthase__Bipolaris_maydis_

PIAIVGMSFRGPGSANNIENLFKMICEKRES-RTAIPKSR-WNNKAFYHPNFQ-RHGSHNVE-YGHFFHD---
DISKFDAPFFNMTKEEAAALDPAQRLLLESTYEALEN---GGIP---LEKIVGTKTSVFVGSFATDY-----TDLLTR--DPESVPMYQCTNSG-
QSRAMISNRLSYFFDLHGPSVTVDTACSGSLVALHLACQSLRAGEAKSAIAAGVNVVLNHEFMTT--MSMM-----KFL-SPDGRCYAFDER---G-NGYGRGEG-
VGTVFLKP--LADALKDGDP-IRAVIRGSASNQDGKTS-----GITLPNPVAQEALIKDVYHVAD---LN-PID----TSIVEAHGTGTAAGDPLELRALTNSFC----
GAPEDRPLIIGSIKSNLGHLEGASGIAAVVKAVLMLEQEVILPNQNFE-KP-NPRIP--FVDWKLRVATNVQPWD--SS---G-------
PLRVSINSFGYGGSNAHVILESTRTPVPNYSHPKLFTLSVFDEPTAQQQIQTLVKHL-HERGIGFLDDLAFTLNERRT

>AAR90263.1_polyketide_synthase__Bipolaris_maydis_

PIAIIGMNMKFPGDAVSAQSFWKLIVSARNV-SKEVPADR-FKI-AFYHPDPN-RLDSIRIR-NAHFMTE---
DPRAFDAPFFNMSHAEASVLDPQQRGLLEGAYRTFEN---AGIP---MESLSRTPTSVYCASFSRDG-----ETITGR--DFASQSRYHATANG-S--
SMLSNRISHFFDLAGPSLTVDTACSSGLYALHLGVQSILTGESTMSLVCGANTFITPESQAL-ALSNG-----GFL-SVDGKSYSFDAK---A-NGYARGEG-
FGFVLLKP--LDAAIRDGDV-VRAVVRATGANQDGRTPS-----ITQPSQQAQLDLLRDTYRVAG---LN-VAD----TGYVEAHGTGTPVGDPIEASAIGQ-AF--
RDGRSLDRPLYIGSVKASIGHLEGASGLAGVIKAVLSLEKAVIPPIAMFE-KV-NPAID--AADLQLAFPKEAIPWP--AS---E-------
VRRASVSSFGYGGSNAHVILDDA--AVPSTTPYAILPFSAADSDGTKRQAEALQTFL-ASHETTFLSDLSYTLSSHRS

>AAR90264.1_polyketide_synthase__Bipolaris_maydis_

PIAVVGFGFKFPQDVTNAETLWKLLIERRST-MTEIPKNR-WNIDGFYKENGH-RPGTVKNR-GGHFLSD---
DPARFDAPFFSIQPAEAECMDPQQRLLLETSYHALEN---AGIT---MQDAVGTRTSVHVGCLLQEY-----SQISQR--DAQMPGDYRIVGSSGL--
AMLSNRLSWFYDFSGPSMTVDTACSGGLVALHLACQELLAGSVNMSLVCGTNLCLLPDSTAL--LSSL-----NMM-SKDSVCYSFDER---A-SGYARGEG-
FGVLVLKR--LSEAIADGNN-IRGVIRSTGCGQDGNTPS-----ITSPSQSAQERLIRETYARAG---LS-LDE----TRYFEAHGTGTKAGDPCEAAAINS-VF------
TPEDPIFVGALKSNMGHPEGASGIAGVIKTLLVLEKGIIPPNVYPE-RI-NPAVT--AAGPNLRFPLEPVTWP-----------
TSGVRRASVNSFGYGGTNAHVVIDDALS-------------------------FLREQG--------------------

>AAR90265.1_polyketide_synthase__Bipolaris_maydis_

PIAVIGLDAKLPCDGDSVQQFFDFLIAGRSA-RKPVPSDR-YNADAFWHPDHH-RDGIIASK--QGHFMNS--
SVKAFDAPFFGITPAEAAALDPQQRLLLESSYAALEN---AGYT---LQEILGSNTGVYTGSFVYDY--RD-VMIK----DTDVALTYSGTGTV-P--
STLAGRVAWFFDFRGPAFTVDTACSSSMVALHQAVIGLKSRECNLALACGTNVILSPEFGQQ--LNGL-----GVL-SPQGASKSFDKE---A-NGYGRGEG-
ISVVVLKR--MSDAIRDGDT-IRAVIRNSGIGHDGK--GA---PLPAPVRESQVNLVRRCYAQAK---ID-PSE----TRLFEAHGTGTNAGDPTEAGAISD-IF----
SRSEEEPIYIGALKSNIGHTEGNSGVASFIKAVLTLESGIIPANAHFK-EV-NHAIP---KKWHFKFPTVATPYP--KT---PSG-----
VRRVSINSFGISGTNAHVVLEDARHINGTAFAPQLFVLSAFDQDGVSRLCKAYAEYL--PKMTS-LYNLSYTLARKRS

>AAR90267.1_polyketide_synthase__Bipolaris_maydis_

PIAICGMALRLPGALKTPAQFWTFLTEKKDA-RAPIPFAR-FDAEAFYSASG--KSGYFNSK--
NGYYLDDSAEVGALDTSLFRMSQQESERMDPQAKILLELARECFES---AGET-----DWRGKDIGTYVGSFGNDW--LE-MAAK----DRLDKNMYKVSGYS-D--
FLLSNRLSYEYDLRGPSMTIRTACSASLMGLHEACLAIRNGDCSAALVGGSNLFWSPDTMAD--MSEQ-----GVT-SPNASCRSFDAD---A-DGYARAEA-
VNLIYIKP--LSAAIKHGNP-IRAIIRGSSLNADGK--TP---GPSMPSSSSQAALIRRAYQNAG---IP-ESE-IIRTAFVECHATGTATGDPIEATAVGN-VF----G---
NGGVYIGSVKPNVGHSEGASGITSIIKAVLALENRVIPPNIKFE-TP-NPKIP--FLEKDLKVPTDCLGWP--------KDK----
LERVSVNNFGIGGANAHFILDSAASFLESLKWGKLFLITASTSESLEMQTRNLRDYA--LSN-TKAEHGSYTLACRRE

>AAR90269.1_polyketide_synthase__Bipolaris_maydis_

PIAIVGMGMRLPGNCHNSEAFWDLLINQRDG-MIDIPSSR-WNSEGFYDPHG--RPGTTKVN-KGNFLGSI--
DPADFDGSFFSMSAAQIAKVDPQHRVLLETTYEALEN---AG-----ESNFRGKKVGVYVGMFADDY-----VEMQSK--DSEPHEFLSLTGHL-D--
LFGSNRVSHEFDWTGPSMTIKTGCSASLVCLDQAVKALRAGDCDAAIVGGANLIMSPALSCA--LVAQ-----GVN-SADGICRSFDAK---A-TGYGRGEA-
VSSLLVKR--LDDALRDGNP-IRAIIRSTACNDDGKTP-----GITQPNTVAHEALIRAAYRTAGISEED-LNK----TGFFECHGTGTAVGDPIEVGAVAR-VF-------
GKDGMIIGSVKSNIGHSEGASGNTSVIKAILSMEHQIIPPNVNFE-TP-NPKIP--WKEANLRVPTECMKWP--SD---R-------
LERASVNSFGIGGTNAHVILESAAASTEQKPRLSLLLFTARSEISLQESINKHADYI-QKHGSSRLGDIAYTLGTRRE

>AAR90270.1_polyketide_synthase__partial__Bipolaris_maydis_

PIAIVGSACRFAGGANSPSKLWDLLRNPKDI-RSNITDKR-FNANGFYHPDGS-HHGHMNVL-QSYLLEE---
DTRLFDAEFFGINPVEAKAMDPQQRLLLEVVYESIES---AGLS---IDRLRGSDTAVFAGLMCGDY-----EARMLR--DLDQVPTHFATGTS-R--
AVMSNRVSYFFDWHGPSVTIDTACSSSLVAVHHAVQALRSGDSHAAVACGSNLIFGPEMYVI--ESKL-----KML-SPDSLSRMWDKD---A-NGYARGEG-
VTAVVLKT--LSQALADNNR-IEVVIRETGMNSDGA--TP---GITMPSASAQRDLIRSVYRKAG---LD-PESAEDRPQYIEAHGTGTPAGDPIEAEALSTAFF----
GEKMTTPIYTGSIKTVLGHTEGSAGLAALLKVTQAMQNSTVPPNLWFQ-QI-NPKLK--PFYGNLQIPTHALIWP--KP---FGRR----
PKRASINNFGFGGTNAHAIVESYELASSAVTVSTPFVFSAASTESLRSNLAAYATYL-DANPQTNTRDLAYTLRERRS

>AAS57290.1_polyketide_synthase__partial__Fusarium_graminearum_

----IGVGCRLPGGANNLDNLWKLLSESRNG-QTEIPKDR-WNADAWFDAYPD-AKQSMVTK--YGYFLQD--
DISQFDAKFFGISSAEANSMDPQQRLFLMTTYEALED---AGIP---VETLRGSNTGVYASIFERSY--DR-MGHK----DLSTIGRTHLNGTG-E--
SDLSNRVSYCFDLRGPCMTIDTGCSGSLVGLHQACQSLRLGESELALVGGSQLVIQPDVLCI--MSGM-----GML-NPDGKSYAFDSR---G-AGYGRGEG-
VATIVLKR--LDNAIKDGDR-IHAIIANSGMNQDGK--TP---GLNTPSGEAQAALSRRVYQEAG---LN-PAD----TSFVEAHGTGTQAGDREEIGSISK-VF----
CEGRTDDLYIGSVKTNIGHLEATSGIAGLLKSILILKHGQIPATLNFI-KP-KPSLK--LYEKKIKIPSELTKLP--TP---QNG-----PPRVSLNSFGYGG--------------------
----

>AAS57294.1_polyketide_synthase__partial__Fusarium_graminearum_

----IGTFCRFHGGANTPSKLWDLLIEKRDV-QSSIPPVR-FNVDAFIAVM-D-KNGCTDVK-KAYLWAE---
DTRLFDASFFKINPREAEAMDPQQRLLLEAVYEATET---SGLP---MEDLKGSDTAVYVGCMTGYY-----HEMLMR--EPQDMPKYMATGTA-R--
SILSNRISYLFDWKGPSMTIDTACSSSLVAVYDAVTALRNGVSRIACAGGVNLILGPEMMIS--ESKL-----HML-SPTGRSRMWDAS---A-NGYARGEG-
VAAIMMKT--LSQALADGDH-IEGIIREIGVNSDGRTN-----GITLPSPDAQKVLIRHTYRNAG---LDVFKD---RCQFFEAHGTGTPAGDPLEGRAIHKAFF--
DGSDIVNEPMYVGSVKTAIGHLEGCAGFAGMIKVIEAVKTGIIPPNQLFE-TL-NPAVK--PYTSNLKLPVESQPWP--KL---TEGF----
PRRASVNSFGFGGTNVHAIIEQFDNSSVNEVICTPLVLSANCELS--------------------------------

>AAT28740.1_FUSS__Fusarium_verticillioides_

PIAIIGTSCRFPGGANTPSKLWDLLCEKRDV-QSRIPNDR-FNVDAFYSTNGD-KNGCTDVK-RAYLLSE---
DIRVFDASFFKINPREAEAMDPQQRLLLEAVYEATEA---AGLP---MEDLKGSDTAVYVGCMTGDY-----HEMLMR--DPQDMPKYMATGTA-R--
SILSNRISYLFDWKGPSMTIDTACSSSLVAVYDAVTALRNGVSKIACAGGANLILGPEMMIS--ESKL-----HML-SPTGRSRMWDAS---A-NGYARGEG-
VAAIMMKT--LSQALADGDH-IQGVIREIGVNSDGRTN-----GITLPSPEAQKFLIRQTYKKAG---LDVFKD---RCQFFEAHGTGTPAGDPLEARAIHEAFF--
TDGDIVSEPMYVGSVKTAIGHLEGCAGLAGLIKALEAVKRGVIPPNQLFE-NL-NPALK--PYVSNLRLPTESKPWP--KL---APGS----
PRRASVNSFGFGGTNVHAIIEQFDHSSADGIISTPLVLSANSDLSLRKQIAHFAEAI-EHNDKGEIDRIIFTLAQRRS

>AFL91703.1_ArmB__Armillaria_mellea_

PIAICGMSVNMPGA-QSVAKLWEVLEKGINT-VSEVPEHR-FKVSDYNDPKKKSRTMA---AHTGNFID----
EPDAFDNKFFNISPREARSMDPQQRVLLHTAYEALED---AGYV---PNSTPTNTFGCYVGVATNDYVQN----------LRNDIDVYYSTGTL-R--
AFLSGRISYALQFSGPSIVVDTACSSSLIAVYQACRALMNRDCNAAVAGGVNVIGSPDMFLG--LDRG-----HFL-SPTGQCKAFDAS---A-DGYSRSEG-
CGIFVLKR--LSDAVAENDQ-ILGVIRGVEVNQSGNAY-----SITRPHAPTQENLFTQTLERSG---LD-ASR----ISVVEAHGTGTQAGDPIELESIRG-IFAKNRKT--
NNPLHITSVKANIGHLEAASGAAALAKLLLMLRHRTIPRLISLK-NL-NPRIK-PLASDNVIIDTKQVAWAVPDE---------
SLPRVALLNNFGAAGSNGALLLEEYIPEKIEVSSTFIVGLSAKNEQALVDLRASYIEYLRSPASAVSLADIAYTATARRR

>AGI05041.1_fumagillin_dodecapentaenoate_synthase__Aspergillus_fumigatus_Af293_

PIAIVSAACRLPGHVNGPHKLWELLQSGGTAVSNEVPQSR-FSSEG-HFDGSG-RPGTMKAL--SGMFIEDI-
DPAAFDAAFFNLTRADAIAMDPQQRQLLEVVYECFEN---GGIP---IEKVRGKQIGCYVGSLNGDY--HD-MQMR----DPEQRVSGHAVGTG-R--
AILSNRISHFFDLRGSSFTIDTACSSGLVGVDVACKNLRAGTLTGAVVAGVNLWLSPEHTEE--RGTM-----RAAYSASGKCHTFDAK---A-DGYCRAEA-
VNAVYLKR--LSDAVRDGDP-IRAVIRGTASNSDGW--TP---GINSPSAQAQAAMIREAYANAGIDSSE-YAE----TGYLECHGTGTPAGDPTEVKGAAS-VL----
AHPPASPLIIGSVKSNIGHSEPGAGLSGLIKAMLVVEEGEIPGNPTFL-NP-NPAID--FDNLRVYATRIRIPWP--KE---SSH-----
YRRASVNSFGFGGSNAHAVLDNAEH--TVTRRPQVLVFSANDMDSLKRQISTLSAHL--LNPRVKLSDLSYTLSERRS

>AIA58899.1_HRPKS__Penicillium_brefeldianum_

PIAVVGIACRLPGHSSTPKKLWDFLERGGIA-ANDTPSTR-FNLAA-HHDGSK-KPKTMRTP--GGMFIEDA-
DPRDFDAGFFGISGADAAAMDPQQRQLMEVVYECLEN---SGVP---FEKLYGAQVACHVGSYAVDY--DA-IQAR----DPEDRAPGAVVGIG-R--
AMLSNRISHFFNFKGPSMTIDTACSGSLVGLDVACRYLHTGEVDGAIIGGANMYFSPEHNLN--TGAM-----SVANSLSGRCHTFDVK---A-DGYCKAEA-
INCVYLKR--LSDAVRDGDP-IRAVIRGSATNSDGN--TP---GIASPNSAAQAAAIRSAYANAGI--TN-LND----TSYLEFHGTGTQAGDPLEAGGVAS-VF----
SAKPEAPLYIGSVKSNIGHSEPAAGISGLIKAILSIEKDLIPGNPTFI-TP-TPKID--FEGLKLQPSRANRRWP-------AAP-----
FKRASVNSFGYGGSNAHVIVEEPKV--VAGGRLQLLVLSANDEASLRANATTLKNYL--TNPNISLGDLSHTLSERRS

>AJG44381.1_MpaC__Penicillium_brevicompactum_

PIAITGMACRYPNA-DTLAQLWELLELGRCT-VKSPPESRFHMSDLQREPKGP--------F-WGHFLE----
RPDVFDHRFFNISAREAESMDPQQRVALQVAYEAMES---AGYLGWQPNRLSQ-DIGCYVGVGSEDYTENV---------ASRNANAFSITGTL-Q--
SFIAGRISHHFGWSGPSISLDTACSSAAVAIHLACKALQTNDCKIALAGGVNVLTNPRVYQN--LSAA-----SFL-SPSGACKPFDAS---A-DGYCRGEG-
AGLFVLRP--LQDAIDNGDP-ILGVIAGSAVNQGSNNS-----PITVPDAEAQRSLYNKAMSLAG---VS-PDE----VTYVEAHGTGTQVGDPIELDSLRRTFG----
GPHRRNNLHIGSIKGNIGHTETSSGAAGLLKTILMLQQQRIPRQANFN-QL-NPKVK-SLTPDRLVIASESTEWV--ST-----------
KRVAMVSNYGASGSNAALIVKEHAPAPEYIQNKLAYNLAMKQNRDLPLNL---------------------------

>BAC20564.1_polyketide_synthase__Penicillium_citrinum_

PIVVVGSGCRFPGGVNTPSKLWELLKEPRDV-QTKIPKER-FDVDTFYSPDGT-HPGRTNAP-FAYLLQE---
DLRGFDASFFNIQAGEAETIDPQQRLLLETVYEAVSN---AGLR---IQGLQGSSTAVYVGMMTHDY-----ETIVTR--ELDSIPTYSATGVA-V--
SVASNRVSYFFDWHGPSMTIDTACSSSLAAVHLAVQQLRTGESTMAVAAGANLILGPMTFVM--ESKL-----NML-SPNGRSRMWDAA---A-DGYARGEG-
VCSIVLKT--LSQALRDGDS-IECVIRETGINQDGRTT-----GITMPNHSAQEALIRATYAKAG---LD-
ITNPQERCQFFEAHGTGTPAGDPQEAEAIATAFFGHKDGTGEKDELFVGSIKTVLGHTEGTAGIAGLMKASFAVRNGVIPPNLLFE-KI-SPRVA--
PFYTHLKIATEATEWPIVAP---------GQPRRVSVNSFGFGGTNAHAIIEEYMATSDADACSLPLVLSSKSQRSMKATLENMLQFLETHDD-
VDMHDIAYTLLEKRS

>BAC20566.1_polyketide_synthase__Penicillium_citrinum_

PIAIVGMGCRFAGDATSPQKLWEMVERGGSA-WSKVPSSR-FNVRGVYHPNGE-RVGSTHVK-GGHFIDE---
DPALFDAAFFNMTTEVASCMDPQYRLMLEVVYESLES---AGIT---IDGMAGSNTSVFGGVMYHDY-----QDSLNR--DPETVPRYFITGNS-G--
TMLSNRISHFYDLRGPSVTVDTACSTTLTALHLACQSLRTGESDTAIVIGANLLLNPDVFVT--MSNL-----GFL-SPDGISYSFDPR---A-NGYGRGEG-
IAALVIKA--LPNALRDQDP-IRAVIRETALNQDGKTP-----AITAPSDVAQKSLIQECYDKAG---LD-MSL----TSYVEAHGTGTPTGDPLEISAISA-AF-------
KGHPLHLGSVKANIGHTEAASGLASIIKVALALEKGLIPPNARFL----QKNSKLMLDQKNIKIPMSAQDWPVKDG-----------
TRRASVNNFGFGGSNAHVILESYDRVEDGSKQSRIYVVRAKDEQACRRTIASLRDYIKSVADIPFLASLAYTLGSRRS

>BAD83684.1_PKSN_polyketide_synthase_for_alternapyrone_biosynthesis__Alternaria_solani_

PVAIIGIAFRGPGDARDPEAFYRMLVEGRNA-RTEIPKDR-YNVDAFYHPDSE-RLGSIQQR-HAHFLQQ---
DFKVFDAPFFSITPKEAKAMDPTHRILLEATYEGFEN---AGLS---LEKVSGTQTSCYIGTFTADF-----PNLQAR--DNEGPSIYHATGMS-A--
SLASNRLSWFYNLRGPSLTVDTACSSSLTAFHLACQSIRTGEAEMSVVGGANLMFGPDMSIL--LGAA-----KIL-SPEGKSKMWDAN---A-NGFARGEG-
FGVTILKP--LDAALRDGDN-IRAVVLATAANEDGHTP-----GISLPNSEAQQELIRRAYQIAG---VD-PAE----TAYVEAHGTGTQAGDPLEARAILK-
TIGSVEGR--KSDLYVGSVKTNIGHLEGAAGVAGIIKAALTVERGMIPPNLWFE-KL-NPEID---LPDNVKIASKLTPWP------------
HSGPRRASINSFGFGGANAHAILEDAASLRDVYPIPKLFVLSSHDQEGVKRSAERLQEYLASKTSTSFLNDLAYTLSAKRT

>BAJ09789.1_polyketide_synthase__Alternaria_solani_

PIAIVGMGCRWPGGVRDASSLWELLKNKRSG-YREFGDHR-FSRKGFHHPNSE-HPGT-VAT-EGGFLLAE--
DPRLFDHAFFGIGSLEVETMDPSQRKLLEVVYEAFEN---SGEP---WDSFSGSTTGVFVGNFSSDH-----LIIQGR--DTDHPRPYASVGTG-T--
SILSNRVNYIFNLRGPSVTIDTACSSSMYALHLAISAIRNGDCDSAIVAASNTIIDPSTXLM--MTKL-----GVL-SPTSTSHTFDSS---A-DGYARGEG-FSALYLKR-
-MSTAVDGDYP-IRALVRGSALNANGR--TG---GITHPGREGQEAVIRKAYENAGN--LP-MKD----TTFFECHGTGTPVGDPIEISAIGN-VF----
GSTPEKPLLVGSIKTNIGHTEPASAIAGIMKVVLALENGFIPPSIGIK-KL-NPKLD--LKGGRINILTENTPWP--DG---R-------
VRRASVNSFGYGGANGHCIIDDVRTYKKRTANTSIGHINGHTNGHTNGHTNGHTNGH-TNGHTNGHTNGAH-------

>BAQ21221.1_polyketide_synthase__Oryzias_latipes_

GIAVVGMGCNFPGG-EGLENFWKVLLEGQNC-VVDIPSER-FDTSFWSDADAT-KPGKTQTT-RAALID----
GFNEFDHKFFGISEAEADVMDPQQKLLLQCSYRALED---AGTS---IESISGSRTGVYVGLMNRDY--EM-LRNN----NPNTISHYNATGTA-T--
SVAANRISFTFNLTGPSFAIDSACSSSLVALHVACQAMRLGDCEMALCGGVNCIIEPRVFVA--LSKA-----KMT-SPEGTSKPFSIR---A-DGYGRGEG-
CGVLLLKP--LRSAKRDRNK-IWGIIRKTAINQDGRSVTP----ITKPSKVQQEELLRGIYSD--------IAD----VQYIEAHGTGTPAGDTTEAGSISD-AI----
AEPGSKTLMVGSVKGNIGHTESAAGVAGVIKVLLMMKHETIVPSVFYS-DD-SAGVD--MRALKLRVPIKAEKWE--AA---G-----
PHGRVAGVNSFGFGGTNSHVVLREYTNATWL----KVCVLSAASEKSLILTINDTHRRL----NQVDLRGLSYTSACGRS

>CAB04244.2_Fatty_Acid_SyNthase__Caenorhabditis_elegans_

DIVISGVSGRFPRC-DNVKMFGDMLLAGEDL---VTEDSLRWTPGFCDLPK---RHGKLK-------------
VLNKFDAGFFQVTPKQANFMDPQVRLLLEASWEAMVD---AGIN---PTDLRGSKTGVFVGCSASET-----SGMLTQ--DPDTVTGYTLTGCV-R--
SMFSNRISYTFDLQGPSFSVDTACSSSLLALQLAVDSIRQGQCDAAIVAGAHLTLTPTAALQ--FLRL-----GML-TDKGSCRSFDES---G-DGYCRTEG-
VAAIFIQR--KKKAQR-----LYATVVHAKSNTDGHKEHG----ITFPSGERQAQLLQEVYSEAG---ID-PNS----VYYVEAHGTGTKVGDPQEANAICE-VF----
CSKRTDSLLIGSVKSNMGHAEPASGVCSLTKILLSIERQLIPPNLHYN-TP-NQYIP-GLTDGRLKVVTEPTALP---------------
GGLIGINSFGFGGSNTHVILKAADHPITPHPFTKLVTYCGRTQEAVENIFTEIESNK-DD---LYLQALLANQANMPA

>CAC44633.1_polyketide_synthase__partial__Fusarium_fujikuroi_

PIAIIGSACRFPGDSSSPSKLWDLLKSPRDL-LTKVPPNR-YNADAFYHADSK-HHGTTNVR-HSYFLNE---
DPARFDNNFFNIQPGEAEAIDPQQRLLMEVVYQGLCA---SGQT---IEGLRGSPTAVYVGVMCDDW-----SGIITR--DLEVFPQYGATGMA-R--
SIMSNRISYFFDWHGPSMTIDTACSSSLVAVHQAIQTLRSGESEVAIAAGANLILTPGMYIA--ESKL-----SML-SPSGRSKMWDQD---V-NGYARGEG-
IAAVVLKP--LSAAIRDNDH-IDCIIRATGVNQDGR--TP---GLTMPSATAQADLIRSTYARAG---LD-INKPEDRPQFFHAHGTGTPAGDPREAEASTVRSI----
PDVKDDKLYVGSIKTVLGHTEGTAGLASLIGTALAIQNKTIPPNMHFD-VL-NPKIK--PFYDNLEVPTKAIAWP--ET---HKGQ----
PRRASINSFGFGGTNAHAIIEAYEPDSAPGPLFSPLTFSASSEPSLRSLLSSYSDHL-KSNPDLSLKDLAYTLQTRRS

>CAG28797.1_polyketide_synthase/peptide_synthetase__Magnaporthe_grisea_

PIAIIGSGCKFPGGSTTPSKLWELLKDPKDI-VSEIRPDR-FDVDKYFHPDHK-HHGTSNVR-HSYFLEE---
NFKHFDAKFFGIRPQEAMAMDPQQRFLLETVYESLEA---AGIT---ISDLKGSQAGVFVGNMGVDY-----SELLSQ--DIDAFPTYFAPGTA-R--
SILSNRISYFFDLHGPSVTVDTACSSSLVAVHQAVQSLRLGETPVAIVCGANLLLGPAQYIA--ESKL-----QML-SPNGRSRMWDAS---A-DGYARGEG-
FASIVLKP--LSVALANGDH-IECIIRETGCNQDGRTK-----GITMPSPLAQCKLIQETYKRAG---LD-
LSKSSDRPQYFEAHGTGTPAGDPVEAEAISTAFFGPESGFSHDPKLYVGSVKTVIGHTEGTAGLAGLIKASLAMKAKSIPPNLHLE-RV-NPAVQ--

PFYGNLEIPTRLMDWP--EP---APGQ----PLRASVNSFGFGGANAHVILESYTPTAAAGPVFSPFVFSASSDKALASMLSAYSDYL-
SLNPTVDLRSVAYTLSQHRS

>CAG29113.1_TPA__putative_polyketide_synthase__Magnaporthe_oryzae_7015_

PIAVVGTGCRFPGGANSPSKLWEILEKPRGL-LKKVPAER-FDVDSFYHPDGS-FHGRTNAA-NAYFLDQ---
NIRAFDANFFNIQHHEAEVTDPQHRLLLETVYEALAT---AGLR---VEDLRGSSTAVYCGQMMNDY-----KDLVNF--DQDRLPTYTATGTA-A--
SILSNRVSYFFDWHGPSMTIDTACSSSLVAVHHAVQQLRSGSSKVAIAAGANLILGPVPLIV--ESKM-----NML-SPTGRSSMWDEK---A-NGYAR-EG-
VAAVVLKT--LSQALSDGDN-IECVIRETGVNQDGR--TP---GITMPSHQAQEALIRETYAAAG---LD-
LSRAEDRCQYFEAHGTGTPAGDPQEAEAISRSFFGHGERPPREEPLYVGSIKTVIGHTEGTAGVAGLIKASLAMQNRTIPPNMHFH-KL-SPKVA--
PFYQNLHVPTEAQPWP--EL---APGV----PMRASVNSFGFGGTNAHAILEAYPNQRSSAVPIWPIPISANSELSLKATMQDLLQYL-
KSASGVRVQDLAWTLTNRRS

>CBX87032.1_BcBOA9_protein__Botrytis_cinerea_B05.10_

PVAIIGMGCRFPGDSDTPDEFWKMLAEERSG-LSRPPLSR-WNIDGFHANKA--RPGSLTPE-GGYFINE---
DIWKFDPAFFGIVQEEAKAMDPQQRKLLECVYESFES---GGIT---LSQLSGSNTGCYIGNFTSDY-----YLQGHR--DHNNPKPYSLLGSG-Y--
TIISNRVSYLFDLCGP-------------------RALQTKEIDAAVVGGTNLMLAVETQMS--TDKV-----GVL-SATSTCHTFDES---A-DGYGRAEG-VGAIFLKR--
LSDAIRDNDP-IRGVIRGTATNANGKTS-----GITQPSAKGHETVMRTAYEFAG---LD-PRD----TSYFETHGTGTQVGDPIEIKGVGNFFF----
NGTDRQKLLVGSVKTNVGHSEAASALASIIKVCLAMEKRTIPATIGIK-KL-NPKID--FKGGRIEVVQKMTPWP--KG---FS------
VCRASINSFGYGGANATAIVEAADSVRAVSRSEFLLLFSAHDISTLKSNIERCRDVA-ED---YNILDLAYTLGCRRS

>EAA49993.1_hypothetical_protein_MG03752.4__Magnaporthe_grisea_7015_

PVAIVGMACRLPGSVSDPEAFYELCCLGRSG-WSEIPSHR-FSKEGYHHPNPD-RLGCFNPV-GGCFLSE---
DIGLFDAPFFKITEREAVAMDPQHRLILECTYEALEN---AGIP---MHSIAGRNVGCFAGGSFTDY-----ELNNLR--DLETQPMHQSTGNA-P--
TMMANRVSYFFDLRGPSHTVETACSSSLTALHLAMQSLRCGDSSLVVLASSHLNVMPDHFVA--MSSN-----GLL-SGDGRSYAFDSR---A-NGFGRGEG-
AGVVILKP--LADALRDGDN-IRALVVGSGVNQDGRTN-----GITMPNGESQLDLMRKVYADNG---LD-PRD----CGYVEAHGTGTKVGDPLEMKALHD-
MFWEDR--SPRQPLLVGSVKTNVGHLEGASGIVSLIKSAMMLERGYVLPNHDFR-EG-NKEIP--FDEWGVKVPSKVMPWP--RG----------
KKYISINSFGFGGGNAHAVLAAPPR----------FRLPGEEGLSLEE-----------------------------

>EAA58609.1_hypothetical_protein_AN6791.2__Aspergillus_nidulans_FGSC_A4_

PIAIVGLSCKFAGEASTPDRLWEMLAAGRSA-WSEIPPSR-FNLKGAYHPSAD-RTNTVHVR--GGHFLEQ--
DLGLFDAQFFSFSAETAASMDPQIRLQLESVYEALEN---AGIT---LPDVAGSNTAVYAAVFSRDY--RD-GIIR----DEDRLPRFLPTGTG-D--
AMFSNRVSHFFDLRGPSITLDTGCSGGLVALHEGVKSLRTGESDMALISGEGLTDEGRFF-----------------SPDGKSYAFDSR---A-NGYGRGEG-IATIVIKR--
LSDAIIAGDP-IRAIVRESGLNQDGK--TE---TITTPSEEAQVALMRDCYRRAG---LD-YAD----TQYLEAHGTGTSTGDPIECRAIAT-VF----
KDSSEQPLRIGSVKTNVGHTEAASGLASLIKVVMALEKGKIPPSINFE-KP-NPKIA--LDEWNLRVVTTLEDWP--AG---PGG-----
VRRASINNFGFGGTNAHLIVESQAAASATNLDSQIFVFSARDKQACVNMVNNLKKYL--RQNAL-LQRVAYTLGQR--

>EAA59818.1_hypothetical_protein_AN3610.2__Aspergillus_nidulans_FGSC_A4_

PIAIVGLGCRFPGGADTPEKLWQLAYEGRQC-WEEVPSNR-YNWHAFHHPDPE-ARGTHNAR-GGFFLQQ---
DPAVFDANFFAISAAEAAAIDPQQRLLLEVSYEALEN---AGMP---LEAIRGTQTGVYVALVSRDY-----DRMIYK--DPSQIPKYHLTGCG-D--
ATACGRISYTFDLKGPCVSMDTGCSGSMVALHLACQALRLGETNAAIVAGTNLLLGPDMTIA--MSAL-----HMI-NENGRCYPFDSR---G-AGYGRAEG-
VAALVLKR--QADAVRDGDP-IRAVIRNTGINQDGKTN-----GIMLPSFEAQQQLTSTLYRQAG---LD-PNY----VCYIEAHGTGTQAGDAAEVTSIKQ-
VFASAART-RERPLFLGSIKANLGHSESTSGLAGVIKTVIALENATIPPLAALE-TL-KPDLHSLLESTKIIIPSKPVSWP--HE---G-------
TRLASVNSFGFGGTNAHVILESAPSEPNEDGRPQLFIVSAKSRVSLEAAIYNVRDWV-SRHGAIKRQELVKTLCHRRS

>EAA61508.1_hypothetical_protein_AN9217.2__Aspergillus_nidulans_FGSC_A4_

PIAIVGMAARFSGEATNPSKLWDMMVQGRTG-HSAVPENR-FDAEAWHHPSHE-RRGTIQPR-SGFFLRE---
DPAVFDAPFFSMTAKEAAGMDPMQRKLLEISYEAFEN---AGIP---ITKLPGTATGVYSGVMTNDY-----ELMTAG--DPMQLPQNAASGTS-R--
AMLANRISWFYDLRGPSFALDTACSSSLYALHLACQSLQAGETDQALVTGVNLILAPNFISQ--LSSM-----HML-SPDGKSHSFDSR---A-NGYARGEA-
LAAVVVKP--LYQALADGDT-IRAVIRGSGANQDGKTV-----GITIPNPQAQAELIRKTYATAG---LG-LEQ----TGYFEAHGTGTPVGDPIELSAIGT-SF----
GESQNCPLFVGSVKTNVGHTEGAAGLAGVVKTVLALEAGIIPPLADFQ-EL-NEKLR--LEEWKLALPLKATPWP--MP---G-------
LRRASVNSFGFGGANAHVILDDAYHDSGEGQSSKLLLFSAYDGAGIKRTEASWNSHL-ADILAMGMNDLAYTLSDRRT

>EAA63960.1_hypothetical_protein_AN1784.2__Aspergillus_nidulans_FGSC_A4_

PIAIIGTGCRLPGGSSSPSRLWTLLKNPRVV-ASETPGDR-FDINAFYHEDPG-YPGTTNSK-EAYYISD---
DPRPFDAPFFNISATEAESIDPQQRQLLETVYESLES---AGLR---LDALQGSSTGIFCGVMNNDW-----GELQSA--DYKSLPQYLATGAA-R--
SIIANRISYFFDWHGPSVVVDTACSSSMVALHHAVTALQQNECTLALATGTNLIQAPNIFIS--TTKI-----QML-SPTGRSRMWDAN---A-DGYARGEG-
VISIVLKR--LSDAIRDGDD-IECVIRATGTNQDGRTM-----GLTMPSSVSQLQLIQSTYARAG---LD-
PRRVQDRCQYFEAHGTGTLAGDPQEAAAIHHAFFGPPASPDDNDILLVGSVKTVVGHTEGTAGLAGIIKASLNLQHGLISPNLLFE-TL-NPALE--
PFASHLKVVTELTPWP--SL---PTGA----PRRVSVNSFGFGGTNAHAILESYDAGQSLTAALLPFVFSAASEGSLAAVLNSYAGYL-
KGKPALNLVDFAVTLLRRRS

>EAA64652.1_hypothetical_protein_AN2547.2__Aspergillus_nidulans_FGSC_A4_

PIAVIGIGCRLSGSATDVSGLWDMLKSGRSG-WTPGPGTR-FNMKAFQDPTGT-RSGTTNAT-GGHFIRE---
DISKFDATFFGINPVEAQAMDPQQRLMLEVAYEAFEN---AGIT---MDALWGSNTGVYVGQWASDY-----HEIATR--DIERPPLYLVTGTG-P--
AITSNRVSYVFNLRGPSFTVDTGCSSSLVALHQAVLSLRNRETTQCFVGGVNLLLDPQRFHY--QSRL-----KMF-SKDGRSFPFDAR---A-NGYGRGEG-
VTGVVLKP--LSVALRDGDP-VRAVIRNSVLNQDGR--TP---GISVPSAVAQKEAIIRAYRQAK---LDLYAD------YVEAHGTGTKVGDPIETSAIAAALT------
SPSRPLPIGSIKGNIGHTESAAGLAGLIKSVLMLENGMIPPQVNYE-TT-NPDIH--LEEWNLRIPTKLERQT---------------
LRRISLNSFGYGGTNAHVIIDAAHELSYHSEKPRVFMVSGASEKACQRVCARLARYL-VVNHRALLARLAHTLAKQSI

>EAA67034.1_hypothetical_protein_AN8412.2__Aspergillus_nidulans_FGSC_A4_

LIAIVGSACRFPGQSDSPSKLWTRLKEPIDL-RKTFPPQR-LNLARFYHPDGE-HHGSTDVR-GTSYLLSE--
DPRQFDASFFNINPREAEGMDPQQRLLLETAFEALEA---AGYS---LEAMNGSKTSVHVGVMNSDF-----SNIQLR--DPEVLPTYNATGTA-I--
SILSNRLSYFFNLKGPSVTIDTACSSSLVALHQAVQGLRAGDATAAIVAGANLIFDPAMYIA--ESSL-----HML-SPDSCSRMWDKD---A-NGYARGEG-
VGVLVLKP--LSRAIMDGDH-VEAVIRSTGVNSDGRTK-----GITMPNAESQTELIRQTYRDAG---LDPIRD---
RCQYFECHGTGTATGDPIEARAVHDAFFPTETRTIPDGKLYVGSVKTIIGHLEGCAGIAGVLKAVLAIKNRTIPPNMHFH-EP-NPRVI--PFCDRLEIPTVPIPWP-
-DT---GRS-----PLRASINSFGFGGTNAHAIIEGYDAITPDDLFIGPLLFSANSSSSLVANVKNMAERI-RSDPSIDLESLVWTLYARRS

>EAA67213.1_hypothetical_protein_FG02396.1__Gibberella_zeae_PH1_

-VAIVGLACRFPGDATSPSKFWDLLKSGKDA-YSET-TDR-YNAQAFYHPNSK-RQNVLPVT--GGHFLKQ--
DPHVFDAAFFNITAAEAISLDPKQRIALEVAYEAFEN---AGKP---LKQVAGTTTACFVGSSMSDY--RD-AVVR----DFAHNPKYHVLGTC-E--
EMIANRISHFFDIHGPSATVHTACSSSLVAIHLACQSLLSGDAEMALAGGVGMILTPDGTMQ--LNNL-----GFL-NPEGHSRSFDKD---A-GGYGRGEG-
CGILVLKK--LDKAIQDGDN-IRAVIRASGVNSDGW--TQ---GVTMPSSEAQAALIKHVYETRG---LD-YGA----TQYVEAHGTGTKAGDPVETGAIHR-TI----
GQSKNRKLW-------IGHLEAAAGVASVIKGVLAMENSLIPPNIHFA-SP-NPEIP--LDEWNMAVPTKLTPWP-------AAR-----
TKRMSVSGFGMGGTNGHVVLEAFNSYQPAHNGKRLFTFSSHDQAGLDRVSKSLVDHL--DSLG--------------

>EAA69154.1_hypothetical_protein_FG01790.1__Gibberella_zeae_PH1_

PIAICGMSVRLPGGLHSPQDLWEFLVSKGDA-RGPVPESR-YNASSYWSEKM--KPGTVKTQ-YGYFLDESI-
DIASVDTSFFTMRKDEVGKADPQQRQMLEVARECLED---AG-----EFDWKGRPIGCFMGSFGEDW-----VEMFAK--EAQQYGIYRVMGYG-D--
FMLSNRVSYELDLMGPSQVVRTGCSASLVALHEACLALSRGDCEGAIVGGANLIMAPGMTVA--MTEQ-----GVL-SPDGSCKTFSAD---A-NGYARGEA-
ISAIFVKP--LSAAIRDGNP-IRAVIRSTASNSDGRGTAG---GIQVPNDIAQETLIRRAYKMAG---IDDYSQ----TAFVECHGTGTAIGDPIETKAVGR-VF------
GPSGGVLIGSVKPNLGHSEGASGLTSLIKAVMALENRTIPPNIKLS-QP-NPAIP--WTSCGLSVPTEPTTWP--SS---K-------
HERISVNSFGIGGTNAHVIIDSACSVGRSATAPQLLVFSANGTDSLRQMITNYENYA-EKNPE-KIDDLAFTLGHKRE

>EAA69820.1_hypothetical_protein_FG10548.1__Gibberella_zeae_PH1_

PIAVVGIGCRFPGNSSNPEALWKLLSDARSC-YSKVPPDR-YNVDGFRHPS-N-KLNTTIAE--GAHFLSE--
NIAAFDAAFFNIAPIDAKSMDPQQRMLLEVVYEGLES---AGIK---MEDTTGSDTSCYVGTFTRDW--SD-MLMR----DPETAPKYSGAGIG-S--
GMQANRVSWFFDWHGPSLTLDTACSSSLVALHLACQSINDGVSKVAVAAGTTLMLNPDMPMW--MSNM-----SFL-SADGLSKSFDAS---A-DGYGRGEG-
IAAVILKS--LDQAVRDRDP-IRAVIRGTGINHDGH--TP---GITLPSASAQADLISSVYKKSG---LD-YDD----TMYFEAHGTGTAAGDSVELRAIGA-SV----
TKDSSKTLVVGSIKSNIGHTEGAAGLAAFIKAVLVVEKGLIPRNIHLN-NP-NPRIP--FEELKIQVPTVEIPWP-------TPG-----
VRRASVNSFGYGGTNAHAILDDASSSFKETTNKRLFVFSAHEESAALNMKQRYATHL--EEEASYLDRLAY-------

>EAA71995.1_hypothetical_protein_FG08795.1__Gibberella_zeae_PH1_

DIAVVGIGLRFPGDASSPEELWKVLERGESQ-WSEFPKDR-LNIDGYYHPGGD-RQGSISFR--GAHFIKS--
DFAAFDASFFAVAAEDAKAIDPQQRILLEASYEALEN---AGIR---KEDVDGTDAAVYVGSFVKDY--EQ-VCLR----DPDWQPQYAATGNG-I--
AIMANRISYFFNLHGPSMTIDTGCSGSLVSVHLAAQSLRTKETSLAIAAGAGMILTPNTMMP--MTAL-----NFL-SPDGKCFTFDSR---A-NGYGRGEG-
IGVVVMKR--LSDALRDNDT-IRAVIRATKVNQDGH--TTALKGITLPSKEAQVANIHSVYESAG---LD-FSQ----TGYVECHGTGTKAGDWRELKAISE-SL----
GSGIDNPIVVGSLKPNIGHLEGAAGVAGLIKGVLTLEHAKIPPNINLD-KP-NPDID--FKNWKVKVPTKLLDWP-------LPG-----
LRRVSVNCFGFGGTNAHVIMDEAPR--------QLFLFSAHERSGVQRVMESHLPYL--KSKTF-LRDYSYTLGCRRS

>EAA73063.1_hypothetical_protein_FG08208.1__Gibberella_zeae_PH1_

PIAVVGMSCRLPGDATDTQKLWELITKGRSA-WSKVPTDR-WNQEAFHDPAAEGKPGR-TSTDGGHFLKD---
DIKAFDASFFGVNPIEATAMDPQQRLVLEIAYEAFEN---AGLT---LQQLSGSNTGVYVGLWASDY-----QEMLLR--DIDFPPIYQASGVG-A--
AIASNRVSYCFNLHGPSLTLDTGCSASLVALHQAVHSLRAGETDKCFVAGVNLQLDPQRYGY--QNKL-----SMF-SKQGKSFTFDHRAKGA-SGYGRGEG-
CSGVVLMP--LSKAQKQGFP-IRAVIRNSVANQDGKTN-----GITVPSAAAQSAAIEKAYAQVG---LT-PYA-----DYVEAHGTGTTVGDPIEAKAIAK-VL--
GAGRQSDSPLPIGSLKANIGHTESAAGLTGLVKAVLMLENNMIPPQVNYE-KP-NPEID--LDALNLRIPISLENKP--------------
LKRISVNSFGYGGTNAHVVVDAADAKDTLRVREQLFVLSAASEKSSQDLVLNVAKYLESKADAALLSRLAYTLSRRTV

>NP_001012687.1_fatty_acid_synthase__Bos_taurus_

EVVITGMSGKLPES-ENLEEFWANLIGGVDM---VTDDDRRWKAGLYGLPRRS-GKLK--------------
DLSRFDASFFGVHPKQAHNMDPQLRLLLEVTYEAIVD---AGIN---PASIRGTNTGVWVGVSGSEA-----SEALSR--DPETLVGYSMVGCQ-R--
AMLANRLSFFFDFKGPSITLDTACSSSLLALQRAYQAIQRGECAMAIVGGVNIRLKPNTSVQ--FMKL-----GML-SPEGTCKFFDAS---G-NGYCRAKA-
VMAILLTK--KSLARR-----VYATILNAGTNTDGCKEKG----VTFPSGEAQEQLISSLYKPAG---LD-PET----LEYVEAHGTGTKVGDPQELNGIVQ-AL----
CGTRQSPLRIGSTKSNMGHPEPASGLAALAKVLLSLEHGLWAPNLHFH-NP-NPKIP-ALQDGRLQVVDRPLPVL--------------
GGNVGINSFGFGGSNVHVILQPNSQPAPHAALPRLLRASGRTLEGVQGLLELGLQHS-QN---LAFVSMLNDIATPSP

>NP_001041530.1_uncharacterized_protein_LOC100000781__Danio_rerio_

EIAIVGVGCHFPGG-EGLENFWRVLLEGKNC-AVQIPNDR-FNLSQWYDPEES-KAGKTYTA-KAALID----
GLNAFDHKFFGITEAESLSMDPQHKLLLQCTYRALED---AGIP---MEKASGTRTGVYLGLMNRDF--ELATVRN----NPKHISHTTGTGVA-M--
SIAANRISYIFNFTGPSVAIDCACSSSLVALHFACQAIKQGDCDMSVCGGVSCILEPALFVA--LSKA-----KMI-SPDGTSKPFSSK---A-DGYGRGEG-
CGVVLLKP--LKRALEDHDH-IWGVIGKTAVNQDGHSVSP----ITKPSMVQQEELLRKIYST-E---AD-LCS----VQYIEAHGTGTPVGDPVEAGSMSK-VI----
AKQKSGPLIIGSVKSNIGHTESAAGVAGLIKVLLMMQHETIVPSLFYS-KE-NSSID--LKSLNMNIPTRVQKWI--SN---N-----
RAGRIAGINNFGFGGTNAHAIVKQYVQSQPISKSCQYFVVSAASEKSMRNIIRDTADQI--GAGKSDLQSLLYTSACRRS

>NP_001093400.1_fatty_acid_synthase__Sus_scrofa_

EVVIAGMSGKLPES-ENLEEFWANLIGGVDM---VTADDRRWKAGLYGLPR---RMGKLK------------
DLSRFDASFFGVHSKQANTMDPQLRMLLEVTYEAIVD---GGIN---PASLRGTSTGVWVGVSSSDA-----SEALSR--DPETLVGYSMIGCQ-R--
AMMANRLSFFFDFKGPSITIDTACSSSLLALQSAYQAIRGGECSAAVVGGLNVLLKPNSSLQ--FMKL-----GML-SQDGTCRSFDAE---G-TGYCRAEA-
VVAVLLTK--KSLARR-----VYATILNAGTNTDGSKEQG----VTFPSGDVQEQLIRSLYAPAG---PD-PES----LEYIEAHGTGTKVGDPQELNGIVN-AL----
CATRREPLLIGSTKSNMGHPEPASGVAALIKVLLSLEHGVWAPNLHYH-TP-NPEIP-ALQDGRLQVVDRPLPIR--------------
GGNVGINSFGFGGSNVHVILQPNSRPAQHAALPRLLQASGRTLEAVQTLLEQGLRHS-RD---LAFVGMLNEIAAVSP

>NP_001137778.2_fatty_acid_synthase_1__isoform_C__Drosophila_melanogaster_

EIAITGFSGRLPES-STIEEFKQNLFDGVDM---VNDDPRRWERGLYGLPD---RIGKLKDS-----------DLENFDQQFFGVHQKQAECMDPLLRMLLELTHEAIID---
AGLN---PSDLRGSRTGVYIGVSNSET-----EQHWCS--DADRVNGYGLTGCA-R--
AMFANRISFTFDFKGPSYSIDTACSSSLYALEQAFSDMREGKVDNALVAGAGLILKPTMSLQ--FKRL-----NML-SPDGSCKAFDES---G-NGYVRSDG-
CVVLLLQR--TSAARR-----VYASILNVRTNTDGFKEQG----ITYPIGKMQNRLIRETYEEIG---LN-PAD----VVYVEAHGTGTKVGDPQEVNSITD-FF----
CKDRTTPLLIGSVKSNMGHSEPASGVCSVAKILIAMEEGVIPGNLHYN-KP-NPDLY-GLVDGRLKVVDRNLPWN--------------
GGIIGLNSFGFGGANAHVILKSNPKLTPKDGALKVVLASGRTFEAVEQLLESASTNA-DDDEYLQLINEIHSKAIPNH

>NP_001239013.1_polyketide_synthase_2__Strongylocentrotus_purpuratus_

KVAIVGIGCRYANGINGVRKFWEMLAKGMDC-TTPPPSDR-FDSSFFLFPGSK-IQGKMYNK-CAGYLSQ---
NPEHFDRQFFRISPEEANHLDPQIRMLLEVVWESLQD---AGIP---AHSARGSKTGVYMGVTASEY--GV-LIGM----PNDNINQYTNSGTN-S--
CMTANRVSYEFDFRGPSFTVDTACSSSMYSVHLACEALKNGQCEMAVAGGVNVSLLPITSIG--FCQA-----GML-APDGKCKSFDKS---A-DGYARGEG-
VGAVILKP--LQRAIDDGDR-IYGVIRGGAIANDGR--TP---GIALPSYDGQVSLIENAYKNAN---TL-PEE----VVYVEAHGTGTQVGDRTEASAIGE-AM----
GLVEQPPLYLGSVKSNFGHSEGAAGIAGVIKAALCLYHEQIPKVVHFR-SG-NESVD--FESLNIRVPGELTRWP-------KAA-----
KKLVGCSSFGFGGANAHIVLEG---NERDSGKPSILFLSAASKDALKQHLEEWGSYL--NEVIRAYNNALYTAAVRST

>NP_004095.4_fatty_acid_synthase__Homo_sapiens_

EVVIAGMSGKLPES-ENLQEFWDNLIGGVDM---VTDDDRRWKAGLYGLPRRSGKLK----------------
DLSRFDASFFGVHPKQAHTMDPQLRLLLEVTYEAIVD---GGIN---PDSLRGTHTGVWVGVSGSET-----SEALSR--DPETLVGYSMVGCQ-R--
AMMANRLSFFFDFRGPSIALDTACSSSLMALQNAYQAIHSGQCPAAIVGGINVLLKPNTSVQ--FLRL-----GML-SPEGTCKAFDTA---G-NGYCRSEG-
VVAVLLTK--KSLARR-----VYATILNAGTNTDGFKEQG----VTFPSGDIQEQLIRSLYQSAG---VA-PES----FEYIEAHGTGTKVGDPQELNGITR-AL----
CATRQEPLLIGSTKSNMGHPEPASGLAALAKVLLSLEHGLWAPNLHFH-SP-NPEIP-ALLDGRLQVVDQPLPVR--------------
GGNVGINSFGFGGSNVHIILRPNTQPAPHATLPRLLRASGRTPEAVQKLLEQGLRHS-QD---LAFLSMLNDIAAVPA

>NP_032014.3_fatty_acid_synthase__Mus_musculus_

EVVIAGMSGKLPES-ENLQEFWANLIGGVDM---VTDDDRRWKAGLYGLPKRS-GKLK--------------
DLSKFDASFFGVHPKQAHTMDPQLRLLLEVSYEAIVD---GGIN---PASLRGTNTGVWVGVSGSEA-----SEALSR--DPETLLGYSMVGCQ-R--
AMMANRLSFFFDFKGPSIALDTACSSSLLALQNAYQAIRSGECPAALVGGINLLLKPNTSVQ--FMKL-----GML-SPDGTCRSFDDS---G-SGYCRSEA-
VVAVLLTK--KSLARR-----VYATILNAGTNTDGSKEQG----VTFPSGEVQEQLICSLYQPAG---LA-PES----LEYIEAHGTGTKVGDPQELNGITR-SL----
CAFRQAPLLIGSTKSNMGHPEPASGLAALTKVLLSLEHGVWAPNLHFH-NP-NPEIP-ALLDGRLQVVDRPLPVR--------------
GGNVGINSFGFGGSNVHVILQPNTRPTAHAALPHLLHASGRTLEAVQDLLEQGRQHS-QD---LAFVSMLNDIAATPT

>NP_059028.1_fatty_acid_synthase__Rattus_norvegicus_

EVVIAGMSGKLPES-ENLQEFWANLIGGVDM---VTDDDRRWKAGLYGLPK---RSGKLK-------------
DLSKFDASFFGVHPKQAHTMDPQLRLLLEVSYEAIVD---GGIN---PASLRGTNTGVWVGVSGSEA-----SEALSR--DPETLLGYSMVGCQ-R--
AMMANRLSFFFDFKGPSIALDTACSSSLLALQNAYQAIRSGECPAAIVGGINLLLKPNTSVQ--FMKL-----GML-SPDGTCRSFDDS---G-NGYCRAEA-
VVAVLLTK--KSLARR-----VYATILNAGTNTDGCKEQG----VTFPSGEAQEQLIRSLYQPGG---VA-PES----LEYIEAHGTGTKVGDPQELNGITR-SL----
CAFRQSPLLIGSTKSNMGHPEPASGLAALTKVLLSLENGVWAPNLHFH-NP-NPEIP-ALLDGRLQVVDRPLPVR--------------
GGIVGINSFGFGGANVHVILQPNTQPAPHAALPHLLHASGRTMEAVQGLLEQGRQHS-QD---LAFVSMLNDIAATPT

>NP_492417.2_Fatty_Acid_SyNthase__Caenorhabditis_elegans_

DIVISGVSGRFPRC-DNVKMFGDMLLAGEDL---VTEDSLRWTPGFCDLPKRH-GKLK--------------
VLNKFDAGFFQVTPKQANFMDPQVRLLLEASWEAMVD---AGIN---PTDLRGSKTGVFVGCSASET-----SGMLTQ--DPDTVTGYTLTGCV-R--
SMFSNRISYTFDLQGPSFSVDTACSSSLLALQLAVDSIRQGQCDAAIVAGAHLTLTPTAALQ--FLRL-----GML-TDKGSCRSFDES---G-DGYCRTEG-
VAAIFIQR--KKKAQR-----LYATVVHAKSNTDGHKEHG----ITFPSGERQAQLLQEVYSEAG---ID-PNS----VYYVEAHGTGTKVGDPQEANAICE-VF----
CSKRTDSLLIGSVKSNMGHAEPASGVCSLTKILLSIERQLIPPNLHYN-TP-NQYIP-GLTDGRLKVVTEPTALP--------------
GGLIGINSFGFGGSNTHVILKAADHPITPHPFTKLVTYCGRTQEAVENIFTEIESNKDD----LYLQALLANQANMPA

>NP_608748.1_fatty_acid_synthase_1__isoform_A__Drosophila_melanogaster_

EIAITGFSGRLPES-STIEEFKQNLFDGVDM---VNDDPRRWERGLYGLPD---RIGKLKDS-----------DLENFDQQFFGVHQKQAECMDPLLRMLLELTHEAIID---
AGLN---PSDLRGSRTGVYIGVSNSET-----EQHWCS--DADRVNGYGLTGCA-R--
AMFANRISFTFDFKGPSYSIDTACSSSLYALEQAFSDMREGKVDNALVAGAGLILKPTMSLQ--FKRL-----NML-SPDGSCKAFDES---G-NGYVRSDG-
CVVLLLQR--TSAARR-----VYASILNVRTNTDGFKEQG----ITYPIGKMQNRLIRETYEEIG---LN-PAD----VVYVEAHGTGTKVGDPQEVNSITD-FF----
CKDRTTPLLIGSVKSNMGHSEPASGVCSVAKILIAMEEGVIPGNLHYN-KP-NPDLY-GLVDGRLKVVDRNLPWN--------------
GGIIGLNSFGFGGANAHVILKSNPKLTPKDGALKVVLASGRTFEAVEQLLESASTNA-DDDEYLQLINEIHSKAIPN-

>NP_990486.2_fatty_acid_synthase__Gallus_gallus_

DVVIAGIAGKLPES-ENLQEFWENLLNGVDM---VTEDDRRWKPGIYGLPKRN-GKLK--------------DIKKFDASFFGVHPKQAHTMDPQLRLLLEVSYEAILD--
-GGIN---PTALRGTDTGVWVGASGSEA-----AEALSQ--DPEELLGYSMTGCQ-R--
AMLANRISYFYDFTGPSLTIDTACSSSLMALENAYKAIRHGQCSAALVGGVNILLKPNTSVQ--FMKL-----GML-SPDGACKAFDVS---G-NGYCRSEA-
VVVVLLTK--KSMAKR-----VYATIVNAGSNTDGFKEQG----VTFPSGEMQQQLVGSLYRECG---IK-PGD----VEYVEAHGTGTKVGDPQEVNGIVN-VF----
CQCEREPLLIGSTKSNMGHPEPASGLAALAKVILSLEHGLWAPNLHFN-DP-NPDIP-ALHDGSLKVVCKPTPVK--------------
GGLVSINSFGFGGSNAHVILRPNEKPQETCNLPRLVQVCGRTQEAVEILIEESRKHGGC----SPFLSLLSDISAVPV

>pdb|3HHD|D_Chain_D__Fatty_acid_synthase

EVVIAGMSGKLPES-ENLQEFWDNLIGGVDM---VTDDDRRWKAGLYGLPR---RSGKLK-------------
DLSRFDASFFGVHPKQAHTMDPQLRLLLEVTYEAIVD---GGIN---PDSLRGTHTGVWVGVSGSET-----SEALSR--DPETLVGYSMVGCQ-R--
AMMANRLSFFFDFRGPSIALDTACSSSLMALQNAYQAIHSGQCPAAIVGGINVLLKPNTSVQ--FLRL-----GML-SPEGTCKAFDTA---G-NGYCRSEG-
VVAVLLTK--KSLARR-----VYATILNAGTNTDGFKEQG----VTFPSGDIQEQLIRSLYQSAG---VA-PES----FEYIEAHGTGTKVGDPQELNGITR-AL----
CATRQEPLLIGSTKSNMGHPEPASGLAALAKVLLSLEHGLWAPNLHFH-SP-NPEIP-ALLDGRLQVVDQPLPVR--------------
GGNVGINSFGFGGSNVHIILRPNTQPAPHATLPRLLRASGRTPEAVQKLLEQGLRHS-QD---LAFLSMLNDIAAVPA

>pdb|5BP1|A_Chain_A__Mycocerosic_Acid_Synthase

PVAIIGMACRLPGAINSPQQLWEALLRGDDF-VTEIPTGR-WDAEEYYDPEPG-VPGR-SVSKWGAFLD----
DPAAFDPEFFGITEREAAAIDPQHRLLLETAWEAVEH---SGLN---PAGLAGSATGVFMGLTHNDY-----AHLAAD--AKALEGPYGFTGTS-F--
SLASGRIAYALGVHGPAITVDTACSSSLSAIHMACRSLHDGESDVALAGGVSVLLEPRKAAG--GSAA-----GML-SPTGHCHAFDTA---A-DGFVSAEG-
CVVLTLKR--LDDAVADGDR-ILAVIRGTATNQDGRTV-----NIATPSADAQAKVYRMALKAAG---VE-PGT----VGLVEAHGTGTPVGDPLEFSSLAE-VY------
GTDGPCALGSIKTNFGHTQSAAGALGVMKAVLALQHNVIPQNLHFT----RLPDQMAEIETGLFVPETITPWPVREGQ----------
PRRAAVSAYGLSGTNVHAVLEQAPEISPKAGNALVFPVSASSADALRSTAQHLADWLLRSGD----------------

>sp|A0A089QRB9.2|MSL3_MYCTU__Mycocerosic_acid_synthase-like_polyketide_synthase_

SVAVIGMACRLPGGIDSPQRLWEALLRGDDL-VGEIPADR-WDANVYYDPEPG-VPGR-SVSRWGAFLD----
DVGGFDCDFFGLTEREATAIDPQHRLLLEVSWEAIEH---AGVD---PATLAESQTGVFVGLTHGDY-----ELLSAD--CGAAEGPYGFTGTS-N--
SFASGRVAYTLGLHGPAVTVDTACSSGLTAVHQACRSLDDGESDLALAGGVVVTLEPRKSVS--GSLQ-----GML-SPTGRCHAFDEA---A-DGFVSGEG-
CVVLLLKR--LPDAVRDGDR-VLAIVRGTAANQDGRTV-----NIAAPSAQAQIAVYQQALAAAG---VE-AST----VGMVEAHGTGTPVGDPVEYASLAA-VY-----
-GTEGPCALTSVKTNFGHLQSASGPLGLMKTILALRHGVVPQNLHFC----RLPDQLAEIDTELFVPQANTSWPDNTGQ----------
PRRAAVSSYGMSGTNVHAILEQAPVLTPEAGGLALFPVSATSAEQLHVTAARLADWVDQNGNAVSMRDLGYTLSCRRA

>sp|A0R1E8.1|PKS5_MYCS2_RecName__Full_Mycocerosic_acid_synthase-like_polyketide_synthase

PVAIIGMACRLPGAINSPQQLWEALLRGDDF-VTEIPTGR-WDAEEYYDPEPG-VPGR-SVSKWGAFLD----
DPAAFDPEFFGITEREAAAIDPQHRLLLETAWEAVEH---SGLN---PAGLAGSATGVFMGLTHNDY-----AHLAAD--AKALEGPYGFTGTS-F--
SLASGRIAYALGVHGPAITVDTACSSSLSAIHMACRSLHDGESDVALAGGVSVLLEPRKAAG--GSAA-----GML-SPTGHCHAFDTA---A-DGFVSAEG-
CVVLTLKR--LDDAVADGDR-ILAVIRGTATNQDGRTV-----NIATPSADAQAKVYRMALKAAG---VE-PGT----VGLVEAHGTGTPVGDPLEFSSLAE-VY------
GTDGPCALGSIKTNFGHTQSAAGALGVMKAVLALQHNVIPQNLHFT----RLPDQMAEIETGLFVPETITPWPVREGQ----------
PRRAAVSAYGLSGTNVHAVLEQAPEISPKAGNALVFPVSASSADALRSTAQHLADWLLRSGDG---------------

>sp|B2HIL7.1|MSL7_MYCMM_RecName__Full_Phenolphthiocerol_synthesis_polyketide_synthase_type_I_

PVAVVGIGCRFPGGADGPEGLWDLVSQGRDA-VTEFPNDRGWDTEGLFDPDPD-AEGKTYTR-WGAFVE----
NATNFDAGFFGIPPSEVLAMDPQQRLMLEVSWEALEH---AGID---PMSLRGSSTGVFTGIFAPSY--------GGK--DVGALQGYGLTGSP-V--
SVASGRVAYVLGLEGPALSVDTACSSSLVAIHWAMASLRSGECDMALAGGVTVMGLPSIFVG--FSRQ-----RGL-AADGRCKAFAAA---A-DGTGWGEG-
AGVLVLER--LSDAQRNGHN-VLAVVRGSAINQDGASN-----GLTAPNGLAQQRVIQAALANCG---LT-SAD----VDVVEAHGTATTLGDPIEAEALLA-TY----
GQPTDQPLWVGSIKSNMGHTQAAAGVAGVIKMVQAMRHGLMPASLHVD-EP-SKRVD--WESGAVSVLAEARDWP--DA---GR------
PRRAGVSSFGISGTNAHVILEEAPAKGEPSLPVVPWVISARSAEALTAQAGRLLAHV-QADPQSNPVDIGFSLAGRSA

>sp|O31782.3|PKSN_BACSU_RecName__Full_Polyketide_synthase_PksN

PIAIVGISGIFPMA-KDVEAYWNILKEGKDC-MTEIPKDR-WDWREYEGDPAK-EVNKTNVK-WGGFID----
GIADFDPLFFGISPREAEQMEPQQRLLLTYAWKAIED---AGYS---AKRLSGTKTGVFIGTGNTGY-----SSLLSK--ANSAIEGSAAANTS-P--
SVGPNRVSYFLNLHGPSEPVDTACSSSLVAIHHAISSIEEGTCDMALAGGVNTIILPEVYIS--FDKA-----GAL-SKEGKCKTFSNQ---A-DGFAHGEG-
AGILFLKK--LKAAEEAGDH-IYGVIKGSAINHGGRAAS-----LTTPNPKAQADVIQSAYQKAG---ID-PKT----VTYIEAHGTGTELGDPVEINGLKS-
AFKALGVNSANPYCGLGSVKTNIGHLSLAAGAAGVIKILLQLKHKTLVKSLHCE--

>sp|O31784.2|PKSR_BACSU_RecName__Full_Polyketide_synthase_PksR

GIAIIGMSGQFPKA-NSVTEFWDNLVQGKNC-VSEVPKER-WDWRKY--AAAD-KEGQSSLQ-WGGFIE----
GIGEFDPLFFGISPKEAANMDPQEFLLLIHAWKAMED---AGLT---GQVLSSRPTGVFVAAGNTDT-----------------------AVV-P--
SLIPNRISYALDVKGPSEYYEAACSSALVALHRAIQSIRNGECEQAIVGAVNLLLSPKGFIG--FDSM-----GYL-SEKGQAKSFQAD---A-NGFVRSEG-
AGVLIIKP--LQKAIEDSDH-IYSVIKGSGVSHGGRGMS-----LHAPNPAGMKDAMLKAYQGAQ---ID-PKT----VTYIEAHGIASPLADAIEIEALKS-GC----SQ-
EEAPCYISSLKPSIGHGELVSGMAALMKVSMAMKHQTIPGISGFS-SL-NDQVS--LKGTRFRVTAENQQWR--DL---
SDDAGKKIPRRASINSYSFGGVNAHVILEEYIPVSMSENGAHIVVLSAKNQDRLKAIAQQQLDYV--NKQQLSLQDYAYTLQTGRE

>sp|O53901.2|PKS5_MYCTU_RecName__Full_Mycocerosic_acid_synthase-like_polyketide_synthase

PVAVIGMGCRLPGGIDSPDRLWEALLRGDDL-VTEIPADR-WDIDEYYDPEPG-VPGRTDCK-WGAYLD----
NVGDFDPEFFGIGEKEAIAIDPQHRLLLETSWEAMEH---GGLT---PNQMAS-RTGVFVGLVHTDY-----ILVHAD--NQTFEGPYGNTGTN-A--
CFASGRVAYAMGLQGPAITVDTACSSGLTAIHLACRSLHDGESDIALAGGVYVMLEPRRFAS--GSAL-----GML-SATGRCHAFDVS---A-DGFVSGEG-
CVMLALKR--LPDALADGDR-ILAVIRGTAANQDGHTV-----NIATPSRSAQVAAYREALDVAG---VD-PAT----VGMVEAHGPGTPVGDPIEYASLAE-VY------
GNDGPCALASVKTNFGHTQSAAGALGLMKAVLALQHGVVPQNLHFT----ALPDKLAAIETNLFVPQEITPWPGADQ---------
ETPRRAAVSSYGMTGTNVHAIVEQAPVATPGIDGALLFALSASSQDALRQTAARLADWVDAQGPELAPADLAYTLARRRG

>sp|P16540.1|KAS1_STRVN_RecName__Full_Granaticin_polyketide_putative_beta-ketoacyl_synthase_1_AltName__Full_ORF1

RVVITGVGVRAPGG-SGTKEFWDLLTAGRTA----------TRPISFFDASPFRSRIA---------------GEIDFDAVAEGFSPREVRRMDRATQFAVACTRDALAD---
SGLD---TGALDPSRIGVALGSAVAYLVMSDSGREWLV--DPAHLSPMMFDYLS-P--
GVMPAEVAWAAGAEGPVTMVSDGCTSGLDSVGYAVQGTREGSADVVVAGAADTPVSPIVVAC--FDAI-KATTPRNDDPAHASRPFDGT---R-NGFVLAEG-
AAMFVLEE--YEAAQRRGAH-IYAEVGGYATRSQAYHMTG-----LKKDGREMAESIRAALDEAR---LD-RTA----VDYVNAHGSGTKQNDRHETAAFKRSLG---
---EHAYAVPVSSIKSMGGHSLGAIGSIEIAASVLAIEHNVVPPTANLH---

>sp|P16541.2|KAS2_STRVN_RecName__Full_Granaticin_polyketide_putative_beta-ketoacyl_synthase_2_AltName__Full_ORF2

RAVVTGLSVAAPGG-LGTERYWKSLLTGENG----IAELSRFDASRYPSRLAG-------------------QIDDFEAS-EHLPSRLLPQTDVSTRYALAAADWALAD---
AGVG---PESGLDDDLGVVTSTAQGDFTHREFHKLWSQ--GPAYVSVYESFAWF-Y--
AVNTGQISIRNTMRGPSAALVGEQAGGLDAIGHARRTVRRGPGWCSAVASTRRSTRGASSSQ--LSGG---LVSTVADPERAYLPFDVD---A-SGYVPGEG-
GAVLIVED--ADSARARGAERIYV---RSPLRRDPAPGSG--------RPPALGRAAELALAEAG---LT-PAD----ISVVFADGAGVPELDRAEADTLAR-LF----GP---
RGVPVTAPKALTGRLCAGGGPADLAAALLALRDQVIPATGRH---

>sp|P40806.3|PKSJ_BACSU_RecName__Full_Polyketide_synthase_PksJ_Short_PKS

PVAIVGISGRFPGA-MDIDEFWKNLEEGKDS-ITEVPKDR-WDWREHYGNPDT-DVNKTDIK-WGGFID----
GVAEFDPLFFGISPREADYVDPQQRLLMTYVWKALED---AGCS---PQSLSGTGTGIFIGTGNTGY-----KDLFHR--ANLPIEGHAATGHMIP--
SVGPNRMSYFLNIHGPSEPVETACSSSLVAIHRAVTAMQNGDCEMAIAGGVNTILTEEAHIS--YSKA-----GML-STDGRCKTFSAD---A-NGYVRGEG-
VGMVMLKK--LEDAERDGNH-IYGVIRGTAENHGGRANT-----LTSPNPKAQADLLVRAYRQAD---ID-PST----VTYIEAHGTGTELGDPIEINGLKA-
AFKELSNMVPDHRCGIGSVKSNIGHLELAAGISGLIKVLLQMKHKTLVKSLHCE-TL-NPYLQ--LTDSPFYIVQEKQEWK--SV---
TDRDGNELPRRAGISSFGIGGVNAHIVIEEYMPEHTATEQPNVIVLSAKNKSRLIDRASQLLEVI-RNKKYQDLHRIAYTLQVGRE

>sp|P40872.4|PKSM_BACSU_RecName__Full_Polyketide_synthase_PksM

DIAIIGMAGRYPKA-KNIQEFWEQLKAGTDC-ITEIPNSR-WEWKESDGLDSP--AGKPLSK-WGGFIE----
EADCFDPQFFRISPREAEMMDPQERLFLETCWEAIED---AGYT---PETIASPHVGVFAGVMHKDY-----SLIGAE--ALSEHNPFPLSLNYAQ----
IANRVSYYCNFHGPSMAVDTVCSSSLTAVHLAIESIRNGECEAALAGGVNLSLHPAKYIS--YGSV-----GMH-SSDGYCHTFGKG---G-DGYVSGEG-
VGTVLLKP--LRKAEQDGDR-IYAVIKGSAINHVGKVS-----GITVPSPVAQADVIEACLEKTG---ID-PRT----ISYVEAHGTGTSLGDPIEVQGLVK-AFSRNTQD--
KQFCSIGSVKSNIGHAEAAAGISGLTKTVLQLHHKTLVPSLHSE-EL-NPYLK--LDQTPFFVQHETKEWE--
QPSFTENGVDVTYPRRAGLSSFGASGSNAHLILEEYIPQTPKDHPPALIVLSAKNMERLQEKAEQLLTAI-KQKRYTDLIRIAYTLQTGRE

>sp|P41175.1|KAS1_STRCM_RecName__Full_Putative_polyketide_beta-ketoacyl_synthase_1_AltName__Full_ORF1

RVAITGIEVLAPGG-LGRKEFWQLLSEGRTA----------TRGITFFDPAPFRSKVA---------------AEADFCGLENGLSPQEVRRMDRAAQFAVVTA-RAVED---
SGAE---LAAHPPHRIGVVVGSAVGYRVVSDGGRLDLV--DHRYAVPHLYNYLV-P--
SSFAAEVAWAVGAEGPSTVVSTGCTSGIDAVGIAVELVREGSVDVMVAGAVDAPISPIPCVL-DAIKA---TTPRHDAPATASRPFDST---R-NGFVLGEG-
AAFFVLEE--LHSARRRGAH-IYAEIAGYATRSNAYHMTG------LRDGAEMAEAIRLALDEAR---LN-PEQ----VDYINAHGSGTKQNDRHETAAFKKALG------
EHAYRTPVSSIKSMVGHSLGAIGSIEIAASALAMEYDVVPPTANLH---

>sp|P41176.1|KAS2_STRCM_RecName__Full_Putative_polyketide_beta-ketoacyl_synthase_2_AltName__Full_ORF_2

PVAVTGMGIAAPNGLGRPTTGRPPWAPRAAS-----AASTRFDPSGYPAQLAG-------------------EIPGFRAA-EHLPGRLVPQTDRVTRLSLAAADWALAD---
AGVE---VAAFDPLDMGVVTASHAGEFGQDELQKLLGQ--GQPVLSAYQSFAWF-Y--AVNSGQISIRHGMKGPSGVVVSDQAGGLDALAQARRLVRKG-
TPLIVCGAVEPRSAPGAGSP--SSPA--GGMSDSDEPNRAYLPFDRD---G-RGYVPGGGRGVVPPLER--AEAAPARGAE-VYGEAGPLARLPAPHSGRG---------
STRAHAIRTALDDAG---TA-PGD----IRRVFADGGGRYPNDRAEAEAISE-VF-------GPGRVPVTCPRTMTGRLHSGAAPLDVACALLAMRAGVIPPTVHID-----
----

>sp|P43678.2|KAS1_STRRM_RecName__Full_Oxytetracycline_polyketide_putative_beta-ketoacyl_synthase_1

RVVITGIGVVAPGD-VGTKPFWEMLTAGRTA----TRPISSFDASPFRSQVA---------------------AECDFDPAAEGLSQRQVRAWDRTMQFAYVAAREALAD---
SGVT-----GEADPRTGVMAGTACGYAVVSDEGRLWQV--DDAHGVPYLYDYFV-P--
SSMAAEIAWLAEAEGPAGVVSAGCTSGIDVLTHAADLVRDGAAEVMVAGASDAAISPITVAC--FDAI-KATTPRNDEPETASRPFDRT---R-NGFVLGEG-
AAFFVLEE--YAHARRRGAR-AYAEIAGYAGRCNAYSMTG-----LRSDGRELAEAVSRALDIAR---VD-PSE----VDYVNAHGSATKQNDLHETAAFKRSLG-----
-PHAYSVPISSIKSMIGHSLGAICALEVAASALRIEHGVIPPTANLR--

>sp|P96202.2|PPSC_MYCTU_RecName__Full_Phthiocerol_synthesis_polyketide_synthase_type_I

PIAVIGMGCRFPGGVNNPEQFWDLLCAGRSG-IVRVPAQR-WDADAYYCDDHT-VPGTICST-EGGFLTSW--
QPDEFDAEFFSISPREAAAMDPQQRLLIEVAWEALED---AGVP---QHTIRGTQTSVFVGVTAYDYMLTLAGRLR-----PVDLDAYIPTGNS-A--
NFAAGRLAYILGARGPAVVIDTACSSSLVAVHLACQSLRGRESDMALVGGTNLLLSPGPSIA--CSRW-----GML-SPEGRCKTFDAS---A-DGYVRGEG-
AAVVVLKR--LDDAVRDGNR-ILAVVRGSAVNQDGASS-----GVTVPNGPAQQALLAKALTSSK---LT-AAD----IDYVEAHGTGTPLGDPIELDSLSK-
VFSDRAGS---DQLVIGSVKTNLGHLEAAAGVAGLMKAVLAVHNGYIPRHLNFH-QL-TPHAS--EAASRLRIAADGIDWP--TT---GR------
PRRAGVSSFGVSGTNAHVVIEQAPDQRGPVPAVSTLVVFGKTAPRVAATASVLADWL--DGPGVPLADVAHTLNHHRA

>sp|P9WQE0.1|PPSE_MYCTO_RecName__Full_Phthiocerol_synthesis_polyketide_synthase_type_I

AIAVVGMAGRFPGA-KDVSAFWSNLRRGKES-IVTLSEQE-LRDAGVSDKTLA-DP--AYVR-RAPLLD----
GIDEFDAGFFGFPPLAAQVLDPQHRLFLQCAWHALED---AGAD---PARFDGS-IGVYGTSSPSGYLLHNLLSHR----DPNAVLAEGLNFDQ-F--
SLLATRISHAFNLRGPSIAVQTACSSSLVAVHLACLSLLSGECDMALAGGSSLCIPHRVGYF--TSPG-----SMV-SAVGHCRPFDVR---A-DGTVFGSG-
VGLVVLKP--LAAAIDAGDR-IHAVIRGSAINNDGSAKMG----YAAPNPAAQADVIAEAHAVSG---ID-SST----VSYVECHGTGTPLGDPIEIQGLRA-AF----
EVSRSAPCVLGSVKSNIGHLEVAAGIAGLIKTILCLKNKALPATLHYT-SP-NPELR--LDQSPFVVQSKYGPWE--CD---G-------
VRRAGVSSFGVGGTNAHVVLEEAPAAHAEPAGPQVILLSAQTAAALGESRTALAAAL--ETQDPRLSDVAYTLARRRK

>sp|P9WQE1.1|PPSE_MYCTU_RecName__Full_Phthiocerol_synthesis_polyketide_synthase_type_I

AIAVVGMAGRFPGA-KDVSAFWSNLRRGKES-IVTLSEQE-LRDAGVSDKTLA-DP--AYVR-RAPLLD----
GIDEFDAGFFGFPPLAAQVLDPQHRLFLQCAWHALED---AGAD---PARFDGS-IGVYGTSSPSGYLLHNLLSHR----DPNAVLAEGLNFDQ-F--
SLLATRISHAFNLRGPSIAVQTACSSSLVAVHLACLSLLSGECDMALAGGSSLCIPHRVGYF--TSPG-----SMV-SAVGHCRPFDVR---A-DGTVFGSG-
VGLVVLKP--LAAAIDAGDR-IHAVIRGSAINNDGSAKMG----YAAPNPAAQADVIAEAHAVSG---ID-SST----VSYVECHGTGTPLGDPIEIQGLRA-AF----
EVSRSAPCVLGSVKSNIGHLEVAAGIAGLIKTILCLKNKALPATLHYT-SP-NPELR--LDQSPFVVQSKYGPWE--CD---G-------
VRRAGVSSFGVGGTNAHVVLEEAPAAHAEPAGPQVILLSAQTAAALGESRTALAAAL--ETQDPRLSDVAYTLARRRK

>sp|P9WQE2.1|PPSD_MYCTO_RecName__Full_Phthiocerol_synthesis_polyketide_synthase_type_I

PVAVVGIGCRFPGNVTGPESFWQLLADGVDT-IEQVPPDR-WDADAFYDPDPS-ASGRMTTK-WGGFVS----
DVDAFDADFFGITPREAVAMDPQHRMLLEVAWEALEH---AGIP---PDSLSGTRTGVMMGLSSWDY-----TIVNIE--RRADIDAYLSTGTP-H--
CAAVGRIAYLLGLRGPAVAVDTACSSSLVAIHLACQSLRLRETDVALAGGVQLTLSPFTAIA--LSKW-----SAL-SPTGRCNSFDAN---A-DGFVRGEG-
CGVVVLKR--LADAVRDQDR-VLAVVRGSATNSDGRSN-----GMTAPNALAQRDVITSALKLAD---VT-PDS----VNYVETHGTGTVLGDPIEFESLAA-
TYGLGKGQ-GESPCALGSVKTNIGHLEAAAGVAGFIKAVLAVQRGHIPRNLHFT-RW-NPAID--ASATRLFVPTESAPWPAAAG----------
PRRAAVSSFGLSGTNAHVVVEQAPDAAGGMPYVSALNVSGKTAARVASAAAVLADWMSGPGAAAPLADVAHTLNRHRA

>sp|P9WQE3.1|PPSD_MYCTU_RecName__Full_Phthiocerol_synthesis_polyketide_synthase_type_I

PVAVVGIGCRFPGNVTGPESFWQLLADGVDT-IEQVPPDR-WDADAFYDPDPS-ASGRMTTK-WGGFVS----
DVDAFDADFFGITPREAVAMDPQHRMLLEVAWEALEH---AGIP---PDSLSGTRTGVMMGLSSWDY-----TIVNIE--RRADIDAYLSTGTP-H--
CAAVGRIAYLLGLRGPAVAVDTACSSSLVAIHLACQSLRLRETDVALAGGVQLTLSPFTAIA--LSKW-----SAL-SPTGRCNSFDAN---A-DGFVRGEG-
CGVVVLKR--LADAVRDQDR-VLAVVRGSATNSDGRSN-----GMTAPNALAQRDVITSALKLAD---VT-PDS----VNYVETHGTGTVLGDPIEFESLAA-
TYGLGKGQ-GESPCALGSVKTNIGHLEAAAGVAGFIKAVLAVQRGHIPRNLHFT-RW-NPAID--ASATRLFVPTESAPWPAAAG----------
PRRAAVSSFGLSGTNAHVVVEQAPDAAGGMPYVSALNVSGKTAARVASAAAVLADWMSGPGAAAPLADVAHTLNRHRA

>sp|P9WQE4.1|PPSB_MYCTO_RecName__Full_Phthiocerol_synthesis_polyketide_synthase_type_I

PVAVVGIGCRFPGDVDGPESFWDFLVAGRNA-ISTVPADR-WDAEAFYHPDPL-TPGRMTTK-WGGFVP----
DVAGFDAEFFGITPREAAAMDPQQRMLLEVAWEALEH---AGIP---PDSLGGTRTAVMMGVYFNEY-----QSMLAA--SPQNVDAYSGTGNA-H--
SITVGRISYLLGLRGPAVAVDTACSSSLVAVHLACQSLRLRETDLALAGGVSITLRPETQIA--ISAW-----GLL-SPQGRCAAFDAA---A-DGFVRGEG-
AGVVVLKR--LTDAVRDGDQ-VLAVVRGSAVNQDGRSN-----GVTAPNTAAQCDVIADALRSGD---VA-PDS----VNYVEAHGTGTVLGDPIEFEALAA-TY----
GH-GGDACALGAVKTNIGHLEAAAGIAGFIKATLAVQRATIPPNLHFS-QW-NPAID--AASTRFFVPTQNSPWPTAEG----------
PRRAAVSSFGLGGTNAHVIIEQGSEEGGEDTGVSTLVVTGKTAQRMAATAQVLADWMEGPGAEVAVADVAHTVNHHRA

>sp|P9WQE5.1|PPSB_MYCTU_RecName__Full_Phthiocerol_synthesis_polyketide_synthase_type_I

PVAVVGIGCRFPGDVDGPESFWDFLVAGRNA-ISTVPADR-WDAEAFYHPDPL-TPGRMTTK-WGGFVP----
DVAGFDAEFFGITPREAAAMDPQQRMLLEVAWEALEH---AGIP---PDSLGGTRTAVMMGVYFNEY-----QSMLAA--SPQNVDAYSGTGNA-H--
SITVGRISYLLGLRGPAVAVDTACSSSLVAVHLACQSLRLRETDLALAGGVSITLRPETQIA--ISAW-----GLL-SPQGRCAAFDAA---A-DGFVRGEG-
AGVVVLKR--LTDAVRDGDQ-VLAVVRGSAVNQDGRSN-----GVTAPNTAAQCDVIADALRSGD---VA-PDS----VNYVEAHGTGTVLGDPIEFEALAA-TY----
GH-GGDACALGAVKTNIGHLEAAAGIAGFIKATLAVQRATIPPNLHFS-QW-NPAID--AASTRFFVPTQNSPWPTAEG----------
PRRAAVSSFGLGGTNAHVIIEQGSEEGGEDTGVSTLVVTGKTAQRMAATAQVLADWMEGPGAEVAVADVAHTVNHHRA

>sp|P9WQE6.1|PPSA_MYCTO_RecName__Full_Phthiocerol_synthesis_polyketide_synthase_type_I

PIAVVGMGCRFPGGISCPEALWDFLCERRSS-ISQVPPQR-WQPFEGGPPEVAAALAR-TTR-WGSFLP----
DIDAFDAEFFEISPSEADKMDPQQRLLLEVAWEALEH---AGIP---PGTLRRSATGVFAGACLSEY-----GAMASA--DLSQVDGWSNSGGA-M--
SIIANRLSYFLDLRGPSVAVDTACSSSLVAIHLACQSLRTQDCHLAIAAGVNLLLSPAVFRG--FDQV-----GAL-SPTGQCRAFDAT---A-DGFVRGEG-
AGVVVLKR--LTDAQRDGDR-VLAVICGSAVNQDGRSN-----GLMAPNPAAQMAVLRAAYTNAG---MQ-PSE----VDYVEAHGTGTLLGDPIEARALGT-VL----
GRPEDSPLLIGSVKTNLGHTEAAAGIAGFIKTVLAVQHGQIPPNQHFE-TA-NPHIP--FTDLRMKVVDTQTEWP--AT---GH------
PRRAGVSSFGFGGTNAHVVIEQGQEGQGLSPAVSTLVVAGKTMQRVSATAGMLADWM-EGPGAVALADVAHTLNHHRS

>sp|P9WQE7.1|PPSA_MYCTU_RecName__Full_Phthiocerol_synthesis_polyketide_synthase_type_I

PIAVVGMGCRFPGGISCPEALWDFLCERRSS-ISQVPPQR-WQPFEGGPPEVAAALAR-TTR-WGSFLP----
DIDAFDAEFFEISPSEADKMDPQQRLLLEVAWEALEH---AGIP---PGTLRRSATGVFAGACLSEY-----GAMASA--DLSQVDGWSNSGGA-M--
SIIANRLSYFLDLRGPSVAVDTACSSSLVAIHLACQSLRTQDCHLAIAAGVNLLLSPAVFRG--FDQV-----GAL-SPTGQCRAFDAT---A-DGFVRGEG-
AGVVVLKR--LTDAQRDGDR-VLAVICGSAVNQDGRSN-----GLMAPNPAAQMAVLRAAYTNAG---MQ-PSE----VDYVEAHGTGTLLGDPIEARALGT-VL----
GRPEDSPLLIGSVKTNLGHTEAAAGIAGFIKTVLAVQHGQIPPNQHFE-TA-NPHIP--FTDLRMKVVDTQTEWP--AT---GH------
PRRAGVSSFGFGGTNAHVVIEQGQEGQGLSPAVSTLVVAGKTMQRVSATAGMLADWM-EGPGAVALADVAHTLNHHRS

>sp|Q02059.2|KASA_STRCO_RecName__Full_Actinorhodin_polyketide_putative_beta-ketoacyl_synthase_1

RVVITGVGVRAPGG-NGTRQFWELLTSGRTA----------TRRISFFDPSPYRSQVA---------------AEADFDPVAEGFGPRELDRMDRASQFAVACAREAFAA---
SGLD---PDTLDPARVGVSLGSAVAYLLLSDSGRDWEV--DAAWLSRHMFDYLV-P--
SVMPAEVAWAVGAEGPVTMVSTGCTSGLDSVGNAVRAIEEGSADVMFAGAADTPITPIVVAC--FDAI-RATTARNDDPEHASRPFDGT---R-DGFVLAEG-
AAMFVLED--YDSALARGAR-IHAEISGYATRCNAYHMTG-----LKADGREMAETIRVALDESR---TD-ATD----IDYINAHGSGTRQNDRHETAAYKRALG------
EHARRTPVSSIKSMVGHSLGAIGSLEIAACVLALEHGVVPPTANLR--

>sp|Q02062.1|KASB_STRCO_RecName__Full_Actinorhodin_polyketide_putative_beta-ketoacyl_synthase_2

SVLITGVGVVAPNG-LGLAPYWSAVLDGRHG---LGPVTR-FDVSRYPATLAG--------------------QIDDFHAP-DHIPGRLLPQTDPSTRLALTAADWALQD---
AKAD---PESLTDYDMGVVTANACGDFTHREFRKLWSE--GPKSVSVYESFAWF-Y--AVNTGQISIRHGMRGPSSALVAEQAGGLDALGHARRTIRRG-
TPLVVSGGVDSALDPWGWVS-QIASG---RISTATDPDRAYLPFDER---A-AGYVPGEG-GAILVLED--SAAAEARGRHDAYGELAGCASTFDPAPGSG--------
RPAGLERAIRLALNDAG---TG-PED----VDVVFADGAGVPELDAAEARAIGR-VF-------
GREGVPVTVPKTTTGRLYSGGGPLDVVTALMSLREGVIAPTAGVT---

>sp|Q02251.2|MCAS_MYCBO_RecName__Full_Mycocerosic_acid_synthase

PVAVIGMGCRLPGGINSPDKLWESLLRGDDL-VTEIPPDR-WDADDYYDPEPG-VPGR-SVSRWGGFLD----
DVAGFDAEFFGISEREATSIDPQQRLLLETSWEAIEH---AGLD---PASLAGSSTAVFTGLTHEDY-----LVLTTT--AGGLASPYVVTGLN-N--
SVASGRIAHTLGLHGPAMTFDTACSSGLMAVHLACRSLHDGEADLALAGGCAVLLEPHACVA--ASAQ-----GML-SSTGRCHSFDAD---A-DGFVRSEG-
CAMVLLKR--LPDALRDGNR-IFAVVRGTATNQDGRTET-----LTMPSEDAQVAVYRAALAAAG---VQ-PET----VGVVEAHGTGTPIGDPIEYRSLAR-VY------
GAGTPCALGSAKSNMGHSTASAGTVGLIKAILSLRHGVVPPLLHFN----RLPDELSDVETGLFVPQAVTPWPNGND---------
HTPKRVAVSSFGMSGTNVHAIVEEAPAPGDAEVGPRLFMLSSTSSDALRQTARQLATWVEEHQDCVAASDLAYTLARGRA

>sp|Q03131.1|ERYA1_SACER_RecName__Full_6-deoxyerythronolide-B_synthase

----
--AVLRGTAVNSDGA--SN---GLSAPNGRAQVRVIQQALAESG---LG-PAD----
IDAVEAHGTGTRLGDPIEARALFE-AY----GR--EQPLHLGSVKSNLGHTQAAAGVAGVIKMVLAMRAGTLPRTLHAS-ER-SKEID--WSSGAISLLDEPEPWP--
A-----GAR----PRRAGVSSFGISGTNAHAIIEEAPQ-------VAPWVLSASSAEGLRAQAARLAAHL--REHP-DPRDIAYSLATGRA

>sp|Q03132.3|ERYA2_SACER_RecName__Full_6-deoxyerythronolide-B_synthase

PIAIVGIGCRFPGGIGSPEQLWRVLAEGANL-TTGFPADRGWDIGRLYHPDPD-NPGTSYVD-KGGFLT----
DAADFDPGFFGITPREALAMDPQQRLMLETAWEAVER---AGID---PDALRGTDTGVFVGMNGQSY-----MQLLAG--EAERVDGYQGLGNS-A--
SVLSGRIAYTFGWEGPALTVDTACSSSLVGIHLAMQALRRGECSLALAGGVTVMSDPYTFVD--FSTQ-----RGL-ASDGRCKAFSAR---A-DGFALSEG-
VAALVLEP--LSRARANGHQ-VLAVLRGSAVNQDGASN-----GLAAPNGPSQERVIRQALAASG---VP-AAD----VDVVEAHGTGTELGDPIEAGALIA-TY----
GQDRDRPLRLGSVKTNIGHTQAAAGAAGVIKVVLAMRHGMLPRSLHAD-EL-SPHID--WESGAVEVLREEVPWP--AG---ER------
PRRAGVSSFGVSGTNAHVIVEEAPAAARTERGPLPFVLSGRSEAVVAAQARALAEHL-RDTPELGLTDAAWTLATGRA

>sp|Q03133.4|ERYA3_SACER_RecName__Full_6-deoxyerythronolide-B_synthase

----
--AVLAGSAVNQDGA--SN---GLTAPSGPAQQRVIRRALENAG---VR-AGD----
VDYVEAHGTGTRLGDPIEVHALLS-TY----GADPDDPLWIGSVKSNIGHTQAAAGVAGVMKAVLALRHGEMPRTLHFD-EP-SPQIE--
WDLGAVSVVSQARSWP--A-----GER----PRRAGVSSFGISGTNAHVIVEEAPE--------VPLVLSGRDEQAMRAQAGRLADHL--APEP-SLRDTGFTLATRAS

>sp|Q05470.3|PKSL_BACSU_RecName__Full_Polyketide_synthase_PksL_Short_PKS

EIAIIGISGRYPQA-DNIDELWEKLRDGRDC-ITEIPADR-WDHSLYYDEDKD-KPGKTYSK-WGGFMK----
DVDKFDPQFFHISPREAKLMDPQERLFLQCVYETMED---AGYT---REHLGRKSVGVYVGVMYEEY-----QLYGAQ--EQVRGRSLALTGNPSS----
IANRVSYYFDFHGPSIALDTMCSSSLTAIHLACQSLQRGECEAAFAGGVNVSIHPNKYLM--LGQN-----KFM-SSKGRCESFGQG---G-DGYVPGEG-
VGAVLLKP--LSKAVEDGDH-IYGIIKGTAINHGGKTN-----GYSVPNPNAQADVIKKAFVEAK---VD-PRT----VSYIEAHGTGTSLGDPIEITGLSK-VFTQETDD-
-KQFCAIGSAKSNIGHCESAAGIAGVTKVLLQMKYRQLAPSLHSN-VL-NPNID--FLNSPFKVQQELEEWK--
RPIISVNGKDIELPRIAGVSSFGAGGVNAHILIEEYAPRLTKQHRSALFVLSAKKEKQLKAYAEAMKDFV-TSNEDIDLEDMAYTLQTGRE

>sp|Q07017.1|OL56_STRAT_RecName__Full_Oleandomycin_polyketide_synthase__modules_5_and_6

--AVVRGSAVNQDGA--SN---GLTAPNGPSQQRVIRAALADAG---LA-PAD----
VDVVEAHGTGTRLGDPIEAQALLA-TY----GQAGGRPVWLGSVKSNIGHTQAAAGVAGVMKMVLALGRGVVPKTLHVD-EP-SPHVD--
WSAGAVELLTEERPW---EP---EAER----LRRAGISAFGVSGTNAHVIVEEAP-AAGD--LVVPWVVSGRDARALRAQAARLAAHV--S---
VSAVDVGWSLVATRS

>AAR90242.1_polyketide_synthase__Botrytis_cinerea_1

RVAIVDYQ-PITGHPLDKTQFWQTLEQGEDA-LREHSAAH-VNAEAFVRTTQQ-KLSTYIHR-TMSFPAHSPSDVALKKP----
MMPAKKQRLDVTQLYALNSCFERVAIIIASNLS---LSADRLQMRALWSGLPGSEG--AI-PLPELP--SINHWSWYGACGIG------
TAQLLAQYFGISADCYAVEAACASSLAAVHDAVRALQAGRYDAVIVGGIETATLERDLV---LCSA-----QMM-LSVSRIRPFSQG---A-DGFTPGDG-
GGFVMLTH-------HPVPR-AIATIEAISGSCDSY-------SMTAPDPLGQALAIKKTLSLTA---ID-AQT----VQYLEAHGTGTELGDRSEVMSLKY-SY----
HRDKHSPLYIGSAKYNFGHCFAGAGALSLCKVLSAFEHERIPPTPVSE--L-NVDLP--LGDIPAEVPQQAIPWR--LS---EDG-----
QRKAAINAFGTGGINYHLVIRQSS--

>sp|Q7TXK8.1|MSL7_MYCBO_RecName__Full_Phenolphthiocerol_synthesis_polyketide_synthase_type_I

PVAVVGIGCRFPGGVDGPDGLWDVVSAGRDV-VSEFPTDRGWDVEGLYDPDPD-AEGKTYTR-WGAFLD----
DATGFDAGFFGIAPSEVLAMDPQQRLMLEVSWEALEH---AGID---PLSLRGSATGVYTGIFAASY--------GNR--DTGGLQGYGLTGTS-I--
SVASGRVSYVLGLQGPAVSVDTACSSSLVAIHWAMSSLRSGECDLALAGGVTVMGLPSIFVG--FSRQ-----RGL-AADGRCKAFAAA---A-DGTGWGEG-
AGVVVLER--LSDARRLGHS-VLAVVRGSAVNQDGASN-----GLTAPNGLAQQRVIQAALANAG---LS-AAD----VDVVEAHGTATTLGDPIEAQALLS-TY----
GQPAEQPLWVGSIKSNMGHTQAAAGVAGVIKMVQAMRHGVMPATLHVD-EP-SPRVD--WTSGAVSVLTEAREWS--VD---GR------
PRRAAVSSFGISGTNAHLILEEAPVTGGPRPSMVPWVISARSAEALTAQAGRLMAHV-QANPGLDPIDVGCSLASRSV

>sp|Q7TXL6.1|PPSE_MYCBO_RecName__Full_Phthiocerol/phenolphthiocerol_synthesis_polyketide_synthase_type_I

AIAVVGMAGRFPGA-KDVSAFWSNLRRGKES-IVTLSEQE-LRDAGVSDKTLA-DP--AYVR-RAPLLD----
GIDEFDAGFFGFPPLAAQVLDPQHRLFLQCAWHALED---AGAD---PARFDGS-IGVYGTSSPSGYLLHNLLSHR----DPNAVLAEGLNFDQ-F--
SLLATRISHAFNLRGPSIAVQTACSSSLVAVHLACLSLLSGECDMALAGGSSLCIPHRVGYF--TSPG-----SMV-SAVGHCRPFDVR---A-DGTVFGSG-
VGLVVLKP--LAAAIDAGDR-IHAVIRGSAINNDGSAKMG----YAAPNPAAQADVIAEAHAVSG---ID-SST----VSYVECHGTGTPLGDPIEIQGLRA-AF----
EVSRSAPCVLGSVKSNIGHLEVAAGIAGLIKTILCLKNKALPATLHYT-SP-NPELR--LDQSPFVVQSKYGPWE--CD---G-------
VRRAGVSSFGVGGTNAHVVLEEAPAAHAEPAGPQVILLSAQTAAALGESRTALAAAL--ETQDPRLSDVAYTLARRRK

>sp|Q7TXL7.1|PPSD_MYCBO_RecName__Full_Phthiocerol/phenolphthiocerol_synthesis_polyketide_synthase_type_I

PVAVVGIGCRFPGNVTGPESFWQLLADGVDT-IEQVPPDR-WDADAFYDPDPS-ASGRMTTK-WGGFVS----
DVDAFDADFFGITPREAVAMDPQHRILLEVAWEALEH---AGIP---PDSLSGTRTGVMMGLSSWDY-----TIVNIE--RRADIDAYLSTGTP-H--
CAAVGRIAYLLGLRGPAVAVDTACSSSLVAIHLACQSLRLRETDVALAGGVQLTLSPFTAIA--LSKW-----SAL-SPTGRCNSFDAN---A-DGFVRGEG-
CGVVVLKR--LADAVRDQDR-VLAVVRGSATNSDGRSN-----GMTAPNALAQRDVITSALKLAD---VT-PDS----VNYVETHGTGTVLGDPIEFESLAA-
TYGLGKGQ-GESPCALGSVKTNIGHLEAAAGVAGFIKAVLAVQRGHIPRNLHFT-RW-NPAID--ASATRLFVPTESAPWPAAAG-----------
PRRAAVSSFGLSGTNAHVVVEQAPDAAGGMPYVSALNVSGKTAARVASAAAVLADWMSGPGAAAPLADVAHTLNRHRA

>sp|Q7TXL8.1|PPSC_MYCBO_RecName__Full_Phthiocerol/phenolphthiocerol_synthesis_polyketide_synthase_type_I

PIAVIGMGCRFPGGVNNPEQFWDLLCAGRSG-IVRVPAQR-WDADAYYCDDHT-VPGTICST-EGGFLTSW--
QPDEFDAEFFSISPREAAAMDPQQRLLIEVAWEALED---AGVP---QHTIRGTQTSVFVGVTAYDYMLTLAGRLR-----PVDLDAYIPTGNS-A--
NFAAGRLAYILGARGPAVVIDTACSSSLVAVHLACQSLRGRESDMALVGGTNLLLSPGPSIA--CSRW-----GML-SPEGRCKTFDAS---A-DGYVRGEG-
AAVVVLKR--LDDAVRDGNR-ILAVVRGSAVNQDGASS-----GVTVPNGPAQQALLAKALTSSK---LT-AAD----IDYVEAHGTGTPLGDPIELDSLSK-
VFSDRAGS---DQLVIGSVKTNLGHLEAAAGVAGLMKAVLAVHNGYIPRHLNFH-QL-TPHAS--EAASRLRIAADGIDWP--TT---GR------
PRRAGVSSFGVSGTNAHVVIEQAPDQRGPVPAVSTLVVFGKTAPRVAATASVLADWL--DGPGVPLADVAHTLNHHRA

>sp|Q7TXL9.1|PPSB_MYCBO_RecName__Full_Phthiocerol/phenolphthiocerol_synthesis_polyketide_synthase_type_I

PVAVVGIGCRFPGDVDGPESFWDFLVAGRNA-ISTVPADR-WDAEAFYHPDPL-TPGRMTTK-WGGFVP----
DVAGFDAEFFGITPREAAAMDPQQRMLLEVAWEALEH---AGIP---PDSLGGTRTAVMMGVYFNEY-----QSMLAA--SPQNVDAYSGTGNA-H--

SITVGRISYLLGLRGPAVAVDTACSSSLVAVHLACQSLRLRETDLALAGGVSITLRPETQIA--ISAW-----GLL-SPQGRCAAFDAA---A-DGFVRGEG-
AGVVVLKR--LTDAVRDGDQ-VLAVVRGSAVNQDGRSN-----GVTAPNTAAQCDVIADALRSGD---VA-PDS----VNYVEAHGTGTVLGDPIEFEALAA-TY----
GH-GGDACALGAVKTNIGHLEAAAGIAGFIKATLAVQRATIPPNLHFS-QW-NPAID--AASTRFFVPTQNSPWPTAEG-----------
PRRAAVSSFGLGGTNAHVIIEQGSEEGGEDTGVSTLVVTGKTAQRMAATAQVLADWMEGPGAEVAVADVAHTVNHHRA

>sp|Q7TXM0.1|PPSA_MYCBO_RecName__Full_Phthiocerol/phenolphthiocerol_synthesis_polyketide_synthase_type_I

PIAVVGMGCRFPGGISCPEALWDFLCERRSS-ISQVPPQR-WQPFEGGPPEVAAALAR-TTR-WGSFLP----
DIDAFDAEFFEISPSEADKMDPQQRLLLEVAWEALEH---AGIP---PGTLRRSATGVFAGACLSEY-----GAMASA--DLSQVDGWSNSGGA-M--
SIIANRLSYFLDLRGPSVAVDTACSSSLVAIHLACQSLRTQDCHLAIAAGVNLLLSPAVFRG--FDQV-----GAL-SPTGQCRAFDAT---A-DGFVRGEG-
AGVVVLKR--LTDAQRDGDR-VLAVICGSAVTQDGRSN-----GLMAPNPAAQMAVLRAAYTNAG---MQ-PSE----VDYVEAHGTGTLLGDPIEARALGT-VL----
GRPEDSPLLIGSVKTNLGHTEAAAGIAGFIKTVLAVQHGQIPPNQHFE-TA-NPHIP--FTDLRMKVVDTQTEWP--AT---GH------
PRRAGVSSFGFGGTNAHVVIEQGQEGQGLSPAVSTLVVAGKTMQRVSATAGMLADWM-EGPGAVALADVAHTLNHHRS

>sp|Q9ZGI2.1|PIKA4_STRVZ_Full_Pikromycin_polyketide_synthaS

PMAIVGMSCRFAGGIRSPEDLWDAVAAGKDL-VSEVPEERGWDIDSLYDPVPG-RKGTTYVR-NAAFLD----
DAAGFDAAFFGISPREALAMDPQQRQLLEASWEVFER---AGID---PASVRGTDVGVYVGCGYQDY-----APDIRV--APEGTGGYVVTGNS-S--
AVASGRIAYSLGLEGPAVTVDTACSSSLVALHLALKGLRNGDCSTALVGGVAVLATPGAFIE--FSSQ-----QAM-AADGRTKGFASA---A-DGLAWGEG-
VAVLLLER--LSDARRKGHR-VLAVVRGSAINQDGASN-----GLTAPHGPSQQHLIRQALADAR---LT-SSD----VDVVEGHGTGTRLGDPIEAQALLA-TY----
GQAPGQPLRLGTLKSNIGHTQAASGVAGVIKMVQALRHGVLPKTLHVD-EP-TDQVD--WSAGSVELLTEAVDWP--ER---PGR-----
LRRAGVSAFGVGGTNAHVVLEEAPAEPPAGGGVVPWPVSAKTSAALDAQIGQLAAYA-EDRTDVDPAVAARALVDSRT

>sp|Q9ZGI3.1|PIKA3_STRVZ_Full_Pikromycin_polyketide_synthS

PVAIVGMACRLPGGVASPEDLWQLVAGDGDA-ISEFPQDRGWDVEGLYDPDPD-ASGRTYCR-SGGFLH----
DAGEFDADFFGISPREALAMDPQQRLSLTTAWEAIES---AGID---PTALKGSGLGVFVGGWHTGY---TSGQTTAV--QSPELEGHLVSGAA-L--
GFLSGRIAYVLGTDGPALTVDTACSSSLVALHLAVQALRKGECDMALAGGVTVMPNADLFVQ--FSRQ-----RGL-AADGRSKAFATS---A-DGFGPAEG-
AGVLLVER--LSDARRNGHR-ILAVVRGSAVNQDGASN-----GLTAPHGPSQQRVIRRALADAR---LA-PGD----VDVVEAHGTGTRLGDPIEAQALIA-TY----
GQSSEQPLRLGALKSNIGHTQAAAGVAGVIKMVQAMRHGLLPKTLHVD-EP-SDQID--WSAGTVELLTEAVDWP--EK---QDGG----
LRRAAVSSFGISGTNAHVVLEEAPAEPPAGGGVVPWPVSAKTPAALDAQIGQLAAYA-DGRTDVDPAVAARALVDSRT

>sp|Q9ZGI4.1|PIKA2_STRVZ_Pikromycin_polyketideS

PVAIVGMACRLPGGVASPEDLWRLVAGGEDA-ISGFPQDRGWDVEGLYDPDPD-ASGRTYCR-AGGFLD----
EAGEFDADFFGISPREALAMDPQQRLLLETSWEAVED---AGID---PTSLQGQQVGVFAGTNGPHY-----EPLLRN--TAEDLEGYVGTGNA-A--
SIMSGRVSYTLGLEGPAVTVDTACSSSLVALHLAVQALRKGECGLALAGGVTVMSTPTTFVE--FSRQ-----RGL-AEDGRSKAFAAS---A-DGFGPAEG-
VGMLLVER--LSDARRNGHR-VLAVVRGSAVNQDGASN-----GLTAPNGPSQQRVIRRALADAR---LT-TAD----VDVVEAHGTGTRLGDPIEAQALIA-TY----
GQDTEQPLRLGSLKSNIGHTQAAAGVSGIIKMVQAMRHGVLPKTLHVD-RP-SDQID--WSAGTVELLTEAMDWP--RK---QEGG----
LRRAAVSSFGISGTNAHIVLEEAPVDEPSVGGVVPWLVSAKTPAALDAQIGRLAAFA-SQGRTADPGAVARVLAGGRA

>sp|Q9ZGI5.1|PIKA1_STRVZFull_Pikromycin_polyketide_S

PVAIVGMACRLPGGVASPEDLWRLVAGGGDA-ISEFPQDRGWDVEGLYHPDPE-HPGTSYVR-QGGFIE----
NVAGFDAAFFGISPREALAMDPQQRLLLETSWEAVED---AGID---PTSLRGRQVGVFTGAMTHEY-----GPSLRD--GGEGLDGYLLTGNT-A--
SVMSGRVSYTLGLEGPALTVDTACSSSLVALHLAVQALRKGEVDMALAGGVAVMPTPGMFVE--FSRQ-----RGL-AGDGRSKAFAAS---A-DGTSWSEG-
VGVLLVER--LSDARRNGHQ-VLAVVRGSALNQDGASN-----GLTAPNGPSQQRVIRRALADAR---LT-TSD----VDVVEAHGTGTRLGDPIEAQALIA-TY----
GQDDEQPLRLGSLKSNIGHTQAAAGVSGVIKMVQAMRHGLLPKTLHVD-EP-SDQID--WSAGAVELLTEAVDWP--EK---QDGG----
LRRAAVSSFGISGTNAHVVLEEAPVGSAVGGGVTPWVVSAKSAAALDAQIERLAAFA-SRDRTVDAGAVAHVLADGRA

>TRINITY_DN10243_c0_g1_i2_

-------------------HINTLEQSKTL---RFLNKITMKIRFSFEIINCKILN---IGID---PNSLSGTNTGIFIGSCYDEF----------V--
NAYSNDHTKFVGTK----QFFAEMVAHKFNLRGPSKLIDTACASSFSALHEAIVSLRSNQCDRAIVMGLNICLRATTQLT--FTHM-----NMI-SPDGHCKCLDED--
-A-NGTAKGEA-LVVMILQK--KSEAKR-----ITATVVHTKTNCDGTTELG----ITYPSTEFQRDLIRDTTSEIG---ID-PLN----
VKTCEAHCTGTQAGDPTEMRAICE-SM----CIERKDPLLIGALKSNIGHAEGASGFCSLTKVILSFENKCIPANMHMT-VP-NSRIE-GLVSGILKPVIKNTQFE-----
----------GELACLNCFGFGGVNVHVIVKHNDKHQIVNQIPRLVQMCGRNEELLNQTFTSLLRNP--EKLTRDYLSLINDISKTSP

>TRINITY_GG_3134_c21_g1__TRINITY_GG_3134_c21_g1_i2

DIVISGVGGRFPES-DSIDEFRDNLYNNVDM---ITADDRRWPTPLYGMSP---RMGKLK-------------EIDKLDSTFFGIVPKVADEVDPQARILLETTYEAIFD---
AGIN---PQTLRGSNTGVYVGYTSVE------VQEDLQ--SSKVESLLWFQGNS-K--
AFYANRVSFVFDFHGPSMSIDVACAASMVALDIAVTDIRLGKCDQAIVAGVSINLQPFTNHI--YQVN-----NIG-SKEGMSRVWDQD---A-NGYVRGET-
VASLFLQR--KSDSKR-----IYATILHSRTNIDGYKISG----MFFPSSESQYDLMVTTYTEAG---IN-PHD----VNYFEGHGTGTKVGDPQEARAILE-AY----
CKDRKSVLPVGLLKSNIGHGEGASGVASLAKLLIVYENKKIPANLNLK----TLKTEIAEMVPPLVPVTENTDYE---------------
PGIAGVNSFGIGGVNCHALLKPNKK---------LVNICGRTEEAVNYIFEFIEKNP--QKVTRDFLALIGEIVNPVS

>TRINITY_GG_3293_c9_g1__TRINITY_GG_3293_c9_g1_i10

---NQRMGKLK-------------EIDKFDGVFFGLMSEIADAVDPHSRILLETTYEAICD---AGVN---
PQTLRATKTGVYIGYSSIE------IQLDSR--TSMKDSATWAPGSS-K--
AMYSNRISFFFDFKGPSLVIDTACSSSMVAFDVAVTDLRLGKCDQAIVGGTNICLQPFTNHI--YQTI-----KFN-SPDGISRVWDEE---S-VGFVRGET-
VACLFLQR--KSDAKR-----IYATVLHSRTNIDGYKKMG----PFFPSSECQRDLMVTTYKEAN---VD-PLE----VTYFEAHGTGTKAGDLQEARAIFN-AY----
CDGREGTLPIGLLKSNIGHGEGACGIASITKVIISYENECIAPNLNLK----QLKTEISVMCPPLFPVTENLTYI---------------
PGIAGVNSFGVGGVNGHALLQPNYE---------LINICARNEDSLKSMFNFIESHS--NKITQHFLALITDVMKTKP

>TRINITY_GG_3293_c9_g1__TRINITY_GG_3293_c9_g1_i4

EIVISGMSGRFPES-DSTDEFAQNLYNKVDM---ITEDDRRWPTDLYS---MNQRMGKLK-------------EIDKFDGVFFGLMSEIADAVDPHSRILLETTYEAICD---
AGVN---PQTLRATKTGVYIGYSSIE------IQLDSR--TSMKDSATWAPGSS-K--

AMYSNRISFFFDFKGPSLVIDTACSSSMVAFDVAVTDLRLGKCDQAIVGGTNICLQPFTNHI--YQTI-----KFN-SPDGISRVWDEE---S-VGFVRGET-
VACLFLQR--KSDAKR-----IYATVLHSRTNIDGYKKMG----PFFPSSECQRDLMVTTYKEAN---VD-PLE----VTYFEAHGTGTKAGDLQEARAIFN-AY----
CDGREGTLPIGLLKSNIGHGEGACGIASITKVIISYENECIAPNLNLK-QL-KTEIS--VMCPPLFPVTENLTYI--------------
PGIAGVNSFGVGGVNGHALLQPNYE---------LINICARNEDSLKSMFNFIESHS--NKITQHFLALITDVMKTKP

>TRINITY_GG_3293_c9_g1__TRINITY_GG_3293_c9_g1_i8

--NQRMGKLK-------------EIDKFDGVFFGLMSEIADAVDPHSRILLETTYEAICD---AGVN---
PQTLRATKTGVYIGYSSIE------IQLDSR--TSMKDSATWAPGSS-K--
AMYSNRISFFFDFKGPSLVIDTACSSSMVAFDVAVTDLRLGKCDQAIVGGTNICLQPFTNHI--YQTI-----KFN-SPDGISRVWDEE---S-VGFVRGET-
VACLFLQR--KSDAKR-----IYATVLHSRTNIDGYKKMG----PFFPSSECQRDLMVTTYKEAN---VD-PLE----VTYFEAHGTGTKAGDLQEARAIFN-AY----
CDGREGTLPIGLLKSNIGHGEGACGIASITKVIISYENECIAPNLNLK----QLKTEISVMCPPLFPVTENLTYI--------------
PGIAGVNSFGVGGVNGHALLQPNYE---------LINICARNEDSLKSMFNFIESHS--NKITQHFLALITDVMKTKP

>TRINITY_GG_4863_c25_g1__TRINITY_GG_4863_c25_g1_i2

DIVISGVAGRFPES-DNIDELKENLYNNVDM---ITEDERRWPLGLYGLPTRS-GKIK---------------DLSKFDAQFFGVHGKQANLMDPQARLLLELTYEAIAD---
AGIN---PQTLRGTRTGVYIGACVSEV-----EEGLAM--DISKVSGYALTGCS-R--
SMFANRVSYTFDFHGPSYSMDTACSSSFLALQQAVLGLRTGQCDMAVVGGVNICLRPVSALQ--FHKL-----NML-AVDGKCKFLDAS---A-NGYVRSET-
CSVIFLQK--KSEAKR-----IYATVIHAKTNTDGYKDQG----ITYPSWQSQSALMNSTLREAG---IK-PTD----INFVEAHGTGTGAGDPTESQAIFD-VF----
CKDRKEPLLIGGVKSNLGHSEPASGLCSIAKVLITFENKCIPANLHFK-TP-NANIP-ALVSGAIKPVVENTPFK---------------
DGVVAINSFGFGGVNVHVLLKPHQKYRVIDNIPRLIPICGRTEESVNYILDFLEQNP--KKVTRDLLALLYDISQTNE

>TRINITY_GG_6264_c208_g1__TRINITY_GG_6264_c208_g1_i1

----------------------NTLEQSKYL---RFLNKITMKIRFSFEIINCKILN---IGID---PNSLSGTNTGIFIGSCYDEFV------------
NAYSNDHTKFVGYK----QFFAEMVAHKFNLRGPSKLIDTACASSFSALHEAIVSLRSNQCDRAIVMGLNICLRATTQLY--FYHM-----NMI-SPDGHCKCLDED-
--A-NGYAKGEA-LVVMILQK--KSEAKR-----IYATVVHTKTNCDGYTELG----ITYPSYEFQRDLIRDTYSEIG---ID-PLN----
VKYCEAHCTGTQAGDPTEMRAICESMC------ERKDPLLIGALKSNIGHAEGASGFCSLTKVILSFENKCIPANMHMT-VP-NSRIE-GLVSGILKPVIKNTQFE----
-----------GELACLNCFGFGGVNVHVIVKHNDKHQIVNQIPRLVQMCGRNEELLNQTFTSLLRNPEKLT--RDYLSLINDISKTSP

>TRINITY_GG_6449_c125_g1__TRINITY_GG_6449_c125_g1_i1

RVVVTGLGIVSPIG-VGVEKVWNNLISGRCG-ITEVIGSG-YES--------------IPCK-IAAYVPK---NDLCLEQSF---AKNELRTLSKSTIYALIAAEEALTD---
AKWK---PTKERDKETGVAVGMGMVDEVVESGTVLRDV--GFNKISPHFVTKVL-M--
NMPAGHISMRYGLTGPNHTVSTACTTGVHSIGDAFNFIQRGAADVMICGGTEAVISPLSIAS--FARIRALCTQFNDNPSKASRPFDSK---R-CGFVMGEG-
CGLVVLEE--LNHALNRKAK-IYAEIIGYGLSGDANHITA-----PREDGKGAFECMKASIDDAH---IR-LDD----VTHINSHATSTPLGDKAELIAIKQ-LF----GE-
HSKNIAITSTKGSTGHLLGAAGSVEAIFTILSCYNSLIPPTINLE-EPIDKELN--------IVANNPQEWN--TK---N-------RRIAITNSFGFGGTNASLVISNF--------------
----

>TRINITY_GG_6449_c125_g1__TRINITY_GG_6449_c125_g1_i2

RVVVTGLGIVSPIG-VGVEKVWNNLISGRCG-ITEVIGSG-YES--------------IPCK-IAAYVPK---NDLCLEQSF---AKNELRTLSKSTIYALIAAEEALTD---
AKWK---PTKERDKETGVAVGMGMVDEVVESGTVLRDV--GFNKISPHFVTKVL-M--
NMPAGHISMRYGLTGPNHTVSTACTTGVHSIGDAFNFIQRGAADVMICGGTEAVISPLSIAS--FARIRALCTQFNDNPSKASRPFDSK---R-CGFVMGEG-
CGLVVLEE--LNHALNRKAK-IYAEIIGYGLSGDANHITA-----PREDGKGAFECMKASIDDAH---IR-LDD----VTHINSHATSTPLGDKAELIAIKQ-LF----GE-
HSKNIAITSTKGSTGHLLGAAGSVEAIFTILSCYNSLIPPTINLE-EPIDKELN--------IVANNPQEWN--TK---N-------RRIAITNSFGFGGTNASLVISNF--------------
----

>TRINITY_GG_6602_c113_g1__TRINITY_GG_6602_c113_g1_i1

----------------------NLTQAPFSNGI--YQSI-----GLL-ASDGKCKCVWDKR---A-DGFVRSET-VGALFLQK--KSEAKR-----IYATLLHAKTNTDGFKAIG----
LFSPFWLRQRDLMIETYHEAG---VD-PND----IKFFESHGTGTNVGDPQEAKAIAD-AY----
CHDRKEPLLLGAVKSNLGHSEGSSGLCSVAKVIVAFENKCIPANLHFE-EP-KPEIE-CVVNKTLIPVMKNTPFE---------------
NGIVGVNSFGVGGVNAHALLKSNDKYKIADPIPRLVNVSGRTEAAVKHIFDFIENNP-RKAHRALLNDSMKTLVIKGS

>XP_002589799.1_hypothetical_protein_BRAFLDRAFT_90481__Branchiostoma_floridae_

EVAIVGLWCRFPSA-EGPDEFWRVLENGEDC-MREIPPER-WNQEAWYNPDRN-TPGKSFVE-RAGFVD----
DFKDFDNRLFNISDLEASRMDPQQRWMLECSYKALEN---AGIP---LKNVYNTDTGVFVGVMNYDY-------YWNSTLDMADVSSNTATGVS-I--
PVVANRVSYAFNLTGPSMTVDTACSSGMTALHLGCQAIRAGDCTMAICGGVNFILNPGMFVQ--LCRA-----GMV-CPDGRCKPFSAA---A-NGYARGEG-
CGVVIIKS--FRKEQLIRRLYEQHN---VD-PRH----IDYIEAHGTGTPVGDPVEARSLGKAIGQSRDSS--
EPPVLIGSVKSNFGHLESAAAMAGLIKVLLMMKHKKIAPTLHFN-EP-NPNID--FEGLKIKVCTEPITWSSRNKD----------
KMMACVNSFGFGGSNAHAILFSEPSELGRHEEPNIIAVSANGKESLQASMKDLISFLEKNQC-TALSQLAYTSSVKRT

>XP_002598380.1_hypothetical_protein_BRAFLDRAFT_96863__Branchiostoma_floridae_

KVAIVGIGCRYPGGVHTPADFWTMLAEGRDC-TIPPPDVR-FDTSFLWHPY-K-TPGKLYNR-CGGYLQC---
NVFEFDRQFFKIPPDEANHLDPQIQLLLEVTWEALEN---AGIP---PRSIRGSNTGVYVGVTSSEY--LT-MTCS----PYSNISQYTNSGTN-S--
CMVSNRISYEFDLHGPSFSVDTACSSSLYSIHLASEAIRKGDCSMAIAGGVNLMLLPITSVG--FCQA-----GML-SPDGKCKSFDAS---A-DGYCRSEG-
AGVVVLKP--LSRALADGDR-VYAVIRGGTLTNDGR--TP---GIANPSYDAQLDLVEKACAAAK---VH-PHD----IQYVEAHGTGTKVGDRTEANALGQ-IL----
GRKEDPPLYIGSVKSNFGHAEGAAGVAGIIKLALMISKGQIPRVVHFS-SP-NPDIH--FDALNIKVPSTLLQWE-------GKG-----
SRLAGCSSFGFGGANAHLILERPPTRGTERETKTIMLLSGNTKAALKEQVEHWISFL--SDTTDRFLQSLYTAANRG-

>XP_002599684.1_hypothetical_protein_BRAFLDRAFT_205831__partial__Branchiostoma_floridae_

SIAIVGLWCRFPHA-DSPDDFWRVLVNGEDC-MEEIPPDR-WSQDAWYSPDKN-APGKIFVQ-RAGFVQ----
GYKMFDPSLFGLRDEEASRMDPQQRFMLECSYKAFEN---AGIT---MDELHGSDTGVFVGLMNTEY-----LLDGDI--DLTQMTNHTITGTA-T--
SLVAARVAYTFNLTGPALTVDTACSSGLYAVHLGCQAIKTGDCSMAVCGGVSFILRPNMFVS--LCRA-----GMA-SPEGRCKPFSAA---A-DGYARGEG-

CGVVILKK--LSKALENNDH-IWGVIH-TAVNQDGRTTIP----ITAPSQSQQEKLLRSILERYR---FD-PDH----IDYIEAHGTGTPVGDPVEANSLGN-VI----
GKADDDPVLLGSVKGNIGHLESAAGLAGLIKVLLMMKYKQIVPNVHFD-RP-NPNID--FDHLRLYVPTEVLDWT--------LRG--
KDRRLACVNSFGFGGSNSHALVEQFPQVNKEQGKAYVVVVSARKRESLKLSVEDLIEHL-EQNPDISLERLAYTSTVRRT

>XP_002605916.1_hypothetical_protein_BRAFLDRAFT_87410__Branchiostoma_floridae_

PIAVVGIGCRMPGGTTSPSKFWDVISEGRNV-ITEVPAER-WSLDVYHSTDPH-QPGTHVTR-RAGFVD----
DIDMFDHTFFKISPREAAMMDPQQRHLLEVTYEAFED---AGIT---PESVSES-CGVFVGIGMMDY-----ALNMAD--DKHLFNSYANTGVA-H--
SVAANRISYAFNLKGPSLAVDTACAASMTALHLGCSSLRNKECSVAIVEGANVLLMPEVTVA--FSAM-----GVL-SPDGFSCPFDAS---A-NGYVRSEG-
FGAILLKP--LSDAIRDRDH-VYSVIRGSAISDNGFSQS-----ITMPSSGEQEKLMRTTYARFG---VP-LSS----VMYVEAHGTSTPVGDPAEAEAIGK-TF----
GRRPASPLKIGSAKSNFGHMECAAGMVGVIKTALMLDRRALCPSVNYT-KP-SPKAD--FDELGISVQTEVETFP--DN---K-------
KVTLGVNSFGFGGALAHVIMEENSAPSDQEGDFIILPLSGKSPDALRDLSAKWLTYK-DEKDA---INVSSWVATRRN

>XP_002608071.1_hypothetical_protein_BRAFLDRAFT_91451__Branchiostoma_floridae_

PVAIVGIGCRMPGGTTSPEKFWDVISEGRNV-ITEVPPER-WSLDVYHSTDPH-QSGT-HVTRKAGFVD----
GIDMFDHTFFKISPREAAMMDPQQRHLLEVSYETFED---AGIV---PEKVSES-CGVFVGIGMFDY-----AMGLAP--NRHLFNSYASTGLE-H--
SLAANRISFVFNLKGPSLTVDTACASSMTALHLACSSLWNRECNIALVGGCNTLLSPETTVG--FSAM-----GVL-SPDGRSCPFDAS---A-NGYVRSEG-
FGAILVKP--LKDAARDGDH-IYCVIRGTAIADNGFSQ-----SITMPSSSAQEILMKNTFARFG---VP-LSS----VSYIEAHGTSTPVGDPVEAQAIGN-TF----
GPHRRSPLKIGSCKSNFGHMECASGIVGVIKTALMMDRRTLCPSINFT-SP-NPEVD--FEALSVSVQTKLEPFVTNE------------
KMIAGVNSFGFGGSLAHVIMEQHIAPKDETGKYVILPLSAKTQDSLSDLAKKWLEFKDE----KDAVCVASWASTRRT

>XP_002610100.1_hypothetical_protein_BRAFLDRAFT_89867__Branchiostoma_floridae_

--AVVGIGCRMPGGTDTPSKFWDVIREGRNV-ITEIPPER-WSLEAYHSTDPH-QSGTHVTR-KAGFVD----
DIDMFDHTFFKISPREAAMMDPQQRHLLEVTYEAFED---AGIT---PESV-SESCGVFVGIGMMDY--AL-FMAG----DKHLFNSYVNTGIA-H--
SVAANRISYAFNLKGPSLAVDTACAASMTALHIACSSLRNKECSVALVGGSNVLLMPEVTVA--FSAM-----GVL-SPDGFSCPFDAS---A-NGYVRSEG-
FGAVLIKP--LSDAIRDRDH-VYSIIRGSAISDNGF--SH---SITMPSSSAQEQLMRTTYARFG---VP-LSD----VLYVEAHGTSTPVGDPAEAEAIGN-AF----GP--
ASPMRIGSAKSNFGHMECAAGMVGVIKTALMLDKRTLCPSVNYT-SP-TPKTD--FEELGISVQTKVENFP--DN---Q-------
IVKLGVNCFGFGGALAHVIMEEHNAPSDQEGDFIILPLSGKSSDALRDLSSKWLTFQ--DEKD--ALSVSSWVATRRN

>XP_003222100.1_PREDICTED__phthioceranic/hydroxyphthioceranic_acid_synthaselike__Anolis_carolinensis_

-IAITGIGCNFPGG-EGLDSFWTVLENGRNC-TTEIPPER-FNISEWYEPDNN-KPNKICTT-RAALLD----
EFNAFDHQLFGINKEEAEHIDPQQKLLLECTYRALED---AGVT---RENISDTKTGVFVGLKNRDY--ES-IASR----AVGKTTHYDGTGTA-S--
STAANRISHTFNLKGPSLDIDTGHSSFLSALHLGYKAIKQGDCEAALCGGVNCIIDPWKFAS--LSKA-----KML-SPDGITKPFSKK---A-TGYGRGEG-
CGVLLLKP--LMKAQEDLNK-IWGVLSITAVSQNGK---P----VTKLPQVEQEKLLLSIYPA-Q---IN-PSV----VQYIETHSIGNPAQDTAEAESLGN-II----
GTSNLPLLKIGSVMGNIGHTESAAGAAGLIKVLLMMHHGKIVPSLHFS-ES-NSSIN--TKKLNLLIPTTVEKWD--ES---S-----
EFGRVAGINCFGYEGNNIHAVVRQVRQKRPV----ELFIISAVSGCSLRQTMEDTARYV--KRNDINLQNLAYTSACRRS

>XP_003222101.1_PREDICTED__phthioceranic/hydroxyphthioceranic_acid_synthaselike__Anolis_carolinensis_

EVAIVGIGCNFPGG-EGLDNFWQVLVEGKNC-VQEIPPER-FDTTEWYDTDSN-KQGKTTTK-HAAFID----
EFNTIDNKLFGINDVEVEYMDPQQKLLLECTYRALED---AGVT---RENINGSTTGVFVGMMNQDY--RI-TRSR----TSCTINHYDGTGTA-M--
SIAANRISYTFNLTGPSIAIDTACSSFHYALHFALHAIKQGDCEAALCAGVNCIIEPRVFIS--LSKA-----KMI-SPEGISKPFSTN---A-DGYGRGEG-CAVLLLKP--
LKKAQEDFSK-IWGVISMSAVNQNGRSVTP----ITRPSQQEQENLLRRIYPV-H---VD-PTT----VQYVEAHGTGTPAGDPVEAESVGN-VL----
GKSNFPPIKIGSVKGNIGHTESAAGAAGLIKVLLMMHHGKIVPSLHFS-ES-TSSIN--TEKLNLSIPTTVEKWD--EP---S-----
EFGRIAGINSFGFGGTNAHVVVRQFKQKRTV----ELFLISSASVNSLKLTMEDMARYL--KTKDIALPNLAYTSACRRS

>XP_003222102.1_PREDICTED__probable_polyketide_synthase_1__Anolis_carolinensis_

EIAIVGIGCNFPGG-EGLDNFWQVLVEGKNC-VREIPPER-FDTTEWYDADSN-KQGKTTTR-HAALIE----
EFNSFDNRLFGINEVEAEYMDPQQKLLLECTYRALED---AGVT---RENINGSKTGVFVGMMNQDY--RL-LRSR----TSSTFSHYDGTGTS-M--
SISANRISYTFNLTGPSIAIDTACSSFHYALHIGLNAIKQGDCEAALCAGVSCIIEPGVFVS--LSKA-----KMI-SPEGISKPFSTK---A-DGYGRGEG-CGVLLLKP--
LRKAQEDFSK-IWGVISMSAVNQNGRSVTP----ITRPSQQEQENLLCKIYPA-H---VD-PST----VQYVEAHGTGTPAGDPVEAESVGN-VL----
GKSNFPPIKIGSVKGNIGHTESAAGAAGLIKVLLMMHHGKIVPSLHFS-ES-TSSIN--TEKLNLSIPTTVEKWD--EP---S-----
EFGRIAGINSFGFGGTNAHVVVRQFKQKRPV----ELFLISAASVNSFKLTMEDTARYL--KTKDIALPNLAYTSACRRS

>XP_004081384.1_equisetin_synthetase_eqxS__Oryzias_latipes_

GVAVVGIGCNFPGG-EGLENFWKVLVNGKNC-CVPIPRER-FDTRSWYDPDDT-KAGKSRTA-KAAFMD----
GFNEFDHRFFGISESEVEQMDPQQKQLLQCVYRALEN---AGIP---MEKASGTRTGVFFGMMNRDY----ETNAAHM--HPSVINHWTGTGLA-M--
SIAANRVSYVFNLTGPSLSLDCACSSSLVALHLACQSIKQGDCDMAFCGGVNCIIEPRVFVA--LSKA-----KMI-SPEGTSKPFSTQ---A-DGYGRGEG-
CGVVLLKP--LKRALQDLDH-IWGVITKTAINQDGRSVVP----MTKPSVKQQEELLRRIYSEQD------TAA----VQFIEAHGTGTPIGDPTEAESISN-VITKARPP-
GSQVLWVGSVKSNIGHTESTAGMAGLIKVLLMMKHKTIVPSVFYS----EKTSSVDVKALNIKIPQRVEKWE--CS---G-------
PRVAGVNNFGFGGTNAHAIVKQYLQQKSDEMPAKYFVMSAMSQKSLTMMVEDTLKHL-EEDRKVDLDSLLFTSACRRS

>XP_005144267.1_PREDICTED__fatty_acid_synthase__Melopsittacus_undulatus_

DVVIAGIAGKLPES-ENLQEFWENLLNGVDM---VTEDDRRWKPGMYGLPKRN-GKLK---------------
DISKFDASFFGVHPKQAHTMDPQLRLLLEVSYEAVLD---GGIN---PTTLRGTDTGVWIGASGSEA-----AEAFSQ--DPEKLLGYSMTGCQ-R--
AMLANRISYFYDFTGPSITIDTACSSSLIALENAYKAIRHGWCSAALVGGVNLLLKPNTSVQ--FMKL-----GML-SPDGACKAFDAS---G-NGYCRSEA-
AVVVLLTK--RSMAKR-----IYATIVNAGSNTDGFKEQG----VTFPSGDMQQQLVRSLYKECN---IR-LED----VEYVEAHGTGTKVGDPQEINGIVN-IF--HQCK--
RKPLLIGSTKSNMGHPEPASGLAALAKVILSLEHGLWAPNLHFN-TP-NPDIP-ALQDGTLEVVCKPTPVK--------------
GGLVCINSFGFGGANAHVILRPNENPLETFNIPRLVQICGRTQEAVETLIQESRKHGRC----SPFLSMLTDISTVPV

>XP_005153144.1_PREDICTED__polyketide_synthasenonribosomal_peptide_synthetaselike__Melopsittacus_undulatus_

EIAIVGIGCNFPGG-DGIDNFWKVLEEGRNC-TEEIPPER-FIVKEWYDPDEN-KPGKICTT-RAALLK----
EFNSFDNHLFGINNMEAERMDPQQKLLIECTFKALED---AGVP---VEAISGTNTGVFVGVMNRDY--EV-ITSR----IVIEMNHYDGTGSA-M--

SIIANRVSFTFNLTGPSLTIDTACSSFLFALHYALQTIKSGDCEAAICGAVNCIIDPRTFVS--LSKA-----KMI-SPDGLSKPFSKQ---A-DGYGRGEG-CGVLFLKP--LKKAKEDHSK-IWGVINISAINQNGKFATP----ITRPSQIQQENLLRRIYET-H---VD-PSV----VQYIEAHGTGTAAGDPTEAESLGN-VI----SKSEVSILKIGSVKGNIGHTESAAGAAALIKVLLMMHHGKFVPSLHYS-KE-MSSID--TEKLNLEIPTTVEPWE--ES---G-----EYGRVAAINCFGFGGTNAHVVVRQAKQKRPL----ELVLLSAASSKSLQMTMADTADQL--STRNITLPSLAYTSACRRS

>XP_005643614.1_hypothetical_protein_COCSUDRAFT_49000__Coccomyxa_subellipsoidea_C169_

-VDIMGFSCRFPES-ETPTQFWENLVAGTDM---VTENDRRWPVGLHGTPGRF-GKLP---------------EYHLFDAPFFSVHGKQASKMDPQMRKLLEVTHEAFVD---SGID---YKALRGSRVGVYLGCCGSEV-----HAMWLS--DYNNITGYEQTGCT-L--SMFANRLSFFFDFKGPSKAVDTACSSAFMALHDAVLDLQRGRVDYAVVGGSSAIFRPATSLA--FLRL-----KML-SPDGACKSFDAS---G-NGYARAEG-VASIILRRSDVFDTPIVIPREPYARVMGIGTNNDGHTEQG----ITFPSGPAQQELGTAVCRATG---ID-PSD----IQYVEAHGTGTVVGDSQELSAIDG-LYGAGAGHTPEEPLLIGSVKSNMGHCEGCSGLAGLIKVCLSYENGLIPGNLHYK-EP-NPNNE-SLKTGILKVVTGATPWQ---------------GGLVAISSFGFGGSNVHAIIAGCVREEPLFPPELIIPIVARTQEGSQSLLEAIKARA-FMADKLPLKRLANHLSDDVG

>XP_005646747.1_ketoacylsyntdomaincontaining_protein__Coccomyxa_subellipsoidea_C169_

----IGLSCRFPES-NGPAEFWTNLIDGTDM---LTTDDRRWPIAFQGLPPRS-GKAP---------------GFDKFDAAFFSVHGKQAQKMDPQLRKLLEVAYEAWVD---AGID---FRALRGSRVGVYVGSCGSEI-----HAQWVA--DIDSITGYEQAGCA-R--SMFANRLSWWFDFHGPSKCIDTACSSSLTAFNDAISDFEAGRIDYAMVGGTSGIFKPQTSIA--FQRL-----HML-SPDGTCKSFDAS---A-NGYARSDG-IAAVVLRRPGLSEAPVWAPRRPYARVLACATNNDGHTKEG----ITFPSGPAQKALAQQVCAKAG---LA-PSA----VGYIEAHGTGTVAGDGQELGAIEE-WYGRAGARTPDNPLLIGSVKSNMGHCEGCSGLAGLIKVLLSYEHRTLPGNLHFK-EP-NPSSA-GLRDGILKVITEPTAF----------------EGIAALSNFGFGGTNVHLLLQGGGWEQSRAATGKVIPLACRTQEGMAEVVKILQGLP-GGEESVVDPMRCWANSCGGS

>XP_006815698.1_PREDICTED__uncharacterized_protein_LOC100373061__Saccoglossus_kowalevskii_

AIAVVGIACRFPGA-NDLDEYWRVLSNGENH-LREIPKER-WNNDAFYSNDKK-EMGKNYVR-HAAFID----RYDEWDNRFFQINDDEAMRMDPQQNILLECVYKALEN---GGFP---VNELAGSNVGTYIG--DCAMAVCGGINLILSPTLFIS--LSRA-----RMA-SATGKCQPFSAD---A-DGYARGEG-CGVVVLKP--LQKAIKDGDI-IWGVI-GTGCNQDGHFATP----ITAPSGKQQEELLNLVYAKYK---VD-PSM----VTYIEAHGTGTPVGDPIEANSLGH-FIGQARSQ-GKPPCYVGSVKSNIGHLEAGAGVAGLIKVLLMMKYRKIPATLHFS-KA-NKKIN--FAELCLKVPTEFVDWK--------VNEGD--SLTACVNSFGFGGTNAHAIVTSYSKPRCSQEKSHIIALSAKTRESLILMIKHLKRSIQQDQP-PTLSALAYTSTVRRT

>XP_012938829.1_PREDICTED__lovastatin_nonaketide_synthaselike__Aplysia_californica_

--------ASVPGA-DNVEQFWRVLEQGENH-VVEIPKER-WNNDAFFDTDVH-AVGKSYVK-RAGLLK----DPKAFDNKLFNINDFEADQMDPQQRFVLECCFMAMED---AGIT---REQLAGSNTGVYVVGAMNGDY-----RALFTA--KSPIVGNYTVTGIS-N--SIISARVSFTFDLRGPSMTLDTACSSALVAIHVGAQALRTGDCDLALCGGTNFLMSPDVFVH--LSKA-----HMV-SPTGQCFAFSDS---A-DGYTRGEG-CGMVILKR--LKDALRDGDH-IWATI-ETGSNQDGHSVTP----ISAPSGDQQLRLLETVYQDAA---MD-LEG----IDYIEAHGTGTSAGDPVEAKALGT-FF------GTTRQRYIGSVKTNVGHLESAAGTAGLIKVLLMMKHSKIAPSLFFD-RP-NPKID--FPDLNLQVPTKAMDWP-------------RRNKIACVNSFGFGGTNCHAVVKAFVEQSEVLSIPCIVCFSAKHEKSLKGSIEDFVQHPEVSS--LDVHDVSYTSTVRRD

>XP_013068913.1_PREDICTED__probable_polyketide_synthase_16__Biomphalaria_glabrata_

AIAIIGIAFKGPGA-ENLEQLWEVLEGGKNY-VLDLPKDR-WNVDAFYDPDPQ-ATGKYYTR-KAGLLS----NPLDFDNTFFDINDIESDQMDPQQKFVLECSYRAMEH---AGIT---RRSIQGSKTGVYIGAMNCDY-----RGLFPV--GYSVVSNYTVTGVS-N--SIIAARVSYAFDLRGPCMVLDTGCSSALLAIHTASQALLNGDCTMAICGGTNFIGSPDIFIH--LSKA-----QMV-SPTGQCHAFSEQ---A-DGYTRGEG-CGIVLLKK--LSD---YE--------IEYIEAHGTGTQAGDYAEANALGK-FF------GSSKKRLIGSIKTNIGHLESAAGVVGLIKVLLMMQHNKFVPSLFSE-SL-NKRID--FEALNLVVASQVKDWT-------------TENKVGSVNSFGFGGSNCHAVVRSVVQTTNQVNNACVVCFSGKTVTSLMGSLRDLANYKRLKL--LDVRDIAYTSTCRRS

>XP_013068917.1_PREDICTED__mycocerosic_acid_synthaselike__Biomphalaria_glabrata_

EIAVIGVGFKGPGA-DNLEQLWMVLENGENK-ISEIPKDR-WNSDAFYDSDTE-ATGKYYSR-KAGLLS----NPHEFDCSFFNISEIESEQMDPQQKFVLECTYRAMEQ---AGIT---RDKIKGSKTGVYIGAMNCDY-----RGMFPA--RSAGVSNYTVTGIS-N--SIIASRVSFTFDLRGPCLVLDTGCSSALIAIHTASHALTLGDCSMAICGGTNFMATPDVFVH--LSKA-----QML-SRSGQCHAFSDQ---A-DGYTRGEG-CGIVILKK--LSDAERDNDP-ILAVI-TTGTNQDGHTVTP----ISAPSMSQQIQLLEDMYRDLS---LEQLDE----IEYIEAHGTGTQAGDYAEANALGQ-FY------GSSKKRLIGSIKTNIGHLESAAGAAGLIKVILMMEHNKIVPSLFSE-RP-NKRIN--FDDLNLVVPPKVREWS-------------TEKKVACVNSFGFGGSNCHAIVRSYKASRQCEKKMCIVCFSGKTVESLKGSLKDFLNFSCIEA--LDIHDVSFTSTVRRS

>XP_013079084.1_PREDICTED__phthiocerol_synthesis_polyketide_synthase_type_I_PpsClike__Biomphalaria_glabrata_

QIAIVGISCKVPGA-DNVEQYWKLLENGENH-VIDIPKER-WNNDAFYDPDPH-AIGKSYVR-RAGLLK----DPIAFDNKLFNINDFEADQMDPQQRYVLECTFRALED---AGIA---REKLAGSNTGVYVGAMNGDY-----RGLFTA--KSPIVGNYTVTGIS-N--SIIAARISFVFDLRGPSMTLDTACSSALLAIHIGAQALRTGDCDMALCGGTNFLMSPDVFVH--LSKA-----HMV-SPTGQCFAFSDS---A-DGYTRGEG-CGMVILKR--LKDALRDGDR-IWATI-ETGSNQDGHTVTP----ISAPSGDQQIKLLDQVYKNSG---VD-VGK----IDYIEAHGTGTSAGDPVEANALGK-FF------GAVRHRYIGSVKTNLGHLESAAGTAGLIKVLLMMKHSKIVPSLFFD-RP-NPKIN--FPDMNLSVPTQVVNWQ-------------SQNKVACVNSFGFGGTNCHAVVMEYEDMVEQKNIPCVVCFSAKHEKSLKGSIEDFVNHPDVST--LDVHDVSYTSTVRRD

>XP_013396153.1_fusarin_C_synthetase__Lingula_anatina_

AIAIVGIGCRFPGA-NNIDEFWQVLVNGENH-LIEIPEDR-WNSAAFYDPGRD-TPGKSYVK-QAAFVQ----HHDAWDNRFFMINDAEAERMDPQQAMVLECVYMALEN---AGIT---REDIFGSTTGVYLGIMNSDY-----SALVNA--DLVEINHHAPTGSS-N--SIAANRVSFTFNLQGPSLCVDTACSSAMVALHLAAQALRTGECTTAIAGGCNLIMTPENFVP--LCRA-----KMM-SAKGQCQAFCAD---A-DGYARGEG-CGMVIMKR--LKDALHDHDH-IWAVV-GTALNQDGHTAVP----ISAPSASQQETLLKHIYCKYQ---VD-PAE----LQYAEAHGTGTPIGDPIEVNALGK-VLGKSRKE-DDLPLFVGSVKTNIGHLESAAGCAGLIKVLLMMRAGKIAPSLHYTPERGNPKIN--FDEIRMKVPTEVTDWPPSPKG----------YRMCCVNSFGFGGTNGHAIVREHLNPEHGDDQTYIVAVSGTSEGSTQGTLTHLVERLRTGQ--YTLNEVAYTSTCRRT

>XP_013403200.1_phenolphthiocerol_synthesis_polyketide_synthase_type_I_PpsDlike__Lingula_anatina_

KVAIVGIGCRFPGEINNVKDFWEVLLKGIDC-TRPLPDDR-FDVNHFYHPTPK-TPGKLYVR-GGGYLEQ---
DLLSFDRLFFKMPPDEANHMDPQVRLLLEVVWEAFED---GGIP---ARSIRGTNCGVYMGVTASEY--TT-LTSA----RSDAISQYSNSGTN-S--
CMVSNRISYEFDLHGPSLSIDTACSSSLYAVHLASEAIRNGDCDMAVAGGVNVIMMPEITIG--FCQA-----GML-APDGRSKSFDSS---A-DGYCRSEG-
AGVIILKS--LNKAVEDGDR-IYAVIRGGALSNDGR--TP---GIANPSYSAQVNLVYRACRHAM---IN-PQD----IQYVEAHGTGTQVGDATEANALGK-TI----
GQAKT-PLYIGSVKSNIGHCEGSSGIAGVIKVALMIHNELIPPVVHFK-LG-NPKIK--FKELNIKVPKELIIWP--YV---EEM-----
PLIAGCSSFGFGGANAHVILEAPPS---------VVLISAATTTALSSRVDDFIELF--RKIQERFYDVAATTCLRSH

>XP_013405320.1_PREDICTED__phthiocerol_synthesis_polyketide_synthase_type_I_PpsClike__Lingula_anatina_

AIAIVGVGCRFPGA-DDIDEFWKVLENGENH-VIDIPKER-WNNDAFFSSDPD-APGKTYVT-RAGFVK----
GHIEWDNRLFGVNDLEAARMDPQQRFVLDCCYMALEN---AGIP---TPDIQGSRTGVYVGVMNNDF-----ADMFET--DVSSMNNYSLTGAS-N--
SITAARVSYVFNLLGPCMSVDTACSSAMVATHLGCQALKNGECNMALCGGVNAIMMPTMHVP--LSKA-----RMV-SPTGQSQAFSSA---A-DGYARGEG-
CGIVVLKR--LKDALKDGDR-IWAII-GTGVNQDGRTAHP----ITAPSGSQQAALLKEVFLRYH---VN-SDD----IQYVEAHGTGTQAGDPVECNAIGS-
FLDRKARV--APECYIGSVKTNIGHLESAAGVAGLIKVLLMMKHEKIVPSLHFKGRPANPKIN--FHELRLRVPTEVMNWTPPEGK----------
PRLACVNSFSFGGTNAHAVVMQFDRTEAFEKSPEIIAISAQNQWSLEQITKNLMEKVSNKE--YSLRDIARTSTCRRT

>XP_013759672.1_fatty_acid_synthase__Thecamonas_trahens_ATCC_50062_

KVVISGLSCRFPES-EDAETFFANLVAGTDM---VTADDRRWDMDTMDARSLPARSGK---------------
SIAEFDARFFAVTGKAADKMDPQLRLLLHATHEAVTD---AGWT---LDELAGSSTGVWVAGSFS-------DSHFDLLEDATAITGYEHTGCL-L--
NMFSNRISYFYDLHGPSLTVDTACSSGMVALAMALAALRSGECERAIVGGVNIQLIPTRSVA--FDKL-----NML-SPDGACKTFDAS---A-NGYVRAEG-
IAAIVLDA--ADTARN-----AYLDLLVAACNNDGYTPQG----ITYPSSDAQVALIRSCLAAAG---AT-PED----
VVYVEAHGTGTQAGDSVEANALDEVFVKHVLSRSPENPLLVGSVKSNMGHCENVSGLAGIIKVCLAARAGVLPPNLHYT-QP-NPKIP-
ALLSGTMRVVTQPTPWR---------------GGLVAISSFGFGGTNAHALVRA---PQPARAADDSADQSTDGLAALRAEVVQLLQSY-TGELAAALSDRLASLFGSAP

>XP_015783256.1_fatty_acid_synthase__Tetranychus_urticae_

DIVISGISGRFAES-DNIDELAKNLFDNVDM---ITEDDRRWPLGLYGLPT---RSGKIK-------------DLSKFDAQFFGVHGKQANLMDPQARMLLELTYEALCD---
AGVN---PHSLRGTRTGVYVGASVSEV-----EEGLAQ--DVSKVSGYALTGCS-R--
SMFANRISYTFDFQGPSYAIDTACSSSFLAFQQALLGLRSGQCDQAIVGGVSICLRPVTALQ--FHKL-----NML-SNEGKCKHMDAS---A-DGYVRSEA-
CSVVFLQR--KSAAKR-----IYATVIHSKTNTDGYKKEG----ITHPSRDAQRDLMKEVLEESG---IS-PTD----VNFVEAHGTGTKLGDPIEIDAIAE-VY----
CQGRTEPLLIGGVKSNLGHTEPASGLCSLAKVLIAFENKNIPANLHFN-TP-NPDIK-PLVRGQIKPIVENTPYN---------------
GGIAAINSFGFGGVNVHALIKSNDK---------VVTMCGRTEEGVNSFFDFIEKNP--TRVTKEFLALVSEVSKVPA

>XP_015792919.1_fatty_acid_synthase__Tetranychus_urticae_

TIVISGLAGRFPGA-DDVDQLWDKLLQGKVL----YSEERDRHPSKLRNRPTALGQIN---------------
DLSRFDAQFFGYDAKAAEHMGTQNRVLFEVVYEALWD---AGIL---VDSLRGSRTGFFLGISAG-----------------DGSDQYDLDETTDP--
VYIFSRLAYHFNWKGPCLSLDTACASTADAIIQAHWHIKHGLCDVAVVAGVNTIVDPFVNMT--AADM-----KML-SQSGFERCLDEN---A-DGFIRSET-
IAALILQR--KPAAKR-----IYGEILGTSVNNDGYTEEG----ITFPNRYAQADAMRSALKIAN---IS-PSQ----IDYIEAHITGTQAGDAVETSAILD-AY----
RDNSDQPIKVGCIKSNIGHAEASAGINAVIRACKILQTSHLSPNAAYS-VP-NSRIS-GLLEGKIIPVLEKQQFN---------------
ANILGVNTFGFGGSNGHIILKGNTQPLDSSLFPRLIVFCNRTRQGIEGMAQHLLTKD-ALNED--LIAMLNELSLAKP

>XP_018429938.1_PREDICTED__probable_polyketide_synthase_16__Nanorana_parkeri_

QIAIVGIGCNFPGG-EGIDNFWKVIIEGQNC-TVEIPKER-FDTKHWYDSDYN-KPGKICTS-RAAIVE----
GIHMFDNKLFGIHNSESDQMDPQHKLLLQCTYRALED---AGYP---MESIGGTETGVFIGLMNKDA--EF-IYNN----YPEKINHFNGTGTS-M--
CIAANRISYQFNLTGPSLSLDTACSSSLVALHLACQAIKQGDCELAICGGVSCILDPRIYVA--LSKA-----KMI-SPEGTSKPFSKK---A-DGYGRGEG-
CGIVVLKP--LKQAKEDYSK-IWGVICASSINQDGRSVTP----ITKPSLEQQERLLSRIYTA-----ID-PCS----IQYLEAHGTGTPVGDPIEVTSIGN-TI----
GKSKMKSLKIGSVKGNIGHTESAAGMAGLIKVLLMMHHEVIPPSLHYS-KE-LGIKQ--IEESKLVVPTAPEKWH--ED---V-----
KFGRMAGINCFGFGGTNSHVVVKQYKEKRPS----EILILSAASSKSLQLSIEDTKKEL-SKMPSICLENLVYTAACRRS

>XP_018430111.1_PREDICTED__LOW_QUALITY_PROTEIN__uncharacterized_protein_LOC108802755__Nanorana_parkeri_

AIAIVGMGCNFPGG-EGIDNFWKVIAEGKNC-TVEIPEER-FNTKHWYDSDDN-KPGKIRTS-RAAIVE----
GIHMFDNKLFGIHNSESDRMDPQHKLLLQCTYRALED---AGYS---MESISGSNTGVYIGIMNKDS--EF-IYNN----SPENINHFNGTGTS-M--
SIAANRISFQFNLTGPSLSLDTACSSSLVALHLACQAIRQGDCELAICGGVSCILDPRIYVA--LSKA-----KMI-SPEGTSKPFSKK---A-DGYGRGEG-CGIVVLKP-
-LKQAKEDYSK-IWGVICASSINQDGRSVTP----ITKPSQEQQERLLSRIYTT-----ID-PCS----IQYLEAHGTGTPVGDPIEATSIGN-TI----
GKSKMKPLKIGSVKGNIGHTESAAGMAGLIKVLFMMHHEVIPPSLHYS-KE-LGIKQ--IEESKLVVPTAPEKWH--ED---V-----
KFGRMAGINCFGFGGTNSHVVVKQXKEKRPS----EILILSAASSKSLQLSIEDIKQEL-SKMPSISLENLVYPAACRRS

>XP_026824077.1_fatty_acid_synthase__Ooceraea_biroi_

EIVISGIAGRYPDS-DNVELLKNNLFNKVDL---VTDDDRRWRFDHAEIPQRL-GKIN---------------NVGKFDALFFGVHFKQAHTLDPMCRMLLEHAYEAVID--
-AGIN---PRQLRGSRTGVFIGTCFSES-----EKTWFY--EKLQVNGFGITGCS-R--
AMLANRISYWLGIHGPSYTLDSACSSSLYALENAYRSILDGQCDSAIVGGSNLCLHPYVSLQ--YSRL-----GVL-SSDGTCKCFDAS---A-NGYVRSEA-
ICVLFLQK--AKDAKR-----IYSTVVHSKTNCDGFKEQG----ITFPSSVMQSALLRDCYEESG---MS-PSS----VSYVECHATGTKVGDPEELNTIEN-IF------
DRTTPLLIGSVKSNIGHTEPVSGLCQITKVVIAMETGTIPPNLHYK-TPLKGMKS--LEDGRLKVVTDTTPWE---------------
GGYVGINSFGFGGANCHVILKSHTKGAKTDDLPRLVVVSGRTEESVKVFLNDVERHAND----VEYVRLLHDIHADDI

>XP_324222.1_hypothetical_protein__Neurospora_crassa_OR74A_

-IAIIGMSCRFAGDVDSPSKLWDLLAQGKSA-WSEIPKDR-FNIDGFHHPNFEKLNGT--NVIGGHFMKE---
DVGLFDAHFFNLSAETAAALDPQFRLQLESTYEALES---AGIT---LQDVAGSNTSVYAGSFFRDY-----HESLIR--DPDTLPRFLLMGTG-A--
AMASNRLSHFFDLRGPSMSVDTGCSTTLTALHQACQSLRSGESTMSIVGGANIMFNPDMFLA--MSSM-----TLI-SKDGRSWAFDSR---A-NGYGRGEG-
SATVVLKP--LDAALRDGDP-IRAVIRDSGINQDGKTET-----ITTPSGEAQEALIRACYERAG---LD-PGQ----
TTYFEAHGTGTPTGDPIEVKAIARVFKDSRKGNGEDALLRIGSVKTNIGHTETASGVAAIIKVALALERGQIPPSINFE----
TPNAKLSLDEWKLKVPTELEEWVGKDG-----------
IRRASINNFGYGGSNAHVIMEDYSSNNGDHHHSKVFLLSAKDEKATERMIANLKTYLHQQKANIAENALLSNLAHT--

>XP_324368.1_hypothetical_protein__Neurospora_crassa_OR74A_

-VAIVGMSCRLPGGVETPGDFYRMMCRGRSG-WSKVPKDR-FNQEAYNHPDPD-HKGTFNSQ-GGYFIKQ---
DLSGFDAAFFDVTRREAEAMDPAQRLLMECTYEALES---GGIP---REHIAGTRTGVFIGGNYGEH-----RFSHIR--DLDTIPSFDATGNQ-P--
AFLSGRLAYYFNLHGPTFTVDTACSSSLHALHLAVQSIRNGECDAAVVGASHLITQPDVWVS--MSML-----RLF-SDEGRTYAFDHR---ARSGYARGEG-
CAVIILKP--VEKAFKDNDH-IFSVISHSGVSHNGRTV-----GIVAPSPDEQEQLLRDVFTAAK---ID-PRE----VGFFEAHGTGTKKGDPIEATAIYK-AVGRYL--
SADDPLYIGSSKPNVGHLECASGLVSVIKGVLSLYYGFILPNADFD-KE-NPGIP--FKEWNMTVAKQQKPWP--AH----------
KKYACVNNFGFSGSNSTCILAGPPIEHGAYTTPRLFVLSANDEQALRTSIKELGIWL--EQHAM-PRNLAYTLCQRRS

>XP_325868.1_hypothetical_protein__Neurospora_crassa_OR74A_

PIAIVGMACRMPGNVSTPAEFWELCTRARSG-WSETPKQR-FNSARFHHPNQG-KGGTLNPV-GGNFLNV---
DLAAFDAPFFGLTEKEAISMDPQQRLLLECTFEALEN---AGIP---KHTIAGKDVGVFVGGSFPEY-----ESHLFR--DPDTIPMHQATGCA-Y--
AMQSNRISHFFDLRGPSFTADTACSSSMVAIHLACQSLRTGESSAALVGGCHLNMLPEFWIS--FSTC-----RLL-SDAGRSFSFDNR---G-TGFGRGEG-
CGMIVLKP--LDQAIKDKDP-IRAVIAATGLNQDGKTP-----GITVPHGPAQ------VYGRAG---LD-PNL----CGFVEAHGTGTRVGDPIEATAIHN-VL----
GQSPRNPLWIGSVKSNIGHLEGASGIAGVIKAALMLERGFILPNHDFK-QP-NPRIP--WKEWNLEVPVTQRPWP------------
RGKKYISVNNFGFGGTNGHVVLTAAPFSDKSGLGRKLYVVTAHDKAALSQQMKNIVVYLEQRPEIDLTSNLAYTLGQRRS

>XP_329445.1_hypothetical_protein__Neurospora_crassa_OR74A_

PIAIVGSSCRFTGEATSPAKLWELLKDPKDL-TREVPKDR-FNVEGFYHPDGE-YHGTTNSA-KAYFLEQ---
DHRLFDASFFNITPKEAEAIDPQQRMLLEVVYEALES---AGYT---LQDYSGKKVAVFAGVMTADY-----DTLSQR--DDLSVSQYYATGNA-R--
SIISNRVSYFFNFHGPSMTIDTACSSSLVALHQAVLSLRSGEAEMACVSGVNLILTPEQFVV--ESSL-----HML-SPTGRCHMWDDR---A-DGYARGEG-
VAAIFIKP--LSKALADGDR-IEAIIRETGVNSDGRSK-----GITMPNWEAQSALIQDTYRRSG---LN-
ARDPIDRCQFFEAHGTGTAAGDPNEARAIEDAFFGRNVSTAEVSKLLVGSVKTVIGHTEGAAGLAGLFKVVHAMINGTVPPNLHFN-RL-QPAVA--
KYYSHLHVPTKALAWP--SV---AAGQ----PRRATVNSFGFGGTNSTAICEMYVPLSADGQTRLPLVLSASSPRSMGAVAKCYRDYL-AQVS-
LPLEEAAWYAYKNRT

>XP_330106.1_hypothetical_protein__Neurospora_crassa_OR74A_

PIAIIGMGCRLPGGSHSSSKLWELLKAGRTA-QSRFPPSR-FNIDGFYHPNSD-RPGSLNME-GGYFIED---
DIRGFENSFFGINNLEATYMDPQQRKLLEVVFETFEN---AGFT---LDQVSDANIGCYVGNFVTDF-----ITMQLK--DSEYTHRYSATGLG-T--
TILANRISHVFNMKGPSFVIDTACSSSLYCLHAAVAALIAGECDSAIVAGANLIQSPEQQLA--TMKA-----GVL-SKTSTCHTFDSS---A-DGYGRADG-
IGAILVKR--LSDAIRDGDP-IRSVIRGTAINSNGKTN-----GITLPSADGQEAVIRKAYAQAG---LG-FNE----TDYIECHGTGTAVGDPIEVEAVSR-VF----
KKPQGAPLLIGSVKSNLGHSEAASGLSSIIKVAMALEKGEIPPTYGVK-NI-NPKIK--TDEWNVQIVTETTPWP--KN---
LPHNAGRLFRRAGVNSFGYGGANAHAILEAPQMPASLTRSTLFLPFSGSNTAALERRVTDIAAAI--DFENVNIADLAYTLGVKRT

>XP_330288.1_hypothetical_protein__Neurospora_crassa_OR74A_

PVAIVGMACRWPGGVHDPSQFWEFLRNKVNG-WKEFDDPR-FSSAGFHHPNSD-RPGSMSMK--GAFLAEQ--
DARLFDHTFFGMTGLEVETMDPSQRKLLEVAYEAIES---AGET---WESVSGTRTGVFVGNFCLDH--WM-IQSR----DWDNPRPYAFTGAG-T--
SILANRISYIFNLQGPSLTVDTACSSSMYALHLAVNAIRAGDCDSAIVASANWIADPGVQIA--LDKL-----GAL-SASARCHTFDAR---A-EGYARGEG-
FGAIYLKR--PSLAIADMSP-IRAMIRGTAINSNGR--TG---GITRPSANGQETVIREAYRNAGN--LP-FRD----TSYFECHGTGTYVGDPIEVAALGR-VF----
APSSDDPLLIGSVKSNVGHGEGASALASIMKVVLALEHGAIPPIYDLQ-TR-NPNID--FEGAKVQPVTEVTPWP--KD---R-------
LQRASINSFGYGGANGHCIIDHVNIPNANTRPLVLLPLSAHNENSLELNLKALSQVV------LPLADVAYTLGARRS

>XP_682975.3_probable_polyketide_synthase_1_isoform_X2__Danio_rerio_

DIAIIGIGCNFPGG-EGVDNFWKVLVEGRNC-VVQIPDER-FETSEWFHPDEG-EPGKTRTT-TAALIE----
GFNEFDQKFFGISEAEADYMDPQQKLLLQCAYRALED---AGIP---LEKVSGTRTGVYIGLMNRDY--ET-LLNN----SPSTITHYNGTGTA-M--
SIAANRISYIFNLTGPSFAIDSACSSSLVALHSACQAIRQGDCEMALCGGVSCILEPRVFVA--LSKA-----KMI-SPEGTSKPFCRT---A-DGYGRGEG-CGIVLLKS-
-LKKAIEDFDH-IWGIVNKTAVNQDGHTVTP----ITKPSMTQQEALLRMIYSS-E---DF-LAN----VQYVEAHGTGTPAGDPVEAGSLSR-VI----
AKPSLGPLFVGSVKSNIGHTESAAGVAGLIKILLMMKHETIVPSLFYS-AE-NSSID--AKALNLKIPTRAEKWL--YS---G-----
SRIKMAGLNSFGFGGTNAHAIITEYVQLEPL----KLLPLSAATDQSLQLCIADTYQRI--SADEVDLQALAYTSACRRS

>XP_793564.2_PREDICTED__probable_polyketide_synthase_1__Strongylocentrotus_purpuratus_

PVAVVGIGTRHACGANTTDDFWKVLKEGKEC-ILDIPPER-WAIDNFHDEDQT-RQGKMVTK-RCGLID----
DLEGFDNLFFKISPREAASLDPQQRHLLEVNYEAFED---AGIN---PDNLGES-CGVFVGIGMMDH------AIQLV--DTSTTDAYTLTGIA-H--
SVSANRISYAFNLKGPSFAVDTACASGLTALHLACTSLWNRECSVALMSACNGIQLPDITVG--FSAL-----GVL-SPDGRCSPFSST---A-NGYVRSEG-
WGAIVLKP--LSQALADNDH-IYTVIRGSAIAANGLANS-----LTMPSPPAQEYVMKEAYEKFG---VS-MSD----VHYVEAHGTGTMVGDPLEAEAISR-AF----
NRTKDNPLKIGSVKSNFGHTEVAAGVTAAIKVALMMENRTIPPTINFV-SS-NPHID--PEEMKLDIVTNVQPFP--TE---D-------
KHIIGLNSFGFAGALAHCIFEEAPKDSDKEGQPIIIPLSAKSPEALTAVAKQWQNLD-IDQDA—MSAVSWMSTRRRV
