# Supplementary material for: De novo biosynthesis of simple aromatic compounds by an arthropod (Archegozetes longisetosus)
Source: Proc Biol Sci. 2020 Sep 2;287(1934):20201429. doi: 10.1098/rspb.2020.1429 (PMC7542773; doi:10.1098/rspb.2020.1429)
Supplement: Table S3 [file rspb20201429supp8.pdf]

Table S3. Relative composition of *A. longisetosus* defensive gland exudates, quantified based on the ion abundance of each individual compound per sample.

treatment	2,6-HMBD [%]	Neral [%]	Nerylformiat [%]	Tridecan [%]	γ-Acaridial [%]	7-Pentadecen [%]	Pentadecan [%]	Heptadecadien [%]	8-Heptadecen [%]	Heptadecan [%]	aromatic [%]	terpenes [%]	hydrocarbons [%]
all	4.62	5.83	45.18	7.49	16.07	3.25	11.52	0.00	5.74	0.29	20.69	51.02	28.29
all	3.87	6.33	43.47	6.17	15.61	4.19	12.94	0.00	7.42	0.00	19.48	49.80	30.72
all	5.48	7.12	36.40	5.89	19.66	3.63	11.85	0.00	9.33	0.64	25.14	43.52	31.34
all	2.76	35.69	21.56	7.90	26.10	0.00	2.23	0.00	2.10	1.67	28.86	57.25	13.89
all	0.00	0.00	59.90	6.00	7.55	4.42	9.46	0.00	10.70	1.97	7.55	59.90	32.55
all	5.38	7.81	45.28	5.22	17.35	3.01	8.95	0.16	6.44	0.39	22.74	53.08	24.18
all	0.00	0.00	49.81	7.72	6.11	4.91	14.21	0.00	14.20	3.03	6.11	49.81	44.08
all	7.50	9.30	40.33	5.24	19.10	3.27	8.64	0.15	5.97	0.49	26.60	49.63	23.76
all	3.11	1.58	50.41	7.19	14.00	3.18	13.06	0.18	6.78	0.51	17.11	51.99	30.90
all	0.00	0.00	0.00	0.00	0.00	0.00	0.00	0.00	0.00	0.00	0.00	0.00	0.00
all	0.00	0.00	50.68	10.29	6.20	5.47	10.91	0.00	12.95	3.50	6.20	50.68	43.12
all	6.05	9.39	47.62	6.42	10.94	2.79	8.41	0.00	7.42	0.96	16.99	57.01	26.00
control	7.33	18.79	30.97	4.13	18.91	2.44	11.07	0.12	5.98	0.25	26.24	49.76	24.00
control	3.75	0.00	57.46	3.10	13.00	3.38	9.53	0.00	8.75	1.03	16.75	57.46	25.79
control	0.00	0.00	62.52	1.47	9.46	4.08	10.53	0.00	10.16	1.80	9.46	62.52	28.03
control	0.00	0.00	0.00	0.00	0.00	0.00	0.00	0.00	0.00	0.00	0.00	0.00	0.00
control	8.17	7.02	45.55	5.13	9.98	4.15	11.09	0.00	7.84	1.07	18.15	52.57	29.27
control	0.00	0.00	59.72	0.00	0.00	5.38	12.42	0.00	16.01	6.46	0.00	59.72	40.28
control	0.00	0.00	63.73	1.23	9.00	3.64	11.14	0.00	9.77	1.50	9.00	63.73	27.28
control	4.74	5.12	46.62	2.77	16.01	3.46	10.65	0.00	9.42	1.22	20.75	51.74	27.51
control	7.95	15.90	36.34	3.34	12.98	3.69	8.55	0.23	10.05	0.96	20.93	52.24	26.83

control	0.00	0.00	65.27	2.43	8.75	2.44	10.23	0.00	8.59	2.28	8.75	65.27	25.98
control	7.44	20.31	32.97	3.99	14.23	2.87	10.38	0.00	7.53	0.29	21.66	53.28	25.06
control	0.00	0.00	64.62	2.19	5.39	4.28	8.81	0.00	11.27	3.45	5.39	64.62	30.00
control	4.68	0.00	48.17	6.45	5.30	5.19	15.28	0.00	12.72	2.22	9.98	48.17	41.85
control	0.00	0.00	65.28	0.00	12.94	2.56	9.00	0.00	8.84	1.38	12.94	65.28	21.78
control	4.62	3.14	52.97	2.80	8.93	4.59	11.20	0.28	10.27	1.20	13.55	56.11	30.34
control	0.00	0.00	56.78	0.00	0.00	6.17	11.21	0.00	16.31	9.53	0.00	56.78	43.22
control	8.00	32.97	26.26	5.06	0.00	3.30	13.69	0.48	9.81	0.41	8.00	59.24	32.76
control	8.77	35.07	27.02	3.96	0.00	3.30	11.88	0.54	8.84	0.61	8.77	62.09	29.14
control	9.39	30.38	24.71	3.73	0.00	3.78	12.07	0.91	13.86	1.18	9.39	55.08	35.53
control	11.30	26.32	28.41	4.49	3.77	3.64	11.14	0.46	9.72	0.75	15.07	54.73	30.21
