# Supplementary material for: De novo biosynthesis of simple aromatic compounds by an arthropod (Archegozetes longisetosus)
Source: Proc Biol Sci. 2020 Sep 2;287(1934):20201429. doi: 10.1098/rspb.2020.1429 (PMC7542773; doi:10.1098/rspb.2020.1429)
Supplement: Table S4 [file rspb20201429supp9.pdf]

Table S4. Bacterial to host copy ratio for untreated (control) and antibiotic-treated mites as assessed by qPCR

treatment	Ratio Bact/Host
control	7534.38
control	12452.43
control	31055.08
control	11531.81
control	713.96
antibiotics	3311.72
antibiotics	1418.65
antibiotics	2763.40
antibiotics	2302.05
antibiotics	621.37
antibiotics	417.31
antibiotics	2155.03
antibiotics	119.20
